# Sex differences in global burden of Congenital Heart Anomalies in children under five from 1990 to 2021

**DOI:** 10.1371/journal.pone.0348351

**Published:** 2026-05-06

**Authors:** Huasheng Lv, Fengyu Sun, Wentao Ma, You Chen

**Affiliations:** 1 Department of Cardiology, First Affiliated Hospital of Xinjiang Medical University, Urumqi, China; 2 Department of Pediatrics, Xinjiang Medical University, Urumqi, China; 3 School of Basic Medical Sciences, Xinjiang Medical University, Urumqi, China; Children’s Hospital of Los Angeles / Keck School of Medicine, UNITED STATES OF AMERICA

## Abstract

**Background:**

Congenital heart anomalies (CHA) significantly contribute to childhood morbidity and mortality worldwide. Understanding sex-specific differences and their association with societal development levels is crucial for formulating effective health strategies.

**Methods:**

We extracted sex-stratified incidence, mortality, and disability-adjusted life years (DALYs) for CHA among children under five from the Global Burden of Disease Study 2021 for 204 countries and territories (1990–2021). Sex differences were quantified using male-to-female rate ratios with 95% uncertainty intervals. Temporal trends were evaluated using the estimated annual percentage change (EAPC) derived from log-linear regressions. We assessed the association between disease burden and development status using the Sociodemographic Index (SDI). To address confounding variables and geographic clustering, we fitted linear mixed-effects models with sex, SDI, and calendar year as fixed effects and GBD region as a random intercept, reporting adjusted coefficients with 95% confidence intervals.

**Results:**

From 1990 to 2021, the global CHA burden declined. While descriptive analysis showed higher raw point estimates for males, a multivariable mixed-effects analysis—adjusted for SDI (as a proxy for macro-level development and health-system context) and temporal trends—confirmed that male sex was significantly associated with a higher CHA burden (DALYs Adjusted Coefficient: 876.4, P < 0.001; Mortality Adjusted Coefficient: 9.7, P < 0.001). This suggests a robust male disadvantage independent of socioeconomic status. The highest CHA burdens were observed in Sub-Saharan Africa, Southeast Asia, and South Asia, while improvements in SDI were significantly associated with overall reductions in burden.

**Conclusion:**

Despite overall reductions in CHA burden, profound regional disparities and observable sex differences persist, especially in resource-limited areas. Policy interventions focusing on gender-sensitive resource allocation, enhanced neonatal screening, and improved surgical access are critical to mitigating these disparities and advancing global pediatric health equity.

## Introduction

Congenital Heart Anomalies (CHA) are among the most prevalent birth defects in children, and their global disease burden is a significant concern [1]. CHA encompasses a spectrum from mild lesions (e.g., small septal defects) to critical malformations that are life-threatening in infancy, making them a leading cause of infant and childhood morbidity and mortality attributable to birth defects. Globally, CHA affects 13 million individuals and causes over 210,000 deaths [2], particularly in children under five. The mortality rate in this age group serves as a critical indicator of the efficacy of national health systems [3] and is directly aligned with the core objective of reducing child mortality as outlined in the United Nations’ 2030 Agenda for Sustainable Development [4]. Despite recent breakthroughs in diagnostic and therapeutic technologies—such as high-resolution echocardiography and minimally invasive interventions that have significantly improved prognoses—many children with CHA in low- and middle-income countries lack access to care and die before age five [5]. Furthermore, children with CHA face an increased risk of developing chronic heart failure and cardiovascular diseases in the future, imposing a significant burden on affected individuals and their families [6,7].However, this overall burden is not uniformly distributed and can be influenced by various factors, including sex. A deeper understanding of sex-specific differences is crucial, as it may reveal disparities in healthcare access, treatment responses, or underlying biological vulnerabilities that are otherwise obscured in aggregated data.

Current research still lacks a comprehensive understanding of the sex-specific differences in CHA and their dynamic association with societal development. Although existing literature has systematically described the epidemiological characteristics and regional distribution of CHA [8], the mechanisms underlying differences in incidence rates, treatment responses, and long-term outcomes between male and female patients remain unclear. From a biomedical perspective, differences in gene expression related to sex chromosomes may influence the regulation of key pathways in cardiac development. From a health systems perspective, gender-based cultural preferences may lead to delayed diagnosis or unequal allocation of treatment resources. Concurrently, the relationship between the Sociodemographic Index (SDI)—a composite measure of education, income, and fertility—and the evolving burden of CHA over time and space requires quantification. Particularly in low- and middle-income regions, it is essential to determine whether improvements in SDI are accompanied by enhanced early screening coverage and how such improvements impact the dynamic changes in sex-specific differences, necessitating multidimensional data modeling.

Accordingly, using GBD 2021 estimates from 1990 to 2021, this study had two overarching aims: (1) to quantify the global, regional, and national burden of CHA among children under five and characterize how this burden has changed over time; and (2) to determine whether meaningful sex differences exist in burden levels and temporal trends. Specifically, we (i) described sex-stratified incidence, mortality, and DALYs; (ii) summarized long-term temporal trends using estimated annual percentage change (EAPC); and (iii) examined the association between development context (SDI) and CHA burden and tested whether male–female differences persisted after adjusting for SDI and secular time trends in a mixed-effects framework. Clarifying these objectives is essential because temporal changes may reflect shifting diagnostic coverage, access to surgery, and broader health-system development that vary substantially across settings.

## Methods

### Data sources and definition

The data for this study were sourced from the GBD 2021 database, provided by the Institute for Health Metrics and Evaluation at the University of Washington, USA, and freely accessible via the Global Health Data Exchange (https://ghdx.healthdata.org/gbd-2021/sources). This database contains information on the incidence, mortality, and 88 risk factors for 371 diseases and injuries across 204 countries and territories globally from 1990 to 2021 [9]. Detailed information on the data, methodologies, and statistical modelling is available in previous reports [10].

The GBD classification system establishes a comprehensive and systematically organized taxonomy of health conditions. CHA are categorized within the primary classification of non-communicable diseases at the first hierarchical level, further classified under other non-communicable conditions at the secondary level, and specifically delineated under congenital birth defects at the tertiary level. In the GBD database, CHA were diagnosed according to the International Classification of Diseases, ICD-10, within the coding range Q20-Q28.9 (S1 Table). The detailed cause-ICD code mappings for GBD 2021 are accessible through the GHDx repository (https://ghdx.healthdata.org/record/ihme-data/gbd-2021-cause-icd-code-mappings). Data on the disease burden of CHA in children under 5 were extracted using the GBD Results Tool.

This study was based on publicly available, de-identified, and aggregated data from the GBD 2021 study. Accordingly, the Ethics Committee of the First Affiliated Hospital of Xinjiang Medical University exempted this study from formal ethical review, and the requirement for individual informed consent was waived. The overarching GBD study protocol was approved by the Institutional Review Board at the University of Washington. This study was reported in accordance with the GATHER (Guidelines for Accurate and Transparent Health Estimates Reporting) guidelines (S2 Table).

### Sociodemographic index

The SDI is a composite metric used to evaluate a region’s developmental level and its relation to population health. Specifically, the SDI is calculated as the geometric mean of three normalized indices, each ranging from 0 to 1: total fertility rate among individuals under 25 years of age, average educational attainment for those aged 15 and older, and lag-distributed income per capita (a metric accounting for income trends over time). In the GBD 2021 study, SDI values were rescaled to a range of 0–100 by multiplying by 100. On this scale, 0 corresponds to the lowest income and educational attainment levels combined with the highest fertility rates, while 100 represents the highest income and educational attainment alongside the lowest fertility rates. In GBD 2021, 204 countries and territories were stratified into five SDI quintiles: low, low-middle, middle, high-middle, and high [9] (S3 Table).

In the GBD framework, the Socio-demographic Index (SDI) is a standardized, time-varying summary indicator of development (income per capita, educational attainment, and fertility) that is strongly correlated with population health outcomes and is routinely used to contextualize cross-location differences in disease burden and expected levels across the development spectrum [1,11]. Because comparable country–year measures of specific health-care infrastructure (e.g., pediatric cardiac surgical capacity) and policy changes are not consistently available for all 204 locations over 1990–2021, we used SDI as a parsimonious proxy for macro-level socioeconomic and health-system context in cross-country ecological analyses, consistent with prior GBD-based burden studies that stratify or model outcomes by SDI [12,13]. We acknowledge that SDI does not directly capture specific policy changes or discrete health-system interventions. Therefore, in the multivariable models we additionally adjusted for calendar year to account for broad secular changes over time, including gradual improvements in diagnosis, treatment availability, and service delivery.

### Disability-adjusted life years

DALYs are a standardized metric used to quantify disease burden, representing the total number of healthy life years lost due to a disease from its onset until death. DALYs encompass two components: years of life lost (YLLs) due to premature mortality and years lived with disability (YLDs) due to non-fatal health outcomes [14]. This relationship is expressed by the formula:


DALYs = YLLs + YLDs.

Uncertainty in the estimates is represented by 95% uncertainty intervals (UIs), which are defined by the 2.5th and 97.5th percentiles of the distribution derived from computational draws. Uncertainty was systematically propagated through each step of the estimation process to ensure robust and reliable results.

### Estimated annual percentage change

The estimated annual percentage change (EAPC) is a commonly used summary metric to quantify long-term temporal trends in epidemiological and disease-burden indicators, and it has been widely applied in GBD-based and other population-level trend analyses [15–21]. In this study, we employed the EAPC to evaluate the dynamic trends in DALYs, incidence, and mortality rates associated with CHA from 1990 to 2021.

The EAPC was calculated by fitting a regression model to the natural logarithm of the rates, with time as the independent variable [22]. Specifically, the natural logarithm of each observed rate (y) was expressed as a linear function of time (x):


y=α+βx+ ε

where α is the intercept, β is the slope, and ϵ represents the random error term. This model assumes that the natural logarithm of the rate follows a linear trend over the specified time period, and that the random error (ε) follows a normal distribution. The EAPC was then derived from the slope (β) using the formula:


EAPC = 100 × (exp(β) − 1), where exp denotes the exponential function.

The 95% confidence intervals (CIs) for the EAPC were derived from the fitted model. Trend interpretation was based on the 95% CIs: a lower limit greater than 0 indicates a significant upward trend, while an upper limit lower than 0 suggests a significant downward trend. If the 95% CIs include 0, it implies no statistically significant change in trends over the study period [23].We explicitly acknowledge that this EAPC model assumes a constant, log-linear trend over the specified time period, which serves as a summary metric for the overall rate of change but may not capture short-term fluctuations or inflection points.

### Statistical analysis of sex disparities

To quantify the magnitude of sex differences (effect size), we calculated the Male-to-Female Rate Ratio (RR) with 95% uncertainty intervals (UIs) for incidence, mortality, and DALYs. An RR greater than 1.0 indicates a higher burden in males, while an RR less than 1.0 indicates a higher burden in females. The 95% UIs were derived to assess the statistical significance of these ratios; if the interval includes 1.0, the difference is considered not statistically significant.

The primary GBD outputs (incidence, mortality, and DALYs) and the EAPC calculations are descriptive summaries intended to characterize burden levels and long-term temporal trends. These descriptive approaches are not designed for multivariable adjustment of macro-level covariates or for accounting for correlation among countries within the same geographic region. Therefore, to address confounding at the macro level, we performed a separate inferential analysis by fitting a linear mixed-effects model. The model treated the DALY rate or mortality rate (analyzed in separate models) as the dependent variable. In this model, sex, SDI, and calendar year were included as fixed effects. Calendar year was included to account for secular changes over time, such as gradual improvements in diagnosis, treatment availability, and broader health-service development that were not consistently measurable across all countries.GBD region was specified as a random intercept to account for spatial clustering and unmeasured region-level factors. The association of sex was reported as the adjusted coefficient (β) with 95% confidence intervals (CIs). A two-sided P-value < 0.05 was considered statistically significant.

In this study, data cleaning, computational analyses, and graphical visualizations were conducted using R software (version 4.4.2).

## Results

### Global and regional level

From 1990 to 2021, the global burden of CHA among children under five exhibited observable sex differences and temporal trends. In 2021, the global DALYs rate for males was 1.21 times higher than for females (Male-to-Female Ratio [M:F Ratio]: 1.21, 95% UI: 0.84–1.75; 3087.77 vs. 2545.96 per 100,000 person-years, respectively) (S4 Table). A similar trend was observed for mortality, where the global rate in males was also 1.21 times higher than in females (M:F Ratio: 1.21, 95% UI: 0.84–1.76; 33.92 vs. 27.94 per 100,000 person-years) (S5 Table). In contrast, the difference in global incidence was minimal (M:F Ratio: 1.01, 95% UI: 0.71–1.43; 350.61 vs. 348.33 per 100,000 person-years) (S6 Table).

This pattern of higher male point estimates in disease burden was observed across most regions. For instance, in Oceania, the male DALYs and mortality rates were 1.29 times higher than those for females, representing one of the larger rate ratios observed (S4 Table, S5 Table). Sex differences in incidence varied by region; for example, male incidence was higher in both East Asia and Oceania, while in some other regions like Central Europe, the female rate was slightly higher (S6 Table). Temporal trends revealed a decline in DALYs and mortality rates globally and in most regions, with males experiencing a slightly faster decline than females (global DALYs EAPC: males −2.70% vs. females −2.28%; mortality EAPC: males −2.62% vs. −2.27%) (S4 Table, S5 Table). Changes in incidence were minimal, with a slight downward trend observed globally (male EAPC −0.30% vs. female EAPC −0.24%) (S6 Table).

### Global and national level

Between 1990 and 2021, the burden of CHA among children under 5 exhibited sex differences at the global, regional, and national levels. The geographic distribution of DALYs, deaths, and incidence was uneven, with Sub-Saharan Africa, Southeast Asia, and South Asia consistently showing the highest burdens (S7–S9 Tables). The spatial patterns were similar for both males and females. However, the point estimates for burden, mortality, and incidence were frequently higher in males across most high-burden regions. For instance, in high-burden areas like West Africa, male DALYs and mortality rates were higher than those for females, while high-income regions like North America and Europe consistently reported the lowest burden for both sexes.

### Time trend

**Fig 1:**
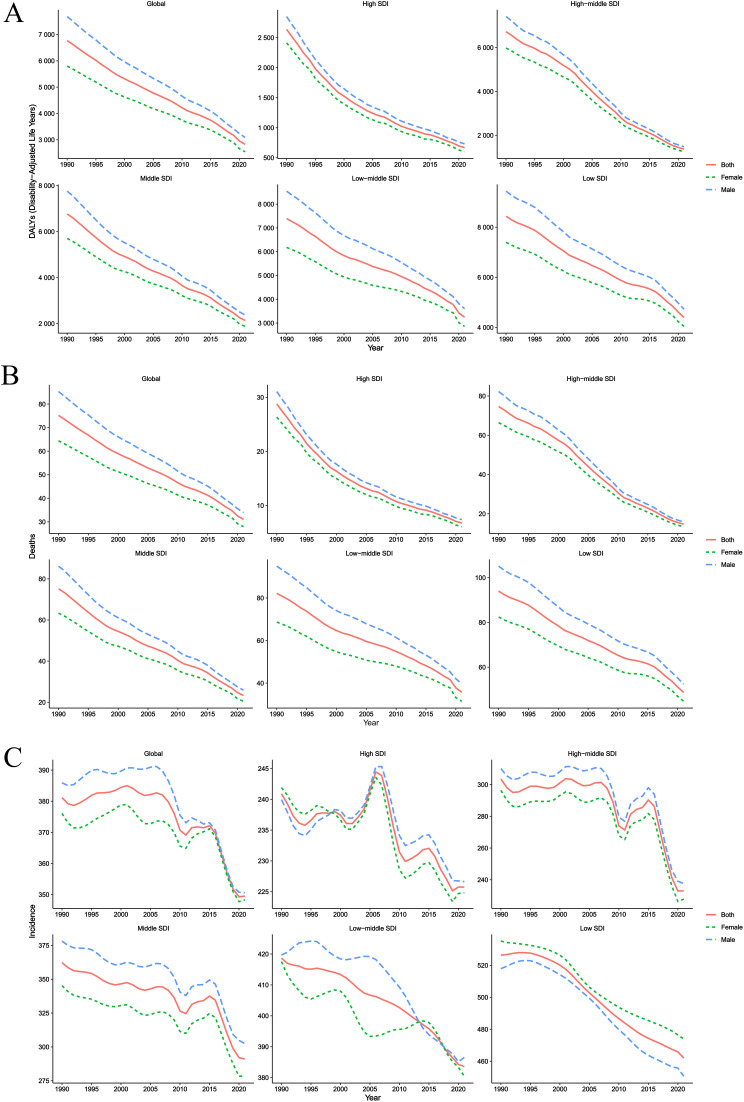
Temporal trends in DALYs, mortality, and incidence rates at global and SDI quintile levels, 1990–2021. The solid red line represents the overall trend (Both), the dashed green line represents females (Female), and the dashed blue line represents males (Male). Rates are per 100,000 person-years. DALYs = Disability-Adjusted Life Years; SDI = Socio-Demographic Index.

The temporal trend maps illustrate the evolution of CHA burden indicators globally (Fig 1). Fig 1A depicts the temporal changes in DALYs rates for CHA at global and different SDI levels. Over the study period (1990–2021), DALYs rates for males (blue curve), females (green curve), and the overall population (red curve) showed a consistent decline globally and across all SDI levels, indicating a reduction in the disease burden attributable to CHA. The decline was more pronounced in high-SDI regions, while low-SDI regions experienced a slower reduction. Although the trends were similar across sexes, males exhibited slightly higher DALYs rates during certain periods. This finding may be related to gender-based healthcare disparities and inherent sex-specific physiological differences. Fig 1B presents the temporal trends in mortality rates. Similar to DALYs rates, mortality rates globally and across SDI levels gradually decreased over the study period. High-SDI regions, benefiting from advanced medical technologies, robust healthcare systems, and heightened health awareness, experienced a faster decline, with mortality rates stabilizing at lower levels in later years. In contrast, low-SDI regions, despite showing a decline, maintained relatively higher mortality rates, which coincided with limited healthcare resources and suboptimal public health conditions. Fig 1C illustrates the temporal trends in incidence rates, which exhibited a more complex pattern. In the early years, incidence rates in some SDI levels fluctuated without a clear monotonic trend, likely due to changes in diagnostic criteria, population mobility, and variations in CHA surveillance methods. However, in later years, most SDI levels began to show a decline in incidence rates, particularly in low-SDI regions, where the reduction was significant. This improvement can likely be attributed to the continuous enhancement and implementation of global public health policies, such as strengthened prenatal care promotion, increased screening efforts, and improved primary healthcare services enabling early diagnosis and intervention. Additionally, variations in the magnitude and timing of declines in incidence rates between sexes suggest that sex-specific biological factors also shape the temporal dynamics of CHA incidence.

### Regional and sex differences

**Fig 2:**
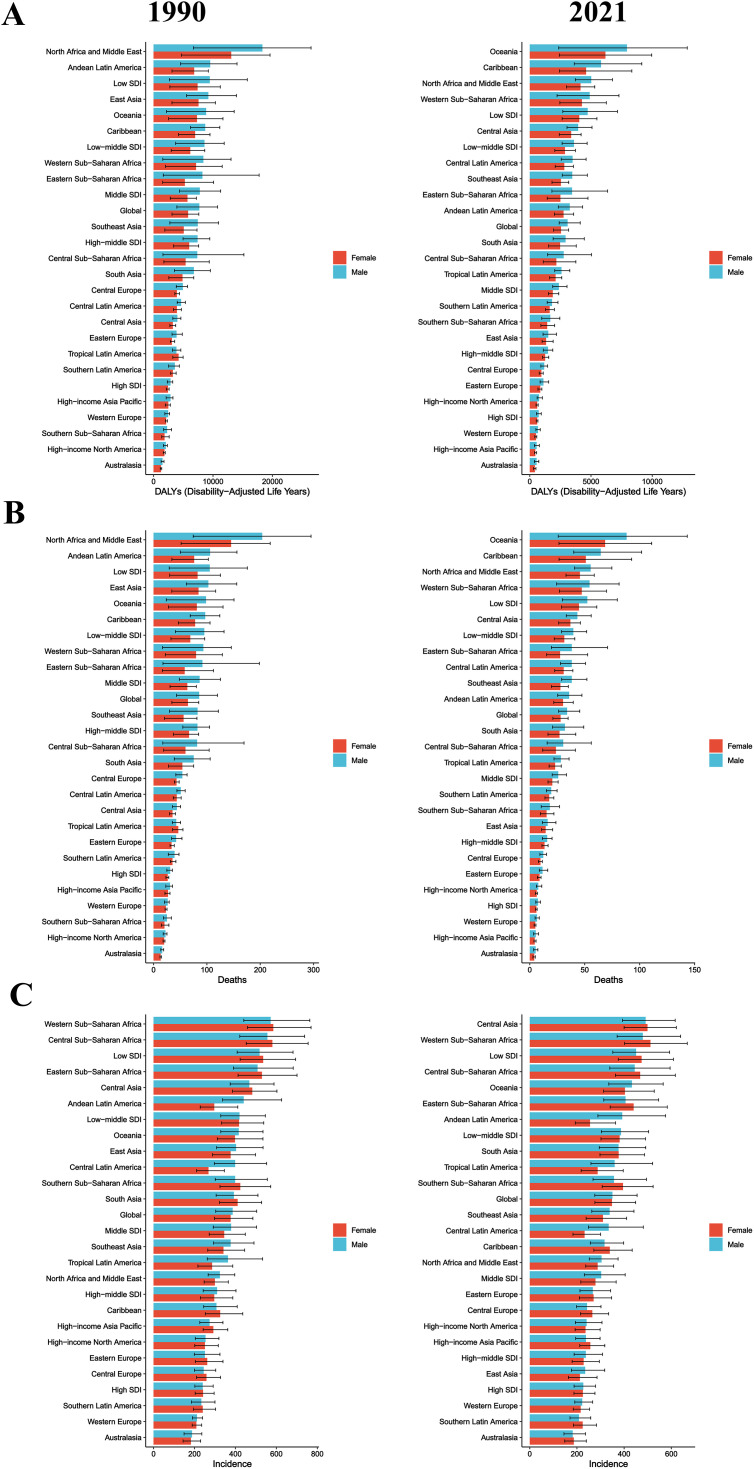
Regional and sex differences in congenital heart anomalies in children under five, 1990 and 2021. **(A)** DALYs rate per 100,000 person-years. **(B)** Absolute number of deaths. **(C)** Incidence rate per 100,000 person-years. Each regional group is divided into two columns: the left column represents 1990, and the right column represents 2021. Bar graphs are color-coded to distinguish females (red) and males (blue). Error bars indicate 95% uncertainty intervals. DALYs = Disability-Adjusted Life Years.

Based on a comparison of data from 1990 and 2021, the regional distribution of DALYs, deaths, and incidence due to CHA has undergone significant changes, with notable increases in Oceania and the Caribbean. Across most regions, the burden point estimates among males were higher than among females (Fig 2). Fig 2A illustrates the sex differences in DALYs due to CHA among children under 5 across different global regions from 1990 to 2021. In 1990, North Africa and the Middle East recorded the highest DALYs, with higher rates observed in males compared to females, while other regions had relatively lower DALYs. By 2021, DALYs in Oceania and the Caribbean had risen sharply, with males in Oceania showing higher DALYs than females, highlighting observable sex differences. Additionally, North Africa and the Middle East, as well as Sub-Saharan Africa, maintained high DALYs levels, with rates in males remaining higher than in females. Fig 2B depicts changes in the number of deaths due to CHA. In 1990, North Africa and the Middle East had the highest number of deaths, with males outnumbering females, followed by the Andean Latin America region. By 2021, Oceania had the highest number of deaths, with males again showing higher numbers than females, while the Caribbean also saw a notable increase in deaths. Furthermore, North Africa and the Middle East, as well as Sub-Saharan Africa, continued to report high death counts, with males outnumbering females. Fig 2C reflects changes in the incidence of CHA. In 1990, Sub-Saharan Africa, Central Asia, and low-SDI regions had the highest incidence rates, with minimal sex differences. By 2021, Central Asia had the highest incidence rates, with higher rates in males compared to females, while Sub-Saharan Africa maintained high incidence rates with no significant sex differences. Additionally, Oceania and the Caribbean experienced marked increases in incidence rates, with males showing higher rates than females.

### The association between CHA burden and SDI

**Fig 3:**
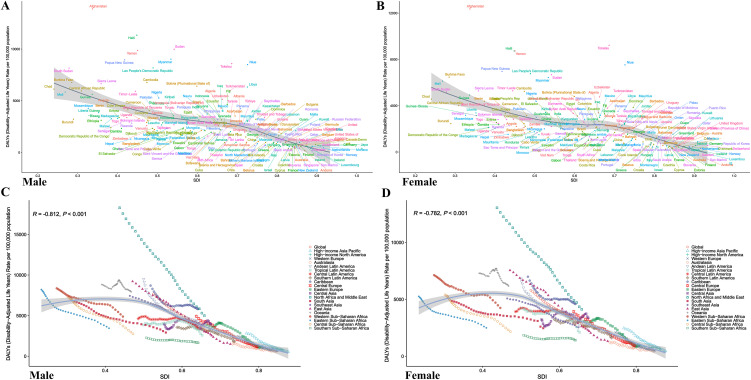
Association between DALYs and SDI for CHA among children under 5 across countries and regions globally. Panels A and C represent males, while Panels B and D represent females. The x-axis represents SDI, and the y-axis represents DALYs rate per 100,000 person-years. Different colors represent different countries and regions, with the gray shaded area indicating the 95% confidence interval of the fitted curve. CHA = Congenital heart anomalies; DALYs = Disability-Adjusted Life Years; SDI = Socio-Demographic Index.

**Fig 4:**
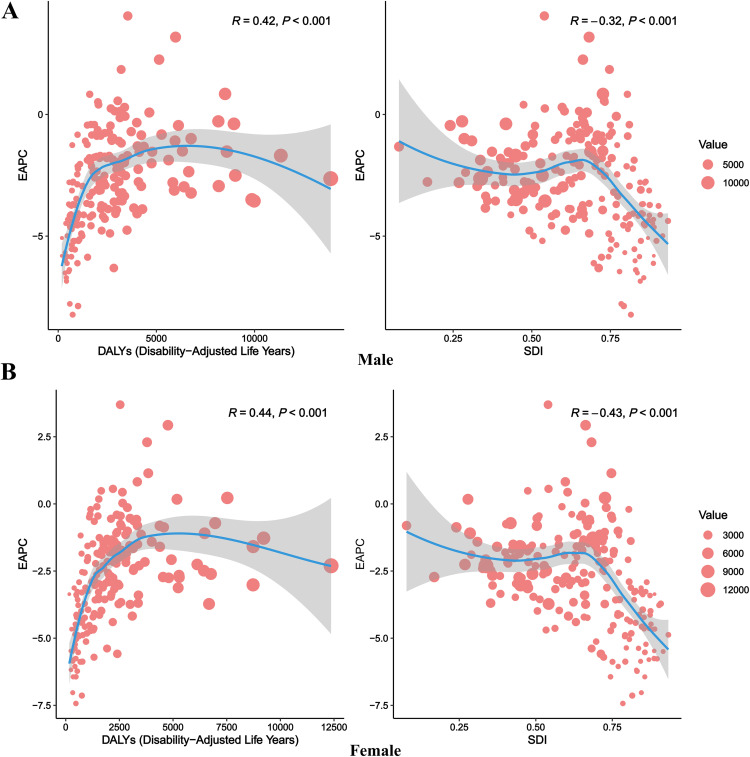
Association between EAPC and DALYs/SDI for CHA among children under 5 globally. Panel A represents males, and Panel B represents females. The left plot shows the relationship between EAPC and DALYs rate (1990); the right plot shows the relationship between EAPC and SDI (2021). Red dots represent different countries. The gray shaded area indicates the 95% confidence interval of the fitted curve. CHA = Congenital heart anomalies; DALYs = Disability-Adjusted Life Years; EAPC = Estimated Annual Percentage Change; SDI = Socio-Demographic Index.

The relationship between CHA burden and SDI was analyzed across 204 countries (Fig 3). Both males and females exhibited a consistent trend: as SDI increased, DALYs decreased. Significant variations were observed among countries, with Afghanistan (low-SDI group) showing notably higher DALYs in Fig 3A,B, while countries like Norway and Japan (high-SDI group) demonstrated significantly lower DALYs, with consistent trends across sexes. In regional subgroup analyses, all regions displayed a similar trend of decreasing DALYs with increasing SDI. High-income regions, such as high-income North America and Australia, exhibited lower DALYs, whereas low-SDI regions like Sub-Saharan Africa reported higher DALYs. Among males, the correlation coefficient between SDI and DALYs was R = −0.812 (P < 0.001, Fig 3C); among females, it was R = −0.782 (P < 0.001, Fig 3D). These results confirm a significant negative correlation between SDI and DALYs, indicating that higher societal development levels are associated with reduced disease burden. In the analysis of EAPC associations (Fig 4), male EAPC showed a positive correlation with DALYs (R = 0.42, P < 0.001) and a negative correlation with SDI (R = −0.32, P < 0.001) (Fig 4A). For females, EAPC exhibited a positive correlation with DALYs (R = 0.44, P < 0.001) and a negative correlation with SDI (R = −0.43, P < 0.001) (Fig 4B). Scatter plots revealed that countries or regions with higher DALYs tended to have positive EAPC growth, while those with higher SDI showed declining EAPC trends, further underscoring the positive impact of societal development on disease burden control.

### Multivariable analysis of sex disparities

After adjusting for SDI, year, and regional random effects, male sex remained an independent risk factor for higher CHA burden (see S10 Table). The adjusted coefficient for males regarding DALYs was 876.4 (95% CI: 793.9 to 958.9, P < 0.001) compared to females. Similarly, for mortality, the adjusted coefficient for males was 9.70 (95% CI: 8.77 to 10.62, P < 0.001). These results indicate that even when holding macro-level development and health-system context (as proxied by SDI) constant, males experience a significantly heavier disease burden.

### Health inequality analysis

**Fig 5:**
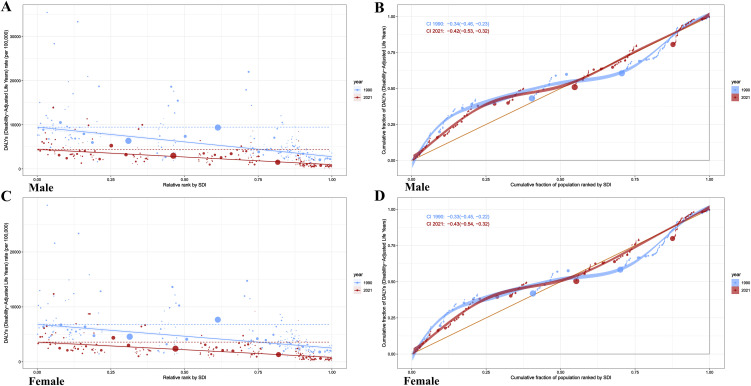
Distribution and cumulative proportion of CHA disease burden by SDI in 1990 and 2021. Panels A (Males) and C (Females) display the distribution of DALYs rate (per 100,000 person-years) relative to the SDI ranking. Panels B (Males) and D (Females) show the concentration curves (cumulative proportion of DALYs vs. cumulative proportion of population). Blue and red colors represent data from 1990 and 2021, respectively. CHA = Congenital heart anomalies; DALYs = Disability-Adjusted Life Years; SDI = Socio-Demographic Index.

The distribution of DALYs rates for CHA across different SDI levels shifted relative to SDI rankings from 1990 to 2021 (Fig 5). As shown in Fig 5A and C, the distribution of DALYs rates in 1990 (blue scatter points) was relatively dispersed, with some high-SDI regions also exhibiting high DALYs rates. By 2021, the red scatter points became more concentrated and shifted toward lower DALYs rates, indicating a reduction in disparities in disease burden across SDI levels and an improvement in inequality. Additionally, sex differences were evident, with males showing higher DALYs rates than females in certain SDI ranking intervals. Fig 5B and D illustrate the relationship between cumulative population proportions and cumulative DALYs proportions. Compared to the 1990 blue curve, the 2021 red curve moved closer to the line of absolute equality (orange line), suggesting a more equitable distribution of disease burden over time. The narrower confidence intervals (CIs) in 2021 further indicate improved data stability and precision. From 1990 to 2021, the concentration index values decreased across the overall population, males, and females (Table 1). For the overall population, the index dropped from −5547.74 (95% CI: −6342.78, −4752.70) to −3131.06 (95% CI: −3562.50, −2699.62); for males, it decreased from −6692.32 (95% CI: −7585.29, −5799.35) to −3458.64 (95% CI: −3921.69, −2995.59); and for females, it declined from −4298.67 (95% CI: −5021.65, −3575.69) to −2769.56 (95% CI: −3174.10, −2365.01). These reductions indicate a decrease in inequality in disease burden over time. Regression curve analysis revealed that the slope for males in 1990 was −0.34 (95% CI: −0.46 to −0.23) and for females was −0.33 (95% CI: −0.45 to −0.22). By 2021, the slope for males decreased to −0.42 (95% CI: −0.53 to −0.32) and for females to −0.43 (95% CI: −0.54 to −0.32), suggesting an increased concentration of disease burden in low-SDI regions over time, with persistent sex differences.

**Table 1:** Health inequality analysis for congenital heart anomalies among children under 5 in 1990 and 2021.

Health inequality analysis		1990	2021
concentration curves	both	−5547.74(−6342.78, −4752.70)	−3131.06(−3562.50, −2699.62)
	male	−6692.32(−7585.29, −5799.35)	−3458.64(−3921.69, −2995.59)
	female	−4298.67(−5021.65, −3575.69)	−2769.56(−3174.10, −2365.01)
regression curves	both	−0.34(−0.45, −0.23)	−0.43(−0.53, −0.32)
	male	−0.34(−0.46, −0.23)	−0.42(−0.53, −0.32)
	female	−0.33(−0.45, −0.22)	−0.43(−0.54, −0.32)

## Discussion

To our knowledge, this study provides a comprehensive assessment of the global, regional, and national burden of congenital heart anomalies (CHA) in children under five and further evaluates whether observed sex differences persist after accounting for macro-level development and temporal change. In the descriptive analyses, males consistently showed higher point estimates for DALYs and mortality than females in 2021, which is broadly consistent with previous GBD-based work [4] and with recent surveillance data from China showing higher mortality in boys [24]. However, the male-to-female uncertainty intervals in the global cross-sectional comparison overlapped 1.0, indicating that crude sex contrasts alone were insufficient to determine whether the observed male disadvantage reflected a stable underlying association or simply heterogeneity across countries and regions.

The additional mixed-effects analysis substantially strengthens the interpretation of our findings. After adjustment for SDI, calendar year, and regional clustering, male sex remained significantly associated with a higher DALY rate and mortality rate. This suggests that the excess burden observed in males is not fully explained by differences in development level or broad secular improvements in diagnosis and care. In this respect, our study extends prior descriptive GBD-style reports [4,25] by showing that the male disadvantage remains detectable even after macro-level contextual factors are taken into account.

Nevertheless, this result should be interpreted within the limits of an ecological model. Our adjusted analysis does not identify an individual-level biological mechanism, nor does it isolate the effect of any specific policy reform. Rather, it indicates that sex disparities in CHA burden persist at the population level after accounting for SDI-related context and time-related change. Taken together, these findings support the view that sex differences in CHA likely reflect a combination of biological susceptibility, variation in lesion severity, and unequal pathways of recognition and treatment across settings.

Our study also demonstrated a strong inverse association between SDI and CHA burden. Countries and regions with lower SDI not only had markedly higher DALY rates, but also experienced slower declines over time, indicating that improvements in CHA outcomes have been unevenly distributed. Importantly, the mixed-effects analysis showed that the male excess burden persisted even after SDI and calendar year were taken into account. This finding suggests that development level is a major determinant of overall burden, but does not fully account for the observed sex disparity.

Geographically, the highest CHA burdens were observed in Sub-Saharan Africa, Southeast Asia, and South Asia, which is consistent with major constraints in prenatal detection, neonatal intensive care, pediatric cardiac surgery, and follow-up care in resource-limited settings. Our findings for North Africa and the Middle East are also in line with the recent GBD-based regional analysis by Soleimani et al.[25], which reported a sustained burden and a slight male predominance. The disproportionate burden in Sub-Saharan Africa and South Asia may reflect the combined effects of limited healthcare resources, malnutrition, infectious exposures, and delayed access to diagnosis and treatment. Conversely, high-SDI regions such as North America, Europe, and Australia maintained substantially lower burden levels, likely reflecting earlier diagnosis, stronger referral systems, and better access to definitive treatment. Taken together, these findings indicate that although social development substantially shapes the overall magnitude of CHA burden, the sex gap remains detectable rather than disappearing once socioeconomic context is considered.

An additional finding requiring cautious interpretation is the apparent increase in burden in Oceania and the Caribbean over time. Because GBD estimates are influenced by changes in data availability, case ascertainment, coding practice, and model inputs, these increases should not automatically be interpreted as a true worsening in underlying disease occurrence. More plausibly, they may reflect a combination of demographic change, improved survival of affected infants, greater diagnostic capture, and evolving reporting systems [26,27]. The more marked male increase observed in some settings may therefore represent either a real difference in severe phenotype distribution or improved identification of cases that were previously missed. Given the ecological nature of the data, these explanations remain hypotheses rather than confirmed mechanisms.

Our health inequality analysis further showed that although the overall distribution of CHA burden became more equitable from 1990 to 2021, the burden remained disproportionately concentrated in low-SDI regions. This pattern suggests that some global progress has been made, but that resource allocation, specialized care capacity, and timely access to surgery remain highly uneven. From a policy perspective, narrowing inequality in average terms should not obscure the continued concentration of avoidable burden in disadvantaged settings.

The persistence of the sex effect after adjustment has important policy implications. If the observed difference had disappeared after accounting for SDI and calendar year, one might reasonably conclude that sex disparities were mainly secondary to uneven development. However, because the disparity remained statistically detectable, our findings suggest that closing development gaps alone may not be sufficient to eliminate sex-based inequities in CHA outcomes. Health-system strengthening remains essential, but it should be accompanied by sex-responsive implementation strategies.

For male infants, this may mean prioritizing rapid identification and referral pathways for critical lesions that present early and require urgent intervention. This interpretation is compatible with clinical experience showing that severe or duct-dependent lesions often demand immediate neonatal management, and with prior estimates that a substantial proportion of preventable CHA-related deaths may be averted through timely surgical access [5]. At the same time, optimizing CHA outcomes cannot be achieved solely through a focus on early male survival. Females may face a different set of risks, namely delayed diagnosis, deferred referral, and reduced access to definitive treatment owing to sociocultural bias. In India, where CHA prevalence shows minimal sex difference, girls have been shown to be underrepresented among pediatric cardiac surgery recipients [28], and referral cohorts have demonstrated lower rates of corrective surgery in girls than in boys [29,30]. Female sex has also been identified as a predictor of surgical non-intervention in this context, potentially reflecting the influence of son preference and unequal household resource allocation [31]. Together, these findings support a dual strategy: strengthening clinical infrastructure for early detection and treatment of critical defects while also implementing gender-sensitive policies to reduce the undertreatment of girls.

Recognizing sex differences in CHA can inform the implementation of screening programs worldwide. Newborn screening for critical CHA—such as pulse oximetry—is now standard in many high-income countries and is being recommended for global adoption [32,33]. While beneficial for all infants, universal newborn screening in low- and middle-income countries could preferentially reduce male mortality from undiagnosed critical CHA, given their slight majority in neonatal cases. Conversely, females with less clinically overt CHA may be more likely to evade neonatal screening and present later. Therefore, health systems should complement screening with robust pediatric surveillance—such as routine checkups in infancy and early childhood—to detect milder lesions more common in girls. In this context, our adjusted findings are relevant because they suggest that sex differences persist beyond broad development-related differences alone. Accordingly, screening policies should not assume that general improvements in health systems will automatically eliminate sex disparities; instead, they should be designed to ensure timely capture of both critically ill male infants and female children with less dramatic early presentations.

At the policy level, the recent 77th World Health Assembly (2024) resolution on improving newborn screening, diagnosis, and management of birth defects provides important international momentum for action [34]. Our findings suggest that implementation of such policies should go beyond expanding screening coverage alone and should also strengthen referral networks, pediatric cardiac workforce capacity, and equitable access to surgery. Gender-sensitive approaches remain especially important in settings where sociocultural barriers may delay care for girls [35]. Ultimately, reducing the global burden of CHA will require both broader health-system investment and explicit attention to the ways in which sex-related biological and social factors shape access to care and outcomes.

Because the sex disparity remained detectable after adjustment for macro-level development and time, biological explanations remain plausible and merit discussion, although our ecological analysis cannot test them directly. Sex differences in the prevalence and severity of CHA arise from a complex interplay of genetic, epigenetic, and hormonal factors. Recent research has shed new light on why males are more prone to severe CHA, while females tend to present with milder forms. The fundamental genetic difference between having two X chromosomes (XX) versus one X and one Y (XY) influences cardiac development from the earliest stages [36]. Unlike many sex differences that manifest after puberty, biases in CHA emerge before gonadal development or sex hormone influence, which suggests a possible role for chromosomal effects. Genes on the X and Y chromosomes—many of which regulate embryonic development—are expressed in cardiac progenitor cells and developing heart structures. An illustrative case is Turner syndrome (45,X in females), where the loss of one X chromosome is associated with a high incidence of CHA, particularly coarctation of the aorta and bicuspid aortic valve, highlighting the role of X-linked gene dosage in heart development [37,38]. Sex-differential expression patterns in embryonic heart tissue likely set the stage for divergent outcomes long before birth. In males, pro-growth pathways or pro-apoptotic pathways might be dialed up or down differently than in females, leading to higher susceptibility to structural defects [36].

While sex hormones are absent in early embryogenesis, hormonal influences later in pregnancy might still contribute to cardiac anomaly risk. One intriguing hypothesis suggests that the maternal endocrine environment during critical periods of heart development could influence the sex ratio of specific defects. James proposed that maternal androgen excess might underlie the male preponderance of transposition of the great arteries [39]. Additionally, male and female fetuses respond differently to intrauterine stresses such as hypoxia or nutrient deficiency, potentially affecting heart formation [40,41]. There is some evidence that male fetuses are more vulnerable to prenatal environmental insults (e.g., maternal smoking, poor placental perfusion), which may contribute to higher rates of malformations in males. Conversely, females may have developmental advantages—for instance, estrogen from the mother or fetus might exert protective effects on developing cardiac tissues [42–44].

### Limitations

This study has several limitations. First, the GBD 2021 estimates are derived from 204 countries and territories, and cross-national differences in data collection, reporting completeness, and diagnostic practices may introduce measurement inconsistency. In low-SDI settings, limited diagnostic capacity and under-reporting may lead to under- or over-estimation of the CHA burden. Second, the GBD 2021 database does not provide severity-stratified estimates, limiting comparisons of burden by disease severity across countries. Third, because this is an ecological analysis, the observed patterns should be interpreted as descriptive associations rather than causal effects. Finally, our EAPC approach assumes a steady log-linear trend and may not capture short-term non-linear fluctuations, and we could not incorporate harmonized country-level covariates reflecting specific policy changes or infrastructure investments; therefore, residual confounding from unmeasured country-specific policy changes, health-system reforms, or service-capacity differences may remain.

## Conclusion

This study provides a global assessment of the burden of congenital heart anomalies in children under five from 1990 to 2021 and shows that substantial sex and regional disparities persist. Although the overall burden declined over time, high-burden regions remained concentrated in low-SDI settings. Importantly, the excess burden observed in males remained significant after accounting for SDI, calendar year, and regional clustering, suggesting that sex disparities are not fully explained by macro-level development alone. These findings support the need for both broader health-system strengthening and sex-responsive strategies in screening, referral, and access to definitive care.

## Supporting information

S1 Table List of International Classification of Diseases Codes for Congenital Heart Anomalies. (DOCX)

S2 Table GATHER Checklist. (DOCX)

S3 Table Geographic regions, countries and territories in GBD 2021. (DOCX)

S4 Table Global and regional trends in disability-adjusted life years due to congenital heart anomalies among children under 5, 2021, and estimated annual percentage change, 1990–2021. (DOCX)

S5 Table Global and regional trends in deaths due to congenital heart anomalies among children under 5, 2021, and estimated annual percentage change, 1990–2021. (DOCX)

S6 Table Global and regional trends in incidence of congenital heart anomalies among children under 5, 2021, and estimated annual percentage change, 1990–2021. (DOCX)

S7 Table National Trends in Disability-Adjusted Life Years Due to Congenital Heart Anomalies among Children Under 5 Years, 2021, and Estimated Annual Percentage Change, 1990–2021. (DOCX)

S8 Table National Trends in Deaths Due to Congenital Heart Anomalies among Children Under 5 Years, 2021, and Estimated Annual Percentage Change, 1990–2021. (DOCX)

S9 Table National Trends in Incidence of Congenital Heart Anomalies among Children Under 5 Years, 2021, and Estimated Annual Percentage Change, 1990–2021. (DOCX)

S10 Table Results of multivariable linear mixed-effects models examining the association between sex and CHA burden, adjusting for SDI and temporal trends. (DOCX)
