# Supplementary material for: Sex differences in global burden of Congenital Heart Anomalies in children under five from 1990 to 2021
Source: PLoS One. 2026 May 6;21(5):e0348351. doi: 10.1371/journal.pone.0348351 (PMC13148693; doi:10.1371/journal.pone.0348351)
Supplement: S1 Table — (DOCX) [file pone.0348351.s001.docx]

**Supplementary Table 1** List of International Classification of Diseases Codes for Congenital Heart Anomalies

| **ICD-10** | **Diagnoses** |
| --- | --- |
| **Q20** | **Congenital malformations of cardiac chambers and connections** |
| Q20.0 | Common arterial trunk |
| Q20.1 | Double outlet right ventricle |
| Q20.2 | Double outlet left ventricle |
| Q20.3 | Discordant ventriculoarterial connection |
| Q20.4 | Double inlet ventricle |
| Q20.5 | Discordant atrioventricular connection |
| Q20.6 | Isomerism of atrial appendages |
| Q20.8 | Other congenital malformations of cardiac chambers and connections |
| Q20.9 | Congenital malformation of cardiac chambers and connections, unspecified |
| **Q21** | **Congenital malformations of cardiac septa** |
| Q21.0 | Ventricular septal defect |
| Q21.1 | Atrial septal defect |
| Q21.2 | Atrioventricular septal defect |
| Q21.3 | Tetralogy of Fallot |
| Q21.4 | Aortopulmonary septal defect |
| Q21.8 | Other congenital malformations of cardiac septa |
| Q21.9 | Congenital malformation of cardiac septum, unspecified |
| **Q22** | **Congenital malformations of pulmonary and tricuspid valves** |
| Q22.0 | Pulmonary valve atresia |
| Q22.1 | Congenital pulmonary valve stenosis |
| Q22.2 | Congenital pulmonary valve insufficiency |
| Q22.3 | Other congenital malformations of pulmonary valve |
| Q22.4 | Congenital tricuspid stenosis |
| Q22.5 | Ebstein anomaly |
| Q22.6 | Hypoplastic right heart syndrome |
| Q22.8 | Other congenital malformations of tricuspid valve |
| Q22.9 | Congenital malformation of tricuspid valve, unspecified |
| **Q23** | **Congenital malformations of aortic and mitral valves** |
| Q23.0 | Congenital stenosis of aortic valve |
| Q23.1 | Congenital insufficiency of aortic valve |
| Q23.2 | Congenital mitral stenosis |
| Q23.3 | Congenital mitral insufficiency |
| Q23.4 | Hypoplastic left heart syndrome |
| Q23.8 | Other congenital malformations of aortic and mitral valves |
| Q23.9 | Congenital malformation of aortic and mitral valves, unspecified |
| **Q24** | **Other congenital malformations of heart** |
| Q24.0 | Dextrocardia |
| Q24.1 | Laevocardia |
| Q24.2 | Cor triatriatum |
| Q24.3 | Pulmonary infundibular stenosis |
| Q24.4 | Congenital subaortic stenosis |
| Q24.5 | Malformation of coronary vessels |
| Q24.6 | Congenital heart block |
| Q24.8 | Other specified congenital malformations of heart |
| Q24.9 | Congenital malformation of heart, unspecified |
| **Q25** | **Congenital malformations of great arteries** |
| Q25.0 | Patent ductus arteriosus |
| Q25.1 | Coarctation of aorta |
| Q25.2 | Atresia of aorta |
| Q25.3 | Stenosis of aorta |
| Q25.4 | Other congenital malformations of aorta |
| Q25.5 | Atresia of pulmonary artery |
| Q25.6 | Stenosis of pulmonary artery |
| Q25.7 | Other congenital malformations of pulmonary artery |
| Q25.8 | Other congenital malformations of great arteries |
| Q25.9 | Congenital malformation of great arteries, unspecified |
| **Q26** | **Congenital malformations of great veins** |
| Q26.0 | Congenital stenosis of vena cava |
| Q26.1 | Persistent left superior vena cava |
| Q26.2 | Total anomalous pulmonary venous connection |
| Q26.3 | Partial anomalous pulmonary venous connection |
| Q26.4 | Anomalous pulmonary venous connection, unspecified |
| Q26.5 | Anomalous portal venous connection |
| Q26.6 | Portal vein-hepatic artery fistula |
| Q26.8 | Other congenital malformations of great veins |
| Q26.9 | Congenital malformation of great vein, unspecified |
| **Q27** | **Other congenital malformations of peripheral vascular system** |
| Q27.0 | Congenital absence and hypoplasia of umbilical artery |
| Q27.1 | Congenital renal artery stenosis |
| Q27.2 | Other congenital malformations of renal artery |
| Q27.3 | Peripheral arteriovenous malformation |
| Q27.4 | Congenital phlebectasia |
| Q27.8 | Other specified congenital malformations of peripheral vascular system |
| Q27.9 | Congenital malformation of peripheral vascular system, unspecified |
| **Q28** | **Other congenital malformations of circulatory system** |
| Q28.0 | Arteriovenous malformation of precerebral vessels |
| Q28.1 | Other malformations of precerebral vessels |
| Q28.2 | Arteriovenous malformation of cerebral vessels |
| Q28.3 | Arteriovenous malformation of cerebral vessels |
| Q28.8 | Other specified congenital malformations of circulatory system |
| Q28.9 | Congenital malformation of circulatory system, unspecified |
