# Supplementary material for: Sex differences in global burden of Congenital Heart Anomalies in children under five from 1990 to 2021
Source: PLoS One. 2026 May 6;21(5):e0348351. doi: 10.1371/journal.pone.0348351 (PMC13148693; doi:10.1371/journal.pone.0348351)
Supplement: S2 Table — (DOCX) [file pone.0348351.s002.docx]

**Supplementary Table 2** GATHER Checklist

| GATHER Item | Description | Location in Manuscript |
| --- | --- | --- |
| 1 | Define the health outcome or parameter estimated. | Abstract, Introduction, Methods (Data sources) |
| 2 | State the study's objectives. | Abstract, Introduction (Last paragraph) |
| 3 | Describe the data sources used for the estimates. | Methods (Data sources) |
| 4 | Provide the time period and geographical location. | Abstract, Methods, Title |
| 5 | For GBD studies, cite the GBD capstone papers. | Methods, References [9, 10, 11] |
| 6 | Describe the statistical methods in detail. | Methods (SDI, DALYs, EAPC, Statistical Analysis) |
| 7 | Describe methods for quantifying uncertainty. | Methods (DALYs, EAPC) |
| 8 | Provide estimates of the health outcome. | Results, Table 1, S4-S9 |
| 9 | Report uncertainty intervals. | Results, Table 1, S4-S9 |
| 10 | Present estimates by age, sex, and location. | Results, Figures 1-5, Table 1, S4-S9 |
| 11 | Present estimates for all time points. | Results, Figures 1-5, Table 1, S4-S9 |
| 12 | State which data and code are publicly available. | Submission system Data Availability Statement; figshare repository |
| 13 | Provide access to the data and code. | Submission system Data Availability Statement; figshare repository |
| 14 | Provide clear and precise figure/table legends. | Figure 1-5 Legends, Table 1 Legend, S1-S10 captions |
| 15 | Describe all known limitations of the estimates. | Discussion (Limitations section) |
| 16 | Describe sources of non-sampling error. | Discussion (Limitations section) |
| 17 | Compare estimates with other studies. | Discussion (Paragraphs 1-3) |
| 18 | Discuss the implications of the findings. | Discussion, Conclusion |
| 19 | Provide information on funding sources. | Acknowledgments |
