# Supplementary material for: Sex differences in global burden of Congenital Heart Anomalies in children under five from 1990 to 2021
Source: PLoS One. 2026 May 6;21(5):e0348351. doi: 10.1371/journal.pone.0348351 (PMC13148693; doi:10.1371/journal.pone.0348351)
Supplement: S3 Table — (DOCX) [file pone.0348351.s003.docx]

**Supplementary Table 3** Geographic regions, countries and territories in GBD 2021.

| **Geographic Regions** | **Countries or territories** |
| --- | --- |
| **Global** |  |
| **Central Europe, eastern Europe, and central Asia** |  |
| **Central Asia** |  |
|  | Armenia |
|  | Azerbaijan |
|  | Georgia |
|  | Kazakhstan |
|  | Kyrgyzstan |
|  | Mongolia |
|  | Tajikistan |
|  | Turkmenistan |
|  | Uzbekistan |
| **Central Europe** |  |
|  | Albania |
|  | Bosnia and Herzegovina |
|  | Bulgaria |
|  | Croatia |
|  | Czechia |
|  | Hungary |
|  | North Macedonia |
|  | Montenegro |
|  | Poland |
|  | Romania |
|  | Serbia |
|  | Slovakia |
|  | Slovenia |
| **Eastern Europe** |  |
|  | Belarus |
|  | Estonia |
|  | Latvia |
|  | Lithuania |
|  | Moldova |
|  | Russia |
|  | Ukraine |
| **High income** |  |
| **Australasia** |  |
|  | Australia |
|  | New Zealand |
| **High-income Asia Pacific** |  |
|  | Brunei |
|  | Japan |
|  | South Korea |
|  | Singapore |
| **High-income North America** |  |
|  | Canada |
|  | Greenland |
|  | USA |
| **Southern Latin America** |  |
|  | Argentina |
|  | Chile |
|  | Uruguay |
| **Western Europe** |  |
|  | Andorra |
|  | Austria |
|  | Belgium |
|  | Cyprus |
|  | Denmark |
|  | Finland |
|  | France |
|  | Germany |
|  | Greece |
|  | Iceland |
|  | Ireland |
|  | Israel |
|  | Italy |
|  | Luxembourg |
|  | Malta |
|  | Monaco |
|  | Netherlands |
|  | Norway |
|  | Portugal |
|  | San Marino |
|  | Spain |
|  | Sweden |
|  | Switzerland |
|  | UK |
| **Latin America and Caribbean** |  |
| **Andean Latin America** |  |
|  | Bolivia |
|  | Ecuador |
|  | Peru |
| **Caribbean** |  |
|  | Antigua and Barbuda |
|  | The Bahamas |
|  | Barbados |
|  | Belize |
|  | Bermuda |
|  | Cuba |
|  | Dominica |
|  | Dominican Republic |
|  | Grenada |
|  | Guyana |
|  | Haiti |
|  | Jamaica |
|  | Puerto Rico |
|  | Saint Kitts and Nevis |
|  | Saint Lucia |
|  | Saint Vincent and the Grenadines |
|  | Suriname |
|  | Trinidad and Tobago |
|  | Virgin Islands |
| **Central Latin America** |  |
|  | Colombia |
|  | Costa Rica |
|  | El Salvador |
|  | Guatemala |
|  | Honduras |
|  | Mexico |
|  | Nicaragua |
|  | Panama |
|  | Venezuela |
| **Tropical Latin America** |  |
|  | Brazil |
|  | Paraguay |
| **North Africa and Middle East** |  |
| **North Africa and Middle East** |  |
|  | Afghanistan |
|  | Algeria |
|  | Bahrain |
|  | Egypt |
|  | Iran |
|  | Iraq |
|  | Jordan |
|  | Kuwait |
|  | Lebanon |
|  | Libya |
|  | Morocco |
|  | Palestine |
|  | Oman |
|  | Qatar |
|  | Saudi Arabia |
|  | Sudan |
|  | Syria |
|  | Tunisia |
|  | Türkiye |
|  | United Arab Emirates |
|  | Yemen |
| **South Asia** |  |
| **South Asia** |  |
|  | Bangladesh |
|  | Bhutan |
|  | India |
|  | Nepal |
|  | Pakistan |
| **Southeast Asia, east Asia, and Oceania** |  |
| **East Asia** |  |
|  | China |
|  | North Korea |
|  | Taiwan (Province of China) |
| **Oceania** |  |
|  | American Samoa |
|  | Cook Islands |
|  | Fiji |
|  | Guam |
|  | Kiribati |
|  | Marshall Islands |
|  | Federated States of Micronesia |
|  | Nauru |
|  | Niue |
|  | Northern Mariana Islands |
|  | Palau |
|  | Papua New Guinea |
|  | Samoa |
|  | Solomon Islands |
|  | Tokelau |
|  | Tonga |
|  | Tuvalu |
|  | Vanuatu |
| **Southeast Asia** |  |
|  | Cambodia |
|  | Indonesia |
|  | Laos |
|  | Malaysia |
|  | Maldives |
|  | Mauritius |
|  | Myanmar |
|  | Philippines |
|  | Seychelles |
|  | Sri Lanka |
|  | Thailand |
|  | Timor-Leste |
|  | Viet Nam |
| **Sub-Saharan Africa** |  |
| **Central Sub-Saharan Africa** |  |
|  | Angola |
|  | Central African Republic |
|  | Congo (Brazzaville) |
|  | DR Congo |
|  | Equatorial Guinea |
|  | Gabon |
| **Eastern Sub-Saharan Africa** |  |
|  | Burundi |
|  | Comoros |
|  | Djibouti |
|  | Eritrea |
|  | Ethiopia |
|  | Kenya |
|  | Madagascar |
|  | Malawi |
|  | Mozambique |
|  | Rwanda |
|  | Somalia |
|  | South Sudan |
|  | Tanzania |
|  | Uganda |
|  | Zambia |
| **Southern Sub-Saharan Africa** |  |
|  | Botswana |
|  | Lesotho |
|  | Namibia |
|  | South Africa |
|  | Eswatini |
|  | Zimbabwe |
| **Western Sub-Saharan Africa** |  |
|  | Benin |
|  | Burkina Faso |
|  | Cameroon |
|  | Cabo Verde |
|  | Chad |
|  | Côte d'Ivoire |
|  | The Gambia |
|  | Ghana |
|  | Guinea |
|  | Guinea-Bissau |
|  | Liberia |
|  | Mali |
|  | Mauritania |
|  | Niger |
|  | Nigeria |
|  | São Tomé and Príncipe |
|  | Senegal |
|  | Sierra Leone |
|  | Togo |
