# Supplementary material for: Sex differences in global burden of Congenital Heart Anomalies in children under five from 1990 to 2021
Source: PLoS One. 2026 May 6;21(5):e0348351. doi: 10.1371/journal.pone.0348351 (PMC13148693; doi:10.1371/journal.pone.0348351)
Supplement: S4 Table — (DOCX) [file pone.0348351.s004.docx]

**Supplementary Table 4.** Global and regional trends in disability-adjusted life years due to congenital heart anomalies among children under 5, 2021, and estimated annual percentage change, 1990–2021.

| Location | Male | | | Female | | | Male-to-Female  Rate Ratio  (95% UI) |
| --- | --- | --- | --- | --- | --- | --- | --- |
|  | 2021 | | EAPC from 1990 to 2021 | 2021 | | EAPC from 1990 to 2021 |  |
|  | DALYs number(95% UI) | DALYs rate(95% UI) | rate(95% CI) | DALYs number(95% UI) | DALYs rate(95% UI) | rate(95% CI) |  |
| Global | 10497778.44(8166300.99,14012907.11) | 3087.77(2402.00,4121.69) | -2.70(-2.81,-2.58) | 8101048.99(6182975.51,10081965.26) | 2545.96(1943.16,3168.51) | -2.28(-2.41,-2.15) | 1.21(0.84–1.75) |
| East Asia | 643742.40(464566.60,933482.09) | 1501.76(1083.77,2177.68) | -5.79(-6.18,-5.39) | 492828.29(375501.57,710064.12) | 1324.53(1009.20,1908.38) | -5.62(-6.03,-5.21) | 1.13(0.71–1.82) |
| Oceania | 80105.32(23880.83,129828.92) | 7934.28(2365.35,12859.31) | -0.16(-0.33,0.01) | 57096.73(22357.25,92091.58) | 6173.68(2417.42,9957.57) | -0.34(-0.52,-0.16) | 1.29(0.43–3.87) |
| Central Europe | 33855.91(26035.44,41626.30) | 1179.40(906.96,1450.08) | -4.61(-4.75,-4.47) | 25556.79(21043.74,29852.79) | 941.30(775.07,1099.53) | -4.62(-4.79,-4.45) | 1.25(0.94–1.68) |
| Eastern Europe | 57863.71(43943.00,80409.25) | 1112.69(845.00,1546.23) | -4.18(-4.93,-3.42) | 42183.37(32909.56,48626.44) | 857.63(669.09,988.63) | -4.42(-5.25,-3.59) | 1.30(0.91–1.86) |
| Australasia | 5173.54(3547.76,6911.37) | 554.06(379.95,740.17) | -2.96(-3.16,-2.75) | 3596.65(2714.50,4454.30) | 407.64(307.66,504.85) | -3.17(-3.35,-2.99) | 1.36(0.90–2.06) |
| High-income Asia Pacific | 18421.64(12670.76,25867.96) | 557.16(383.23,782.38) | -4.99(-5.15,-4.83) | 14169.32(12348.51,17405.05) | 450.42(392.54,553.27) | -5.08(-5.26,-4.90) | 1.24(0.83–1.84) |
| Southeast Asia | 1004475.48(769184.56,1363284.82) | 3465.20(2653.50,4703.00) | -2.42(-2.55,-2.29) | 692734.91(495767.39,868553.17) | 2537.77(1816.20,3181.86) | -1.82(-2.14,-1.49) | 1.37(0.91–2.04) |
| Central Asia | 204617.06(157509.42,263411.77) | 3947.68(3038.83,5082.01) | 0.50(0.17,0.82) | 162423.47(116025.40,201733.54) | 3374.12(2410.27,4190.73) | 0.76(0.35,1.17) | 1.17(0.80–1.71) |
| Western Europe | 72283.71(54523.61,93148.08) | 664.38(501.14,856.15) | -4.21(-4.40,-4.02) | 52109.50(42592.16,59529.66) | 503.50(411.54,575.20) | -4.47(-4.66,-4.28) | 1.32(0.96–1.81) |
| Southern Latin America | 39603.35(31097.55,50309.14) | 1812.57(1423.28,2302.56) | -1.86(-2.20,-1.52) | 34079.60(26916.13,42076.28) | 1627.84(1285.67,2009.81) | -1.63(-1.81,-1.44) | 1.11(0.80–1.55) |
| High-income North America | 79038.49(63052.59,108017.66) | 754.12(601.60,1030.62) | -2.79(-2.98,-2.61) | 61174.33(51365.34,68993.28) | 610.66(512.74,688.71) | -2.63(-2.87,-2.38) | 1.23(0.91–1.68) |
| Caribbean | 114858.43(71687.92,180474.39) | 5821.80(3633.63,9147.66) | -0.93(-1.15,-0.71) | 87100.76(46020.61,157967.56) | 4595.67(2428.17,8334.80) | -1.36(-1.48,-1.25) | 1.27(0.59–2.74) |
| Andean Latin America | 103101.33(73567.19,136254.67) | 3263.84(2328.89,4313.36) | -2.76(-2.94,-2.57) | 82387.10(60422.97,107336.89) | 2749.07(2016.18,3581.59) | -2.35(-2.47,-2.24) | 1.19(0.78–1.81) |
| Central Latin America | 356448.90(262474.09,470077.96) | 3487.74(2568.22,4599.56) | -0.64(-0.86,-0.43) | 277735.38(205295.76,351598.24) | 2813.84(2079.93,3562.17) | -0.48(-0.68,-0.28) | 1.24(0.83–1.84) |
| Tropical Latin America | 227871.97(179822.15,287868.54) | 2587.70(2042.05,3269.02) | -0.75(-1.17,-0.33) | 176907.57(136673.74,219557.01) | 2105.63(1626.75,2613.26) | -0.97(-1.17,-0.77) | 1.23(0.88–1.72) |
| North Africa and Middle East | 1576830.22(1171168.18,2124304.43) | 5011.72(3722.38,6751.78) | -3.86(-4.04,-3.68) | 1226993.46(892379.77,1574785.89) | 4134.88(3007.25,5306.91) | -3.42(-3.61,-3.23) | 1.21(0.80–1.83) |
| South Asia | 2413642.91(1577616.84,3680077.15) | 2919.91(1908.53,4451.98) | -2.44(-2.55,-2.33) | 1873817.73(1169839.40,2883073.60) | 2467.75(1540.64,3796.91) | -2.01(-2.18,-1.85) | 1.18(0.64–2.20) |
| Central Sub-Saharan Africa | 295655.03(156005.88,538629.80) | 2763.92(1458.41,5035.35) | -2.81(-3.11,-2.52) | 224512.46(114173.91,390216.85) | 2165.12(1101.05,3763.11) | -2.96(-3.17,-2.75) | 1.28(0.53–3.06) |
| Southern Sub-Saharan Africa | 67809.49(39825.19,99717.60) | 1669.36(980.43,2454.89) | -0.74(-0.84,-0.63) | 56053.74(36029.32,80297.82) | 1412.96(908.20,2024.09) | -0.72(-0.99,-0.45) | 1.18(0.64–2.17) |
| Eastern Sub-Saharan Africa | 1120576.68(586852.21,2061354.62) | 3451.52(1807.58,6349.24) | -2.53(-2.64,-2.42) | 783086.04(445973.26,1489026.78) | 2499.46(1423.46,4752.69) | -2.24(-2.44,-2.03) | 1.38(0.58–3.30) |
| Western Sub-Saharan Africa | 1981802.86(903169.79,2954323.03) | 4885.03(2226.26,7282.24) | -1.36(-1.50,-1.22) | 1674501.77(967709.96,2462745.91) | 4251.17(2456.79,6252.33) | -1.43(-1.85,-1.00) | 1.15(0.54–2.44) |
| High SDI | 202247.75(153240.67,259839.43) | 732.31(554.86,940.84) | -4.10(-4.25,-3.95) | 157372.37(138228.36,175052.05) | 600.01(527.02,667.41) | -4.15(-4.30,-4.00) | 1.22(0.91–1.63) |
| Low SDI | 3998288.10(2283744.04,6056429.13) | 4725.81(2699.29,7158.45) | -2.02(-2.11,-1.93) | 3271531.84(2139100.69,4439671.01) | 4040.43(2641.84,5483.11) | -1.72(-1.81,-1.63) | 1.17(0.64–2.15) |
| High-middle SDI | 542812.08(405732.21,690900.64) | 1477.97(1104.73,1881.19) | -5.28(-5.61,-4.95) | 417066.43(348115.30,516503.09) | 1251.78(1044.83,1550.22) | -5.12(-5.45,-4.79) | 1.18(0.85–1.64) |
| Middle SDI | 2183782.04(1728140.71,2799178.36) | 2372.93(1877.82,3041.63) | -3.45(-3.61,-3.29) | 1589955.86(1292707.21,2002545.02) | 1879.64(1528.23,2367.40) | -3.18(-3.47,-2.90) | 1.26(0.91–1.75) |
| Low-middle SDI | 3558734.75(2607871.53,4626257.06) | 3604.21(2641.20,4685.38) | -2.45(-2.58,-2.31) | 2656378.78(1898621.86,3459508.06) | 2861.25(2045.05,3726.32) | -2.13(-2.23,-2.03) | 1.26(0.83–1.91) |

DALYs = disability-adjusted life years; EAPC = estimated annual percentage change.
