# Supplementary material for: Sex differences in global burden of Congenital Heart Anomalies in children under five from 1990 to 2021
Source: PLoS One. 2026 May 6;21(5):e0348351. doi: 10.1371/journal.pone.0348351 (PMC13148693; doi:10.1371/journal.pone.0348351)
Supplement: S5 Table — (DOCX) [file pone.0348351.s005.docx]

**Supplementary Table 5.** Global and regional trends in deaths due to congenital heart anomalies among children under 5, 2021, and estimated annual percentage change, 1990–2021.

| Location | Male | | | Female | | | Male-to-Female  Rate Ratio  (95% UI) |
| --- | --- | --- | --- | --- | --- | --- | --- |
|  | 2021 | | EAPC from 1990 to 2021 | 2021 | | EAPC from 1990 to 2021 |  |
|  | death number(95% UI) | mortality rate(95% UI) | rate(95% CI) | death number(95% UI) | mortality rate(95% UI) | rate(95% CI) |  |
| Global | 115325.46(89213.72,154597.96) | 33.92(26.24,45.47) | -2.62(-2.69,-2.55) | 88897.51(66945.96,111022.15) | 27.94(21.04,34.89) | -2.27(-2.35,-2.20) | 1.21(0.84–1.76) |
| East Asia | 6948.41(4914.61,10204.09) | 16.21(11.47,23.80) | -4.75(-5.16,-4.33) | 5331.27(4024.18,7750.84) | 14.33(10.82,20.83) | -4.87(-4.98,-4.76) | 1.13(0.69–1.85) |
| Oceania | 889.81(261.41,1446.77) | 88.13(25.89,143.30) | -0.17(-0.27,-0.07) | 634.23(245.71,1026.03) | 68.58(26.57,110.94) | -0.28(-0.41,-0.14) | 1.29(0.42–3.92) |
| Central Europe | 351.17(264.48,436.14) | 12.23(9.21,15.19) | -4.41(-4.59,-4.24) | 264.07(214.76,310.78) | 9.73(7.91,11.45) | -4.43(-4.52,-4.34) | 1.26(0.92–1.72) |
| Eastern Europe | 603.87(453.76,850.70) | 11.61(8.73,16.36) | -3.26(-3.81,-2.71) | 436.71(343.17,504.71) | 8.88(6.98,10.26) | -3.38(-3.50,-3.26) | 1.31(0.90–1.89) |
| Australasia | 50.25(32.96,68.85) | 5.38(3.53,7.37) | -3.59(-3.76,-3.42) | 35.41(25.37,44.77) | 4.01(2.88,5.07) | -3.49(-3.66,-3.33) | 1.34(0.84–2.13) |
| High-income Asia Pacific | 179.32(115.94,262.12) | 5.42(3.51,7.93) | -5.03(-5.16,-4.91) | 139.05(120.30,179.86) | 4.42(3.82,5.72) | -4.94(-5.11,-4.78) | 1.23(0.78–1.93) |
| Southeast Asia | 11057.12(8417.73,15071.73) | 38.14(29.04,51.99) | -2.15(-2.26,-2.04) | 7605.90(5403.85,9581.96) | 27.86(19.80,35.10) | -1.96(-2.08,-1.83) | 1.37(0.91–2.06) |
| Central Asia | 2241.33(1721.87,2896.56) | 43.24(33.22,55.88) | 0.00(-0.24,0.24) | 1778.15(1265.57,2216.55) | 36.94(26.29,46.05) | 0.26(-0.02,0.54) | 1.17(0.80–1.72) |
| Western Europe | 713.03(525.06,943.71) | 6.55(4.83,8.67) | -4.61(-4.72,-4.50) | 515.37(408.49,586.08) | 4.98(3.95,5.66) | -4.50(-4.94,-4.06) | 1.32(0.93–1.85) |
| Southern Latin America | 426.66(332.25,543.27) | 19.53(15.21,24.86) | -1.38(-1.63,-1.13) | 368.49(289.60,458.69) | 17.60(13.83,21.91) | -1.49(-1.71,-1.26) | 1.11(0.79–1.55) |
| High-income North America | 809.41(635.23,1145.60) | 7.72(6.06,10.93) | -3.25(-3.39,-3.11) | 625.63(515.16,709.63) | 6.25(5.14,7.08) | -2.92(-3.06,-2.78) | 1.24(0.88–1.73) |
| Caribbean | 1273.42(790.10,2008.71) | 64.55(40.05,101.81) | -1.48(-1.68,-1.28) | 965.12(504.94,1757.19) | 50.92(26.64,92.71) | -1.51(-1.76,-1.25) | 1.27(0.58–2.76) |
| Andean Latin America | 1131.21(803.72,1498.99) | 35.81(25.44,47.45) | -3.22(-3.38,-3.05) | 905.28(655.80,1186.00) | 30.21(21.88,39.57) | -2.70(-2.87,-2.53) | 1.19(0.77–1.82) |
| Central Latin America | 3907.36(2858.85,5180.81) | 38.23(27.97,50.69) | -0.42(-0.56,-0.27) | 3044.54(2239.90,3871.61) | 30.85(22.69,39.22) | -0.41(-0.59,-0.23) | 1.24(0.83–1.86) |
| Tropical Latin America | 2487.19(1940.49,3148.36) | 28.24(22.04,35.75) | -0.57(-0.82,-0.31) | 1929.96(1476.42,2409.14) | 22.97(17.57,28.67) | -0.95(-1.15,-0.75) | 1.23(0.87–1.73) |
| North Africa and Middle East | 17389.81(12823.20,23506.47) | 55.27(40.76,74.71) | -3.86(-3.97,-3.74) | 13527.46(9781.33,17412.12) | 45.59(32.96,58.68) | -3.75(-3.96,-3.54) | 1.21(0.80–1.84) |
| South Asia | 26425.83(17160.87,40618.19) | 31.97(20.76,49.14) | -2.57(-2.65,-2.49) | 20517.50(12582.70,31792.94) | 27.02(16.57,41.87) | -2.05(-2.14,-1.95) | 1.18(0.63–2.23) |
| Central Sub-Saharan Africa | 3259.48(1691.23,5989.11) | 30.47(15.81,55.99) | -2.13(-2.39,-1.86) | 2455.72(1224.93,4304.16) | 23.68(11.81,41.51) | -1.80(-1.93,-1.68) | 1.29(0.53–3.14) |
| Southern Sub-Saharan Africa | 738.22(423.57,1096.40) | 18.17(10.43,26.99) | -1.25(-1.41,-1.09) | 603.34(382.39,871.80) | 15.21(9.64,21.98) | -1.23(-1.31,-1.15) | 1.19(0.64–2.24) |
| Eastern Sub-Saharan Africa | 12415.10(6408.23,22969.19) | 38.24(19.74,70.75) | -2.30(-2.40,-2.19) | 8620.61(4810.33,16505.60) | 27.52(15.35,52.68) | -1.97(-2.06,-1.89) | 1.39(0.57–3.37) |
| Western Sub-Saharan Africa | 22027.46(9912.78,32974.55) | 54.30(24.43,81.28) | -1.28(-1.36,-1.19) | 18593.70(10620.44,27498.63) | 47.21(26.96,69.81) | -1.48(-1.67,-1.30) | 1.15(0.53–2.48) |
| High SDI | 2043.14(1521.34,2673.66) | 7.40(5.51,9.68) | -4.41(-4.49,-4.32) | 1595.25(1374.17,1768.59) | 6.08(5.24,6.74) | -4.29(-4.63,-3.95) | 1.22(0.89–1.66) |
| Low SDI | 44344.27(25089.47,67399.15) | 52.41(29.65,79.66) | -1.95(-2.01,-1.89) | 36232.18(23426.60,49392.94) | 44.75(28.93,61.00) | -1.68(-1.73,-1.62) | 1.17(0.63–2.18) |
| High-middle SDI | 5815.47(4215.79,7445.88) | 15.83(11.48,20.27) | -4.50(-4.83,-4.18) | 4469.23(3683.67,5551.76) | 13.41(11.06,16.66) | -4.45(-4.63,-4.27) | 1.18(0.83–1.68) |
| Middle SDI | 23822.99(18771.58,30688.16) | 25.89(20.40,33.35) | -3.29(-3.40,-3.17) | 17309.43(14033.81,21831.75) | 20.46(16.59,25.81) | -3.49(-4.08,-2.90) | 1.27(0.91–1.76) |
| Low-middle SDI | 39168.07(28534.84,51113.43) | 39.67(28.90,51.77) | -2.46(-2.54,-2.37) | 29195.01(20729.03,38039.84) | 31.45(22.33,40.97) | -2.04(-2.27,-1.81) | 1.26(0.83–1.92) |

DALYs = disability-adjusted life years; EAPC = estimated annual percentage change.
