# Supplementary material for: Sex differences in global burden of Congenital Heart Anomalies in children under five from 1990 to 2021
Source: PLoS One. 2026 May 6;21(5):e0348351. doi: 10.1371/journal.pone.0348351 (PMC13148693; doi:10.1371/journal.pone.0348351)
Supplement: S6 Table — (DOCX) [file pone.0348351.s006.docx]

**Supplementary Table 6.** Global and regional trends in incidence of congenital heart anomalies among children under 5, 2021, and estimated annual percentage change, 1990–2021.

| Location | Male | | | Female | | | Male-to-Female  Rate Ratio  (95% UI) |
| --- | --- | --- | --- | --- | --- | --- | --- |
|  | 2021 | | EAPC from  1990 to 2021 | 2021 | | EAPC from  1990 to 2021 |  |
|  | incidence number(95% UI) | incidence rate(95% UI) | rate(95% CI) | incidence number(95% UI) | incidence rate(95% UI) | rate(95% CI) |  |
| Global | 1191984.34(937419.10,1546979.55) | 350.61(275.73,455.02) | -0.30(-0.38,-0.22) | 1108343.03(878454.29,1428152.58) | 348.33(276.08,448.83) | -0.24(-0.31,-0.17) | 1.01(0.71–1.43) |
| East Asia | 100547.54(75794.39,136198.04) | 234.56(176.82,317.73) | -1.23(-1.58,-0.88) | 79087.39(60936.71,106115.27) | 212.56(163.77,285.20) | -1.28(-1.64,-0.93) | 1.10(0.74–1.65) |
| Oceania | 4366.95(3383.30,5711.17) | 432.54(335.11,565.68) | 0.12(0.06,0.19) | 3728.08(2896.35,4880.63) | 403.11(313.17,527.73) | 0.15(0.09,0.21) | 1.07(0.74–1.55) |
| Central Europe | 6968.16(5698.17,8666.73) | 242.74(198.50,301.91) | 0.05(-0.08,0.19) | 7194.73(5846.80,9071.81) | 264.99(215.35,334.13) | 0.09(-0.03,0.20) | 0.92(0.68–1.24) |
| Eastern Europe | 13885.38(11063.66,17806.94) | 267.01(212.75,342.42) | 0.33(-0.06,0.72) | 13293.59(10346.22,17054.39) | 270.27(210.35,346.73) | 0.36(-0.03,0.75) | 0.99(0.70–1.40) |
| Australasia | 1696.33(1353.02,2202.49) | 181.67(144.90,235.88) | 0.17(0.02,0.32) | 1647.01(1308.26,2116.50) | 186.67(148.28,239.88) | 0.18(0.05,0.31) | 0.97(0.69–1.37) |
| High-income Asia Pacific | 7865.78(6431.83,9852.67) | 237.90(194.53,297.99) | -0.61(-0.71,-0.51) | 8075.07(6672.28,10008.03) | 256.69(212.10,318.14) | -0.55(-0.69,-0.42) | 0.93(0.69–1.24) |
| Southeast Asia | 98139.00(76230.37,128041.97) | 338.56(262.98,441.71) | -0.33(-0.38,-0.28) | 84599.93(65200.28,112012.50) | 309.92(238.86,410.35) | -0.32(-0.36,-0.28) | 1.09(0.75–1.59) |
| Central Asia | 25433.48(20385.92,31955.41) | 490.69(393.31,616.52) | 0.48(0.29,0.68) | 24025.01(19253.02,29909.53) | 499.09(399.96,621.33) | 0.39(0.21,0.58) | 0.98(0.72–1.35) |
| Western Europe | 24186.08(20769.69,29015.89) | 222.30(190.90,266.69) | 0.16(0.05,0.28) | 22313.78(19075.19,26227.07) | 215.61(184.31,253.42) | 0.16(0.07,0.24) | 1.03(0.82–1.30) |
| Southern Latin America | 4557.34(3724.33,5647.54) | 208.58(170.46,258.48) | -0.22(-0.37,-0.07) | 4696.70(3878.52,5915.54) | 224.34(185.26,282.56) | -0.22(-0.35,-0.09) | 0.93(0.69–1.25) |
| High-income North America | 25224.80(20287.10,32090.12) | 240.68(193.56,306.18) | -0.12(-0.19,-0.05) | 23618.16(19264.07,29881.67) | 235.76(192.30,298.29) | -0.17(-0.27,-0.06) | 1.02(0.74–1.40) |
| Caribbean | 6246.08(5065.06,7852.78) | 316.59(256.73,398.03) | 0.19(0.14,0.24) | 6431.30(5149.07,8229.63) | 339.33(271.68,434.22) | 0.23(0.19,0.28) | 0.93(0.68–1.29) |
| Andean Latin America | 12380.39(9124.66,18183.98) | 391.92(288.86,575.64) | -0.32(-0.37,-0.27) | 7646.60(5784.31,10891.97) | 255.15(193.01,363.44) | -0.27(-0.33,-0.21) | 1.54(0.96–2.45) |
| Central Latin America | 34167.63(25422.69,49192.66) | 334.32(248.75,481.33) | -0.40(-0.48,-0.32) | 22915.88(18083.77,29623.00) | 232.17(183.21,300.12) | -0.34(-0.43,-0.26) | 1.44(0.95–2.17) |
| Tropical Latin America | 31664.91(22838.10,45863.47) | 359.59(259.35,520.82) | -0.05(-0.11,0.02) | 24164.79(18297.21,33292.98) | 287.62(217.78,396.27) | -0.01(-0.08,0.05) | 1.25(0.79–1.98) |
| North Africa and Middle East | 95640.90(79387.79,117791.91) | 303.98(252.32,374.38) | -0.06(-0.14,0.01) | 85363.09(70437.10,105513.08) | 287.67(237.37,355.57) | -0.12(-0.25,0.00) | 1.06(0.80–1.40) |
| South Asia | 310970.17(243576.52,406327.98) | 376.20(294.67,491.56) | -0.25(-0.31,-0.19) | 286037.31(225991.68,369505.85) | 376.70(297.62,486.63) | -0.23(-0.26,-0.20) | 1.00(0.70–1.42) |
| Central Sub-Saharan Africa | 47544.39(36295.08,63637.80) | 444.47(339.30,594.92) | -0.75(-0.84,-0.66) | 48484.52(37666.64,63988.33) | 467.57(363.24,617.08) | -0.74(-0.83,-0.65) | 0.95(0.65–1.40) |
| Southern Sub-Saharan Africa | 14508.19(10890.79,20085.21) | 357.17(268.11,494.47) | -0.31(-0.37,-0.24) | 15677.15(12185.55,20744.33) | 395.18(307.17,522.91) | -0.25(-0.34,-0.16) | 0.90(0.60–1.36) |
| Eastern Sub-Saharan Africa | 131731.82(101859.55,177287.00) | 405.75(313.74,546.07) | -0.78(-0.82,-0.74) | 137961.96(106783.95,182633.31) | 440.35(340.83,582.93) | -0.74(-0.77,-0.71) | 0.92(0.63–1.36) |
| Western Sub-Saharan Africa | 194259.05(150280.40,259367.78) | 478.84(370.43,639.33) | -0.58(-0.63,-0.52) | 201380.98(158407.54,262986.80) | 511.26(402.16,667.66) | -0.52(-0.57,-0.47) | 0.94(0.65–1.36) |
| High SDI | 62590.04(51871.52,76919.67) | 226.63(187.82,278.52) | -0.14(-0.27,-0.02) | 58965.93(49131.15,72486.20) | 224.82(187.32,276.37) | -0.17(-0.23,-0.11) | 1.01(0.76–1.33) |
| Low SDI | 381232.04(297862.20,501240.15) | 450.60(352.06,592.45) | -0.54(-0.58,-0.49) | 383766.73(303707.02,492963.51) | 473.96(375.09,608.82) | -0.49(-0.52,-0.46) | 0.95(0.67–1.36) |
| High-middle SDI | 87263.89(69051.24,113137.43) | 237.60(188.01,308.05) | -0.61(-0.82,-0.41) | 75915.18(59829.74,98267.08) | 227.85(179.57,294.94) | -0.60(-0.80,-0.40) | 1.04(0.73–1.48) |
| Middle SDI | 278372.06(212876.23,372276.77) | 302.48(231.32,404.52) | -0.56(-0.67,-0.45) | 235683.45(183051.38,309796.75) | 278.62(216.40,366.24) | -0.52(-0.63,-0.41) | 1.09(0.74–1.59) |
| Low-middle SDI | 381652.48(299653.17,497610.59) | 386.53(303.48,503.97) | -0.32(-0.41,-0.23) | 353181.65(280173.16,455264.62) | 380.42(301.78,490.38) | -0.27(-0.29,-0.24) | 1.02(0.72–1.44) |

DALYs = disability-adjusted life years; EAPC = estimated annual percentage change.
