# Supplementary material for: Sex differences in global burden of Congenital Heart Anomalies in children under five from 1990 to 2021
Source: PLoS One. 2026 May 6;21(5):e0348351. doi: 10.1371/journal.pone.0348351 (PMC13148693; doi:10.1371/journal.pone.0348351)
Supplement: S7 Table — (DOCX) [file pone.0348351.s007.docx]

**Supplementary Table 7.** National Trends in Disability-Adjusted Life Years Due to Congenital Heart Anomalies among Children Under 5 Years, 2021, and Estimated Annual Percentage Change, 1990–2021.

| location | Male | | | Female | | |
| --- | --- | --- | --- | --- | --- | --- |
|  | 2021 | | EAPC from 1990 to 2021 | 2021 | | EAPC from 1990 to 2021 |
|  | DALYs number  (95% UI) | DALYs rate  (95% UI) | rate  (95% CI) | DALYs number  (95% UI) | DALYs rate  (95% UI) | rate  (95% CI) |
| Afghanistan | 395528.97(167622.74,581049.04) | 13879.67(5882.12,20389.83) | -2.48(-2.78,-2.17) | 325334.72(139928.21,503788.43) | 12361.95(5316.94,19142.76) | -2.64(-2.81,-2.47) |
| Albania | 2065.83(1207.73,3191.13) | 2784.19(1627.70,4300.79) | -1.81(-1.96,-1.66) | 1331.29(721.04,2164.71) | 1946.21(1054.08,3164.59) | -1.86(-2.03,-1.68) |
| Algeria | 103969.13(74758.06,147069.65) | 4287.86(3083.14,6065.39) | -3.66(-4.28,-3.04) | 77223.21(49777.15,108099.65) | 3387.07(2183.27,4741.34) | -3.89(-4.16,-3.62) |
| American Samoa | 18.80(10.28,33.49) | 984.77(538.57,1753.94) | -1.01(-1.50,-0.52) | 28.18(18.45,42.57) | 1565.88(1025.26,2365.13) | -0.83(-1.13,-0.54) |
| Andorra | 2.44(1.64,3.65) | 189.16(127.02,282.41) | -5.04(-5.24,-4.85) | 3.41(2.23,4.88) | 276.48(180.73,395.95) | -5.08(-5.52,-4.63) |
| Angola | 92635.01(45526.49,162541.90) | 3264.29(1604.27,5727.69) | -3.01(-3.16,-2.87) | 61854.10(35579.40,100008.96) | 2212.73(1272.80,3577.66) | -3.21(-3.50,-2.93) |
| Antigua and Barbuda | 39.82(27.59,48.81) | 1481.99(1027.02,1816.84) | -0.55(-0.99,-0.12) | 54.53(45.28,64.09) | 2109.12(1751.34,2478.69) | -0.44(-0.73,-0.15) |
| Argentina | 32253.98(24685.53,41219.85) | 2106.74(1612.39,2692.37) | -1.77(-2.18,-1.36) | 27917.07(21746.07,34961.35) | 1905.24(1484.09,2385.99) | -1.80(-2.11,-1.48) |
| Armenia | 2675.14(1982.12,3467.89) | 2727.37(2020.81,3535.59) | -1.32(-1.78,-0.86) | 1686.25(1129.88,2248.21) | 1913.50(1282.15,2551.20) | -1.08(-1.99,-0.16) |
| Australia | 4313.82(2977.72,5667.94) | 557.87(385.08,732.99) | -2.63(-2.81,-2.46) | 2864.86(2133.21,3596.75) | 392.35(292.15,492.58) | -2.79(-2.99,-2.58) |
| Austria | 1447.04(1093.11,2055.54) | 649.50(490.64,922.62) | -3.97(-4.11,-3.82) | 973.74(764.42,1152.25) | 465.18(365.19,550.47) | -4.09(-4.61,-3.57) |
| Azerbaijan | 13524.45(8096.77,24033.25) | 3526.17(2111.04,6266.09) | -1.60(-1.75,-1.44) | 10054.41(5379.15,16436.12) | 2979.08(1593.82,4869.95) | -1.69(-2.13,-1.25) |
| Bahamas | 210.62(143.12,288.10) | 1966.67(1336.43,2690.13) | -1.13(-1.34,-0.92) | 125.09(92.06,167.02) | 1209.05(889.77,1614.31) | -0.89(-1.20,-0.58) |
| Bahrain | 512.39(373.43,689.54) | 1069.11(779.17,1438.74) | -5.13(-6.10,-4.14) | 503.03(359.71,680.89) | 1099.19(786.03,1487.84) | -5.14(-5.68,-4.60) |
| Bangladesh | 163129.80(62201.83,323367.16) | 2212.41(843.60,4385.59) | -4.82(-5.19,-4.45) | 147182.70(72339.64,321666.87) | 2105.42(1034.80,4601.38) | -4.77(-5.01,-4.53) |
| Barbados | 150.71(100.95,223.27) | 2161.41(1447.78,3201.93) | -1.09(-1.25,-0.94) | 169.46(121.37,234.29) | 2550.54(1826.70,3526.30) | -0.88(-1.15,-0.61) |
| Belarus | 1420.89(891.94,3093.57) | 590.81(370.87,1286.31) | -7.03(-7.79,-6.26) | 1076.34(784.73,1499.11) | 474.45(345.91,660.81) | -7.78(-9.01,-6.54) |
| Belgium | 2283.42(1700.45,2913.44) | 754.30(561.72,962.41) | -3.35(-3.48,-3.22) | 1463.51(1104.26,1796.92) | 506.03(381.81,621.31) | -3.68(-4.17,-3.20) |
| Belize | 413.09(321.67,524.60) | 2140.73(1666.99,2718.61) | -1.66(-1.81,-1.51) | 256.22(199.16,327.10) | 1360.27(1057.34,1736.59) | -1.70(-2.07,-1.32) |
| Benin | 57163.95(25992.46,92935.75) | 4789.14(2177.62,7786.07) | -1.57(-1.79,-1.34) | 50411.97(27553.86,78710.90) | 4388.43(2398.60,6851.89) | -1.53(-1.71,-1.36) |
| Bermuda | 13.15(6.27,21.92) | 1009.19(480.86,1682.21) | -2.15(-2.37,-1.94) | 6.95(3.49,16.05) | 556.82(279.62,1285.67) | -2.23(-2.46,-1.99) |
| Bhutan | 899.52(357.71,1702.86) | 2901.27(1153.74,5492.31) | -3.72(-4.30,-3.12) | 735.13(376.36,1371.90) | 2448.33(1253.44,4569.05) | -3.92(-4.16,-3.68) |
| Bolivia (Plurinational State of) | 39029.60(26722.96,53838.01) | 6407.00(4386.77,8837.91) | -2.85(-3.09,-2.62) | 28070.30(18304.91,39094.81) | 4798.23(3128.97,6682.71) | -2.97(-3.12,-2.83) |
| Bosnia and Herzegovina | 721.29(517.97,967.59) | 925.23(664.43,1241.17) | -3.12(-3.33,-2.92) | 559.79(371.42,890.13) | 765.97(508.21,1217.99) | -3.47(-3.82,-3.11) |
| Botswana | 1994.03(1151.63,3115.23) | 1668.39(963.56,2606.48) | -0.49(-0.66,-0.33) | 1273.44(678.69,2016.15) | 1097.99(585.18,1738.37) | -0.41(-0.51,-0.32) |
| Brazil | 218533.19(172164.22,278085.23) | 2579.46(2032.14,3282.38) | -0.82(-0.96,-0.67) | 169450.66(130321.53,210037.58) | 2095.74(1611.80,2597.71) | -0.74(-1.17,-0.32) |
| Brunei Darussalam | 295.12(199.20,416.06) | 1839.36(1241.50,2593.09) | -0.54(-0.98,-0.09) | 283.02(195.46,382.90) | 1905.80(1316.18,2578.36) | -0.42(-0.62,-0.22) |
| Bulgaria | 2527.14(1922.56,3210.84) | 1624.02(1235.50,2063.39) | -4.21(-4.43,-3.99) | 2317.46(1806.48,2850.80) | 1575.74(1228.30,1938.38) | -4.21(-4.87,-3.55) |
| Burkina Faso | 140767.87(48586.40,230100.16) | 6766.24(2335.39,11060.15) | -1.18(-1.67,-0.69) | 131089.15(56868.03,222312.74) | 6470.28(2806.88,10972.88) | -0.99(-1.12,-0.86) |
| Burundi | 34921.13(16001.16,62402.02) | 3216.13(1473.66,5747.04) | -2.26(-2.40,-2.12) | 26366.75(11264.30,46063.68) | 2453.69(1048.26,4286.68) | -2.38(-2.85,-1.91) |
| Cabo Verde | 230.73(117.67,396.76) | 1028.47(524.51,1768.57) | -5.23(-5.55,-4.90) | 277.94(143.40,508.84) | 1285.26(663.10,2352.97) | -5.19(-5.51,-4.88) |
| Cambodia | 60480.05(34302.98,93130.45) | 6767.89(3838.60,10421.57) | -3.04(-3.35,-2.74) | 44792.59(29404.52,62674.04) | 5239.43(3439.47,7331.04) | -3.23(-3.45,-3.01) |
| Cameroon | 100152.76(39251.65,157034.55) | 4031.34(1579.95,6320.94) | -1.50(-1.70,-1.31) | 74806.59(41031.67,110245.91) | 3143.98(1724.48,4633.43) | -1.30(-1.50,-1.09) |
| Canada | 6823.97(5398.29,8795.67) | 700.77(554.36,903.25) | -2.97(-3.26,-2.68) | 4612.18(3658.73,5585.06) | 497.91(394.98,602.94) | -3.13(-3.50,-2.75) |
| Central African Republic | 25428.05(7861.85,49132.32) | 5949.72(1839.54,11496.11) | -1.38(-1.54,-1.22) | 16489.55(6657.70,29024.05) | 4007.56(1618.07,7053.91) | -1.08(-1.24,-0.93) |
| Chad | 113493.66(26540.77,193326.15) | 6123.74(1432.05,10431.24) | -0.58(-0.92,-0.24) | 81439.16(34095.52,132573.70) | 4569.64(1913.14,7438.86) | -0.45(-0.54,-0.37) |
| Chile | 5387.65(4360.06,6740.01) | 972.03(786.64,1216.02) | -2.30(-2.44,-2.16) | 4674.86(3820.25,5550.16) | 875.94(715.81,1039.95) | -2.46(-2.95,-1.96) |
| China | 622895.43(447881.13,903941.00) | 1496.24(1075.84,2171.34) | -5.77(-6.21,-5.32) | 474882.32(359931.19,682679.76) | 1317.74(998.76,1894.35) | -5.87(-6.27,-5.46) |
| Colombia | 48862.43(32895.19,70788.48) | 2771.04(1865.52,4014.50) | -1.26(-1.42,-1.09) | 36034.10(24008.20,50564.17) | 2145.60(1429.53,3010.77) | -1.01(-1.45,-0.57) |
| Comoros | 1288.72(616.04,2507.09) | 3102.01(1482.84,6034.68) | -2.56(-2.93,-2.18) | 883.82(457.18,1794.02) | 2222.77(1149.79,4511.85) | -2.76(-2.97,-2.55) |
| Congo | 6691.21(3794.38,11405.10) | 2087.48(1183.75,3558.09) | -2.98(-3.06,-2.90) | 4229.82(2687.55,7135.26) | 1354.40(860.56,2284.73) | -3.14(-3.51,-2.77) |
| Cook Islands | 3.11(0.92,10.00) | 532.23(157.59,1714.09) | -6.18(-6.56,-5.79) | 2.78(1.54,8.77) | 513.95(284.03,1621.83) | -6.46(-7.23,-5.67) |
| Costa Rica | 2895.21(2162.96,3904.07) | 1841.61(1375.84,2483.34) | -1.71(-2.04,-1.38) | 2549.28(2013.04,3103.39) | 1687.25(1332.33,2053.99) | -1.76(-2.14,-1.38) |
| Côte d'Ivoire | 91993.70(40680.70,151123.11) | 4128.67(1825.75,6782.39) | -4.22(-4.78,-3.66) | 71164.29(42423.71,107576.62) | 3346.98(1995.26,5059.52) | -4.26(-4.62,-3.90) |
| Croatia | 807.61(581.92,1119.59) | 861.55(620.78,1194.36) | -4.30(-4.67,-3.93) | 591.09(455.87,754.96) | 667.84(515.06,852.99) | -4.30(-4.49,-4.11) |
| Cuba | 2684.40(1978.39,3812.24) | 953.07(702.41,1353.50) | -6.23(-7.11,-5.33) | 2333.99(1836.68,2855.64) | 888.10(698.87,1086.59) | -6.51(-6.83,-6.19) |
| Cyprus | 134.74(84.88,197.06) | 347.91(219.19,508.84) | -5.80(-6.16,-5.43) | 157.19(104.70,245.17) | 432.84(288.31,675.12) | -6.01(-6.44,-5.58) |
| Czechia | 1081.61(706.60,1535.66) | 375.85(245.54,533.62) | -1.58(-1.83,-1.34) | 673.06(489.79,849.07) | 245.39(178.57,309.55) | -1.55(-1.76,-1.34) |
| Democratic People's Republic of Korea | 16786.30(9461.59,31379.63) | 2169.62(1222.91,4055.80) | -2.96(-3.16,-2.75) | 14842.74(9026.87,26037.12) | 2007.34(1220.80,3521.28) | -3.13(-3.51,-2.74) |
| Democratic Republic of the Congo | 166627.75(85480.39,307424.07) | 2413.34(1238.05,4452.54) | -2.75(-3.01,-2.48) | 139728.63(61391.55,255736.52) | 2099.20(922.31,3842.03) | -2.91(-3.24,-2.58) |
| Denmark | 1231.47(872.19,1535.02) | 772.57(547.17,963.00) | -5.36(-5.86,-4.86) | 675.98(502.22,819.47) | 446.80(331.95,541.64) | -5.19(-5.78,-4.61) |
| Djibouti | 1787.90(832.08,3417.89) | 2328.09(1083.49,4450.56) | -2.62(-2.76,-2.49) | 1211.58(561.40,2366.10) | 1758.49(814.82,3434.17) | -2.78(-3.13,-2.42) |
| Dominica | 56.65(35.01,87.48) | 3205.38(1980.93,4949.39) | 1.14(0.84,1.44) | 65.79(36.35,101.32) | 3845.45(2124.81,5921.74) | 1.85(1.42,2.28) |
| Dominican Republic | 11115.99(5094.30,23490.97) | 2106.87(965.55,4452.36) | -2.49(-2.76,-2.21) | 8857.66(4803.32,18447.45) | 1752.20(950.18,3649.23) | -2.69(-3.01,-2.38) |
| Ecuador | 26643.78(19786.84,35135.93) | 3145.79(2336.20,4148.44) | -0.39(-0.70,-0.08) | 21585.56(15527.46,28790.20) | 2651.27(1907.18,3536.19) | -0.14(-0.43,0.15) |
| Egypt | 234240.88(166494.15,325845.48) | 3503.63(2490.31,4873.79) | -4.72(-5.07,-4.37) | 197105.21(134684.91,272514.83) | 3103.17(2120.44,4290.40) | -4.75(-5.06,-4.45) |
| El Salvador | 7068.50(3845.18,12087.42) | 2275.83(1238.02,3891.76) | -4.18(-4.58,-3.77) | 6562.93(4327.52,9693.30) | 2262.01(1491.54,3340.93) | -4.18(-4.47,-3.89) |
| Equatorial Guinea | 2174.03(1151.81,3832.74) | 2192.36(1161.52,3865.05) | -3.69(-4.12,-3.26) | 1120.65(622.33,1908.97) | 1270.94(705.79,2165.00) | -3.89(-4.11,-3.68) |
| Eritrea | 16031.55(8052.35,30840.07) | 3385.21(1700.33,6512.17) | -1.85(-2.05,-1.66) | 11343.11(5139.52,23296.28) | 2552.11(1156.35,5241.48) | -1.89(-2.06,-1.73) |
| Estonia | 150.04(89.88,249.18) | 422.11(252.86,701.00) | -6.44(-7.11,-5.76) | 109.94(76.32,134.36) | 326.90(226.95,399.53) | -6.85(-7.62,-6.08) |
| Eswatini | 1228.34(722.90,2119.73) | 1707.62(1004.96,2946.82) | -1.05(-2.25,0.17) | 994.99(579.74,1580.22) | 1452.90(846.55,2307.46) | -0.85(-1.26,-0.43) |
| Ethiopia | 254114.61(118499.79,487143.52) | 3108.62(1449.63,5959.31) | -3.17(-3.48,-2.86) | 193946.20(106503.40,356814.21) | 2489.49(1367.07,4580.05) | -3.55(-3.69,-3.42) |
| Fiji | 2183.45(1461.59,3186.07) | 4642.15(3107.43,6773.79) | -0.14(-0.33,0.06) | 1451.92(1016.08,2078.38) | 3299.07(2308.75,4722.51) | 0.08(-0.23,0.39) |
| Finland | 718.89(454.84,1073.65) | 578.31(365.90,863.70) | -4.65(-5.09,-4.21) | 525.45(385.49,641.99) | 443.45(325.33,541.81) | -4.57(-4.80,-4.34) |
| France | 10366.32(7535.29,15328.12) | 574.54(417.63,849.54) | -4.14(-4.43,-3.84) | 8536.63(6686.24,10539.33) | 493.55(386.57,609.34) | -4.18(-4.44,-3.92) |
| Gabon | 2098.97(1221.36,3825.32) | 1950.92(1135.21,3555.50) | -1.70(-1.90,-1.49) | 1089.71(609.57,1849.32) | 1028.51(575.33,1745.46) | -1.73(-2.20,-1.26) |
| Gambia | 4242.75(2589.22,6570.82) | 2330.29(1422.11,3608.97) | -2.61(-2.74,-2.48) | 4145.25(2533.83,6190.52) | 2361.69(1443.61,3526.95) | -2.78(-3.00,-2.56) |
| Georgia | 2551.98(1838.74,3317.47) | 2022.78(1457.44,2629.53) | 0.44(-0.28,1.16) | 1805.33(1049.33,2401.84) | 1541.29(895.86,2050.55) | 0.51(-0.05,1.08) |
| Germany | 14000.13(10559.08,18006.73) | 674.81(508.95,867.93) | -3.48(-3.55,-3.40) | 11427.84(9003.23,13357.45) | 580.45(457.30,678.46) | -3.76(-4.03,-3.48) |
| Ghana | 56100.36(30309.30,85545.91) | 2364.21(1277.31,3605.12) | -1.71(-2.76,-0.65) | 46627.65(27091.05,70487.63) | 2056.59(1194.89,3108.97) | -1.73(-1.97,-1.50) |
| Greece | 2133.35(1591.11,2795.25) | 984.45(734.23,1289.89) | -4.64(-4.89,-4.39) | 1636.47(1337.32,1989.22) | 794.76(649.47,966.07) | -4.56(-5.03,-4.08) |
| Greenland | 14.45(7.69,32.24) | 687.21(365.70,1533.30) | -5.47(-6.00,-4.95) | 14.31(8.96,26.26) | 738.10(461.87,1354.13) | -5.54(-5.73,-5.34) |
| Grenada | 93.63(69.96,121.20) | 2660.84(1988.19,3444.36) | -0.63(-0.99,-0.27) | 86.88(67.76,109.09) | 2594.63(2023.65,3257.87) | -0.46(-0.70,-0.22) |
| Guam | 106.34(68.60,148.42) | 1603.64(1034.57,2238.23) | 0.56(0.30,0.82) | 71.52(47.88,126.74) | 1163.36(778.84,2061.48) | 0.83(0.24,1.42) |
| Guatemala | 28008.87(18841.32,38629.38) | 3543.60(2383.75,4887.28) | 3.69(2.87,4.52) | 19509.27(14064.06,25677.23) | 2537.43(1829.21,3339.65) | 4.05(3.16,4.95) |
| Guinea | 64598.25(25939.08,99529.60) | 5622.13(2257.54,8662.29) | -1.84(-2.25,-1.42) | 54234.41(27966.32,84920.21) | 4923.79(2538.98,7709.67) | -1.87(-2.06,-1.69) |
| Guinea-Bissau | 5735.45(2914.82,8893.15) | 3383.45(1719.51,5246.25) | -3.12(-3.69,-2.54) | 5144.99(2849.13,8453.14) | 3153.71(1746.43,5181.50) | -3.36(-3.73,-2.98) |
| Guyana | 1099.44(726.04,1565.57) | 2878.85(1901.11,4099.38) | 0.03(-0.32,0.38) | 864.88(634.74,1153.49) | 2381.13(1747.52,3175.73) | 0.14(-0.17,0.44) |
| Haiti | 90239.33(52213.71,143121.66) | 11338.43(6560.57,17983.00) | -1.59(-1.82,-1.36) | 67573.86(30505.78,128084.02) | 8730.75(3941.44,16548.85) | -1.69(-1.95,-1.43) |
| Honduras | 16016.15(9992.37,25080.29) | 2864.01(1786.84,4484.86) | -2.88(-3.14,-2.62) | 9733.00(5803.51,17964.65) | 1814.84(1082.14,3349.73) | -3.00(-3.16,-2.83) |
| Hungary | 1992.26(1392.58,2718.69) | 854.71(597.44,1166.35) | -4.63(-4.89,-4.38) | 1378.65(971.55,1760.50) | 622.94(438.99,795.47) | -4.55(-4.80,-4.29) |
| Iceland | 64.81(42.45,94.20) | 574.29(376.16,834.70) | -3.40(-3.68,-3.12) | 47.50(35.76,58.37) | 444.46(334.64,546.15) | -3.72(-4.14,-3.29) |
| India | 1736329.86(1169883.95,2698135.08) | 2976.01(2005.14,4624.51) | -2.17(-2.35,-1.98) | 1270399.37(851472.88,1933493.64) | 2397.34(1606.79,3648.65) | -2.26(-2.37,-2.15) |
| Indonesia | 385360.81(268202.94,527653.24) | 3431.24(2388.07,4698.21) | -2.38(-2.66,-2.10) | 210854.95(123926.10,302981.08) | 1975.11(1160.83,2838.07) | -2.52(-2.58,-2.45) |
| Iran (Islamic Republic of) | 31000.15(19610.67,48798.24) | 979.16(619.41,1541.32) | -5.86(-6.05,-5.67) | 28260.21(20231.11,42783.74) | 945.44(676.82,1431.31) | -6.11(-7.23,-4.98) |
| Iraq | 88840.63(57165.72,137877.56) | 4016.83(2584.68,6233.98) | -3.74(-4.04,-3.44) | 62499.55(44726.31,88686.15) | 3002.30(2148.53,4260.23) | -4.07(-4.31,-3.83) |
| Ireland | 881.39(650.55,1261.95) | 576.42(425.45,825.30) | -4.12(-4.93,-3.31) | 694.62(540.19,861.67) | 477.43(371.29,592.24) | -4.15(-4.43,-3.86) |
| Israel | 2832.27(2126.66,3924.59) | 600.83(451.14,832.55) | -3.99(-4.34,-3.63) | 1897.30(1437.97,2377.38) | 424.57(321.78,532.00) | -4.11(-4.37,-3.85) |
| Italy | 7716.65(5644.43,9764.89) | 692.02(506.18,875.70) | -4.70(-5.06,-4.33) | 5231.04(3884.60,6397.10) | 495.81(368.19,606.33) | -4.72(-4.91,-4.52) |
| Jamaica | 1751.73(1226.78,2450.61) | 2013.21(1409.90,2816.41) | -0.72(-0.92,-0.51) | 1471.91(1080.99,1944.60) | 1748.75(1284.30,2310.34) | -0.63(-0.97,-0.29) |
| Japan | 13914.23(8885.05,19873.42) | 591.55(377.74,844.91) | -4.40(-4.66,-4.14) | 9813.47(7520.88,11893.02) | 439.42(336.76,532.53) | -4.36(-4.52,-4.19) |
| Jordan | 16834.11(11873.14,24337.21) | 2982.80(2103.78,4312.26) | -3.72(-3.95,-3.50) | 13158.68(9926.16,17474.20) | 2469.06(1862.52,3278.81) | -3.93(-4.04,-3.81) |
| Kazakhstan | 31107.90(23422.69,40811.56) | 3097.20(2332.03,4063.32) | -1.41(-1.55,-1.27) | 22599.22(14763.84,28614.82) | 2393.86(1563.88,3031.07) | -1.10(-2.14,-0.04) |
| Kenya | 49938.43(27525.31,92434.42) | 1651.35(910.20,3056.60) | -1.89(-2.31,-1.47) | 29831.35(14791.56,76078.10) | 1019.50(505.51,2599.99) | -1.98(-2.24,-1.71) |
| Kiribati | 352.41(108.44,549.79) | 4754.51(1462.97,7417.41) | -1.94(-2.10,-1.79) | 235.19(99.52,382.63) | 3418.26(1446.42,5561.19) | -2.01(-2.11,-1.90) |
| Kuwait | 2862.45(2238.78,3716.00) | 2103.18(1644.93,2730.32) | -2.91(-3.11,-2.72) | 1975.09(1596.59,2467.86) | 1532.11(1238.50,1914.37) | -3.10(-3.72,-2.47) |
| Kyrgyzstan | 12809.25(9966.48,16209.17) | 3145.40(2447.34,3980.28) | -0.10(-0.35,0.14) | 10213.75(7171.30,12734.35) | 2641.52(1854.67,3293.41) | 0.14(-0.45,0.72) |
| Lao People's Democratic Republic | 34592.97(15976.20,56633.70) | 8176.40(3776.14,13385.95) | -2.75(-2.88,-2.62) | 27494.87(14479.20,42183.64) | 6764.64(3562.36,10378.55) | -2.94(-3.12,-2.77) |
| Latvia | 273.84(193.21,458.03) | 565.15(398.74,945.27) | -6.05(-6.28,-5.82) | 243.35(193.31,292.06) | 537.68(427.12,645.31) | -6.39(-7.17,-5.60) |
| Lebanon | 2492.26(1552.82,3682.67) | 1180.39(735.45,1744.19) | -4.87(-5.37,-4.38) | 2249.84(1413.78,3698.76) | 1155.19(725.91,1899.14) | -4.90(-5.19,-4.61) |
| Lesotho | 2733.57(1326.13,4797.25) | 2660.24(1290.55,4668.56) | -0.65(-0.86,-0.45) | 1514.92(814.99,2491.64) | 1500.55(807.26,2468.00) | -0.47(-0.61,-0.33) |
| Liberia | 15118.16(6415.02,24444.59) | 3852.68(1634.79,6229.41) | -3.56(-4.26,-2.86) | 12621.77(6978.79,20275.63) | 3374.97(1866.08,5421.56) | -3.85(-4.22,-3.47) |
| Libya | 13699.55(7046.25,20809.62) | 6327.23(3254.35,9611.06) | -1.54(-1.91,-1.17) | 7617.78(3733.88,12056.35) | 3687.97(1807.67,5836.79) | -1.50(-2.19,-0.81) |
| Lithuania | 658.53(458.81,955.92) | 974.98(679.29,1415.29) | -4.52(-4.94,-4.10) | 370.69(272.32,450.32) | 578.74(425.15,703.05) | -4.50(-5.01,-3.99) |
| Luxembourg | 65.05(42.76,108.85) | 385.33(253.26,644.75) | -4.48(-4.78,-4.17) | 44.27(35.21,54.63) | 273.54(217.60,337.61) | -4.48(-5.21,-3.74) |
| Madagascar | 68163.97(31266.52,128373.42) | 3283.00(1505.90,6182.88) | -1.78(-1.97,-1.59) | 41179.22(18954.09,85168.87) | 2046.90(942.15,4233.49) | -1.80(-2.01,-1.58) |
| Malawi | 44736.99(24101.32,80976.46) | 3260.65(1756.62,5901.95) | -3.27(-3.58,-2.96) | 28817.18(15130.23,55366.05) | 2131.41(1119.08,4095.04) | -3.56(-3.72,-3.39) |
| Malaysia | 14058.24(9372.97,20112.80) | 1108.25(738.90,1585.55) | -2.01(-2.15,-1.87) | 11854.60(8567.97,16701.78) | 996.26(720.05,1403.61) | -2.04(-2.73,-1.35) |
| Maldives | 284.70(181.04,424.39) | 1746.29(1110.48,2603.15) | -4.11(-4.45,-3.77) | 287.78(203.17,423.75) | 1862.00(1314.58,2741.80) | -4.14(-4.32,-3.95) |
| Mali | 136111.04(76157.12,202752.05) | 5837.89(3266.43,8696.16) | -2.66(-2.77,-2.54) | 137740.53(69172.66,216291.02) | 6124.88(3075.89,9617.77) | -2.80(-2.95,-2.65) |
| Malta | 113.00(82.42,153.45) | 993.27(724.46,1348.88) | -2.08(-2.55,-1.61) | 108.79(86.04,135.60) | 1023.83(809.69,1276.07) | -2.13(-2.64,-1.62) |
| Marshall Islands | 79.31(47.05,125.01) | 2705.03(1604.71,4263.86) | -1.29(-1.71,-0.87) | 52.83(32.79,79.54) | 1927.16(1196.10,2901.27) | -1.04(-1.46,-0.61) |
| Mauritania | 7036.73(4031.53,11039.66) | 2100.49(1203.42,3295.37) | -2.72(-2.99,-2.45) | 5630.62(3295.27,8282.19) | 1748.66(1023.39,2572.13) | -2.83(-3.25,-2.41) |
| Mauritius | 906.30(678.51,1276.64) | 2773.70(2076.55,3907.11) | -1.28(-3.19,0.67) | 717.63(560.09,973.65) | 2269.50(1771.27,3079.17) | -1.02(-1.23,-0.81) |
| Mexico | 199978.50(142185.06,269527.06) | 4001.55(2845.11,5393.21) | -0.45(-0.80,-0.09) | 155065.51(111724.00,198810.63) | 3177.83(2289.62,4074.33) | -0.34(-0.63,-0.04) |
| Micronesia (Federated States of) | 97.31(66.69,144.10) | 1988.51(1362.83,2944.74) | -2.90(-3.24,-2.56) | 71.98(45.70,108.49) | 1572.80(998.53,2370.35) | -3.07(-3.18,-2.96) |
| Monaco | 5.06(2.94,8.92) | 620.95(360.81,1093.65) | -4.08(-4.58,-3.59) | 7.10(4.84,10.60) | 884.10(603.13,1320.27) | -4.13(-4.66,-3.59) |
| Mongolia | 4338.10(2518.94,6448.56) | 2161.02(1254.80,3212.34) | -3.57(-3.89,-3.25) | 4249.43(2886.49,6006.02) | 2237.10(1519.59,3161.86) | -3.88(-4.07,-3.68) |
| Montenegro | 64.96(33.14,123.82) | 345.70(176.35,658.93) | -5.53(-6.09,-4.96) | 74.47(47.78,135.79) | 427.99(274.61,780.39) | -5.68(-6.22,-5.13) |
| Morocco | 27779.65(15674.87,58725.48) | 1668.23(941.31,3526.59) | -4.66(-4.94,-4.38) | 20847.60(10937.49,50712.66) | 1314.83(689.81,3198.38) | -4.57(-4.94,-4.21) |
| Mozambique | 110481.13(48688.88,213179.93) | 4230.12(1864.21,8162.27) | -2.84(-3.14,-2.53) | 81582.03(38656.65,151374.81) | 3177.56(1505.65,5895.94) | -2.95(-3.10,-2.79) |
| Myanmar | 241095.79(111441.72,376167.12) | 9021.70(4170.10,14076.01) | -2.30(-2.45,-2.16) | 164380.24(90821.05,242966.10) | 6436.16(3556.02,9513.12) | -2.51(-2.79,-2.22) |
| Namibia | 1940.97(1075.00,3296.89) | 1382.24(765.55,2347.85) | -1.40(-1.87,-0.92) | 1568.38(973.75,2513.45) | 1136.26(705.46,1820.95) | -1.09(-1.36,-0.82) |
| Nauru | 27.18(14.64,42.94) | 3717.34(2002.20,5872.11) | -0.75(-0.96,-0.55) | 18.62(10.54,26.99) | 2792.67(1581.14,4049.08) | -0.71(-1.37,-0.04) |
| Nepal | 20727.94(9618.33,54118.31) | 1292.28(599.65,3373.99) | -3.99(-4.40,-3.57) | 22946.59(11909.70,54082.95) | 1527.33(792.71,3599.76) | -4.13(-4.23,-4.02) |
| Netherlands | 2621.30(2089.31,3630.03) | 594.35(473.72,823.06) | -4.66(-5.54,-3.79) | 1797.75(1515.49,2130.55) | 427.85(360.67,507.05) | -4.64(-4.98,-4.29) |
| New Zealand | 859.72(573.19,1316.32) | 535.71(357.16,820.21) | -3.36(-3.52,-3.20) | 731.79(593.16,861.00) | 481.08(389.94,566.02) | -3.68(-4.27,-3.09) |
| Nicaragua | 7776.96(4670.57,12760.88) | 2329.65(1399.10,3822.62) | -3.55(-3.62,-3.49) | 6335.47(4189.90,9176.23) | 2000.41(1322.95,2897.37) | -3.79(-4.00,-3.57) |
| Niger | 106250.00(34900.57,169433.71) | 4089.12(1343.18,6520.80) | -2.57(-2.71,-2.43) | 113319.11(46017.93,186028.80) | 4536.42(1842.20,7447.16) | -2.77(-2.99,-2.56) |
| Nigeria | 985713.69(368299.28,1565781.61) | 5264.41(1966.98,8362.39) | -0.87(-0.92,-0.82) | 805336.59(389320.14,1326250.26) | 4378.32(2116.59,7210.33) | -0.83(-1.00,-0.66) |
| Niue | 5.16(3.63,7.13) | 8498.77(5974.63,11755.98) | 0.82(0.55,1.09) | 4.27(3.08,6.20) | 7528.13(5430.61,10914.19) | 0.84(0.00,1.69) |
| North Macedonia | 442.35(271.48,753.55) | 851.03(522.29,1449.75) | -5.50(-5.99,-5.00) | 331.60(226.60,527.57) | 683.14(466.84,1086.88) | -5.55(-6.06,-5.04) |
| Northern Mariana Islands | 11.92(8.01,18.01) | 717.03(481.60,1083.69) | -1.49(-1.85,-1.12) | 10.74(6.86,16.68) | 695.50(444.08,1079.60) | -1.26(-1.72,-0.79) |
| Norway | 658.49(422.61,1205.02) | 456.04(292.68,834.55) | -4.88(-5.36,-4.40) | 429.82(302.99,558.64) | 314.37(221.61,408.59) | -4.99(-5.33,-4.65) |
| Oman | 3460.62(2340.23,5015.19) | 1599.55(1081.69,2318.10) | -3.12(-3.34,-2.90) | 3167.92(2253.66,4545.31) | 1525.44(1085.20,2188.69) | -3.36(-4.33,-2.37) |
| Pakistan | 492555.79(280083.92,741073.25) | 3217.44(1829.55,4840.80) | -1.43(-1.86,-1.01) | 432553.94(231128.57,685378.17) | 3000.29(1603.16,4753.93) | -1.18(-1.50,-0.86) |
| Palau | 12.26(8.09,17.58) | 2480.07(1636.32,3554.52) | -1.76(-1.97,-1.54) | 6.96(4.23,9.98) | 1537.63(934.00,2205.38) | -1.79(-2.00,-1.58) |
| Palestine | 7456.56(4941.92,10964.78) | 2373.11(1572.80,3489.62) | -4.24(-4.48,-4.00) | 6544.79(4596.92,9454.89) | 2192.22(1539.77,3166.98) | -4.29(-4.59,-3.98) |
| Panama | 8077.97(5761.39,10590.08) | 4232.67(3018.83,5548.96) | -1.09(-1.29,-0.90) | 6333.51(4818.40,7994.30) | 3511.11(2671.18,4431.80) | -0.85(-1.02,-0.68) |
| Papua New Guinea | 71166.23(18002.63,116935.04) | 8952.15(2264.59,14709.50) | -0.45(-0.64,-0.26) | 50568.82(18166.56,83286.10) | 6962.05(2501.08,11466.39) | -0.39(-0.56,-0.21) |
| Paraguay | 9338.78(5219.54,15148.82) | 2796.80(1563.16,4536.81) | -1.15(-1.77,-0.52) | 7456.91(4771.86,11356.97) | 2358.59(1509.32,3592.16) | -0.96(-1.23,-0.70) |
| Peru | 37427.95(21435.93,58117.18) | 2198.08(1258.89,3413.12) | -3.46(-3.65,-3.26) | 32731.24(19742.64,46783.04) | 2048.60(1235.66,2928.08) | -3.72(-4.06,-3.39) |
| Philippines | 184830.60(136113.44,274798.79) | 3173.02(2336.68,4717.52) | -2.01(-2.14,-1.89) | 140979.48(109710.63,187792.06) | 2616.39(2036.08,3485.17) | -2.08(-2.26,-1.90) |
| Poland | 11672.87(8610.54,15451.28) | 1207.97(891.06,1598.98) | -4.57(-4.74,-4.41) | 9032.07(6776.44,11061.66) | 986.30(739.99,1207.93) | -4.52(-4.84,-4.19) |
| Portugal | 1453.96(1028.73,1875.00) | 669.74(473.87,863.69) | -5.76(-5.94,-5.57) | 991.20(763.63,1216.47) | 476.00(366.71,584.18) | -5.85(-6.28,-5.41) |
| Puerto Rico | 797.78(600.77,1006.10) | 1479.65(1114.25,1866.01) | -1.85(-2.09,-1.61) | 516.64(366.48,637.71) | 1008.96(715.71,1245.42) | -1.88(-2.26,-1.49) |
| Qatar | 784.98(477.05,1178.26) | 839.48(510.17,1260.07) | -5.38(-5.54,-5.22) | 812.07(573.61,1118.65) | 894.41(631.78,1232.08) | -5.20(-5.33,-5.06) |
| Republic of Korea | 3604.82(2384.76,5128.93) | 455.18(301.12,647.63) | -5.87(-6.50,-5.24) | 3585.49(2534.28,6020.47) | 473.06(334.36,794.32) | -6.12(-6.31,-5.93) |
| Republic of Moldova | 1867.49(1330.18,2571.30) | 2352.09(1675.35,3238.52) | -2.31(-2.59,-2.02) | 1301.20(976.44,1773.70) | 1738.98(1304.96,2370.44) | -2.52(-2.94,-2.09) |
| Romania | 8007.07(6335.37,9563.21) | 1663.49(1316.19,1986.78) | -3.38(-3.84,-2.91) | 6071.10(4911.18,7194.93) | 1329.90(1075.82,1576.08) | -3.70(-4.22,-3.17) |
| Russian Federation | 39199.04(27318.44,56385.97) | 1002.84(698.89,1442.53) | -4.14(-4.68,-3.61) | 28502.40(21260.42,32843.84) | 769.95(574.32,887.23) | -4.18(-5.09,-3.26) |
| Rwanda | 25190.11(13956.55,45209.05) | 2835.46(1570.98,5088.84) | -3.29(-4.08,-2.49) | 19581.80(10006.72,36661.10) | 2276.78(1163.48,4262.59) | -3.59(-3.97,-3.20) |
| Saint Kitts and Nevis | 30.57(21.85,41.87) | 1975.37(1411.56,2705.32) | -0.81(-1.09,-0.52) | 33.49(24.95,44.09) | 2227.41(1659.24,2931.86) | -0.74(-1.00,-0.47) |
| Saint Lucia | 107.04(73.13,152.90) | 2370.79(1619.68,3386.35) | -0.82(-1.11,-0.54) | 54.53(39.38,75.79) | 1262.76(911.89,1755.08) | -0.76(-1.11,-0.42) |
| Saint Vincent and the Grenadines | 67.89(48.82,94.32) | 1857.19(1335.43,2580.26) | -2.47(-2.72,-2.21) | 46.89(34.68,61.38) | 1324.16(979.47,1733.64) | -2.60(-3.00,-2.20) |
| Samoa | 270.48(166.97,415.31) | 1781.56(1099.77,2735.51) | -2.22(-2.42,-2.02) | 235.86(147.39,370.37) | 1677.34(1048.20,2633.89) | -2.37(-2.46,-2.28) |
| San Marino | 1.13(0.63,2.14) | 179.16(100.51,339.25) | -5.58(-5.74,-5.42) | 0.97(0.59,1.87) | 166.62(101.24,322.33) | -5.81(-6.06,-5.55) |
| Sao Tome and Principe | 154.87(87.60,281.47) | 1215.70(687.59,2209.44) | -4.93(-5.17,-4.69) | 204.09(100.91,444.39) | 1672.27(826.82,3641.22) | -4.99(-5.39,-4.59) |
| Saudi Arabia | 9237.14(4220.96,18630.60) | 735.22(335.96,1482.88) | -7.43(-8.58,-6.26) | 8853.19(5146.81,17212.32) | 752.35(437.38,1462.72) | -8.23(-8.31,-8.14) |
| Senegal | 33176.13(20505.13,51438.05) | 2852.32(1762.93,4422.39) | -2.40(-2.54,-2.27) | 31065.55(19145.32,48114.90) | 2802.94(1727.42,4341.25) | -2.59(-2.94,-2.23) |
| Serbia | 1928.08(1257.58,2640.58) | 1010.20(658.90,1383.51) | -7.14(-7.22,-7.05) | 1382.96(992.10,2378.03) | 777.83(557.99,1337.49) | -7.87(-8.65,-7.08) |
| Seychelles | 122.07(86.16,167.50) | 3034.36(2141.69,4163.75) | 0.49(0.28,0.70) | 110.64(72.45,153.82) | 2872.43(1881.10,3993.68) | 0.56(0.30,0.82) |
| Sierra Leone | 45177.50(15254.88,75842.34) | 6648.26(2244.89,11160.86) | -2.22(-2.45,-1.99) | 35019.45(17984.54,55698.95) | 5278.68(2710.91,8395.81) | -2.35(-2.48,-2.22) |
| Singapore | 607.47(373.15,992.56) | 415.55(255.25,678.97) | -6.44(-6.92,-5.95) | 487.34(346.02,619.19) | 348.74(247.61,443.09) | -6.71(-7.28,-6.13) |
| Slovakia | 1817.18(1325.23,2376.95) | 1238.27(903.04,1619.70) | -2.81(-3.21,-2.40) | 1282.76(980.04,1774.95) | 920.34(703.15,1273.47) | -2.94(-3.22,-2.67) |
| Slovenia | 234.87(156.26,329.00) | 465.97(310.01,652.74) | -5.71(-6.13,-5.29) | 158.50(113.67,188.79) | 333.70(239.33,397.48) | -5.84(-6.07,-5.61) |
| Solomon Islands | 1465.45(937.69,2195.66) | 2946.47(1885.33,4414.65) | -1.99(-2.35,-1.63) | 1279.20(720.29,1989.42) | 2805.12(1579.51,4362.55) | -2.01(-2.12,-1.90) |
| Somalia | 93293.57(25577.16,206645.48) | 4364.37(1196.53,9667.10) | -1.52(-1.73,-1.31) | 66522.33(22093.31,153462.77) | 3340.39(1109.41,7706.07) | -1.32(-1.62,-1.02) |
| South Africa | 37068.69(20668.49,59178.23) | 1474.97(822.40,2354.71) | -1.44(-1.87,-1.00) | 32570.24(21202.42,46039.00) | 1330.42(866.07,1880.58) | -1.24(-1.35,-1.12) |
| South Sudan | 65846.54(12827.22,130806.01) | 8156.40(1588.91,16202.91) | -0.41(-0.94,0.13) | 39131.56(14111.73,77188.92) | 5182.75(1869.02,10223.23) | -0.28(-0.72,0.16) |
| Spain | 6589.81(4689.31,8338.37) | 696.44(495.59,881.24) | -5.38(-5.48,-5.29) | 3837.82(2906.92,4629.08) | 428.78(324.77,517.18) | -5.36(-5.76,-4.96) |
| Sri Lanka | 13533.23(8624.17,21171.22) | 1696.88(1081.36,2654.59) | -2.12(-2.69,-1.55) | 11237.59(8045.14,16503.03) | 1463.09(1047.45,2148.63) | -2.14(-2.52,-1.75) |
| Sudan | 290536.70(161132.73,443446.58) | 9980.30(5535.11,15232.94) | -3.25(-3.71,-2.79) | 182134.18(106034.22,272455.96) | 6676.55(3886.93,9987.50) | -3.56(-3.78,-3.34) |
| Suriname | 679.97(405.69,1063.94) | 2989.44(1783.59,4677.51) | -1.87(-2.09,-1.66) | 487.64(290.22,713.99) | 2237.76(1331.82,3276.47) | -1.96(-2.09,-1.83) |
| Sweden | 1437.92(1000.93,2072.61) | 479.81(333.99,691.59) | -4.38(-5.30,-3.44) | 669.42(524.55,1048.05) | 236.12(185.02,369.68) | -4.32(-4.87,-3.76) |
| Switzerland | 1895.02(1456.77,2363.48) | 834.73(641.69,1041.08) | -4.43(-4.79,-4.06) | 1209.14(969.77,1444.40) | 562.46(451.11,671.89) | -4.38(-4.86,-3.89) |
| Syrian Arab Republic | 13123.36(8302.30,20468.32) | 2545.01(1610.06,3969.42) | -3.58(-4.15,-3.00) | 12607.70(7926.40,18175.67) | 2576.05(1619.55,3713.72) | -3.89(-4.72,-3.05) |
| Taiwan (Province of China) | 4060.66(3059.95,5128.41) | 879.81(662.99,1111.16) | -3.41(-3.86,-2.96) | 3103.23(2560.80,3621.79) | 720.51(594.57,840.91) | -3.72(-4.13,-3.31) |
| Tajikistan | 18533.41(9456.69,44978.70) | 2681.07(1368.02,6506.69) | 0.17(-0.29,0.64) | 14235.62(7477.68,28492.69) | 2199.52(1155.36,4402.35) | 0.25(-0.03,0.52) |
| Thailand | 21102.65(11508.44,29147.64) | 1451.47(791.57,2004.81) | -3.30(-3.55,-3.04) | 19812.29(13483.57,26689.26) | 1442.84(981.95,1943.66) | -3.66(-3.88,-3.43) |
| Timor-Leste | 5628.78(3116.20,8651.50) | 5909.99(3271.88,9083.72) | -2.92(-3.22,-2.62) | 4740.87(3000.91,6966.53) | 5290.75(3348.97,7774.55) | -3.12(-3.32,-2.91) |
| Togo | 18562.14(9204.42,29259.99) | 3115.54(1544.91,4911.11) | -2.08(-2.25,-1.90) | 14203.12(8392.06,22764.94) | 2462.75(1455.14,3947.33) | -2.13(-2.31,-1.94) |
| Tokelau | 4.45(2.67,7.44) | 8588.60(5149.86,14357.94) | -1.55(-1.99,-1.10) | 4.35(2.89,7.71) | 9218.40(6137.22,16359.02) | -1.53(-3.25,0.24) |
| Tonga | 119.65(77.88,177.45) | 1588.31(1033.85,2355.69) | -1.72(-1.87,-1.57) | 50.71(31.58,90.55) | 737.63(459.39,1317.06) | -1.78(-2.03,-1.52) |
| Trinidad and Tobago | 1399.95(968.00,1926.92) | 3404.24(2353.87,4685.69) | -0.20(-0.76,0.36) | 1132.73(846.70,1504.77) | 2875.68(2149.55,3820.20) | -0.08(-0.53,0.38) |
| Tunisia | 12231.65(8594.93,17327.37) | 2637.17(1853.08,3735.82) | -4.86(-5.21,-4.50) | 8245.50(5481.55,11462.67) | 1925.58(1280.11,2676.88) | -4.89(-5.08,-4.69) |
| Türkiye | 80749.09(56759.10,110944.13) | 2831.97(1990.61,3890.94) | 2.93(2.16,3.70) | 64918.05(45880.02,86352.93) | 2403.90(1698.93,3197.63) | 3.18(2.29,4.08) |
| Turkmenistan | 16542.07(11615.64,21685.48) | 5976.21(4196.42,7834.39) | -3.94(-4.44,-3.43) | 9964.86(6526.40,14503.05) | 3790.14(2482.32,5516.24) | -4.08(-4.30,-3.86) |
| Tuvalu | 19.66(11.81,30.56) | 2917.00(1751.81,4533.47) | -5.88(-6.25,-5.51) | 10.09(6.43,15.77) | 1655.37(1054.87,2587.55) | -6.31(-6.50,-6.12) |
| Uganda | 126086.85(60898.15,222794.26) | 3378.13(1631.59,5969.12) | -1.91(-1.99,-1.82) | 83831.90(46637.23,139244.18) | 2338.23(1300.80,3883.78) | -1.98(-2.19,-1.77) |
| Ukraine | 14293.88(10685.41,18770.45) | 1742.96(1302.95,2288.82) | -2.98(-3.16,-2.80) | 10579.46(8404.42,12935.84) | 1370.20(1088.50,1675.39) | -3.20(-3.56,-2.83) |
| United Arab Emirates | 2234.80(1267.32,3427.54) | 1009.18(572.29,1547.79) | -3.73(-3.82,-3.63) | 2201.95(1194.22,3103.19) | 1042.44(565.37,1469.10) | -4.06(-4.70,-3.41) |
| United Kingdom | 13566.42(10737.65,17744.13) | 725.65(574.34,949.11) | -2.68(-2.96,-2.41) | 9696.68(8004.40,11331.55) | 543.21(448.41,634.80) | -2.81(-3.17,-2.44) |
| United Republic of Tanzania | 181923.27(92303.80,326753.91) | 4076.32(2068.23,7321.51) | -2.16(-2.49,-1.83) | 124158.79(61985.63,231055.61) | 2832.59(1414.15,5271.35) | -2.25(-2.45,-2.05) |
| United States of America | 72198.83(57404.99,99331.74) | 759.60(603.96,1045.07) | -2.54(-2.89,-2.19) | 56546.87(47358.85,63927.08) | 622.12(521.04,703.32) | -2.76(-2.93,-2.58) |
| United States Virgin Islands | 19.73(10.21,38.78) | 967.85(501.12,1902.83) | -3.77(-4.16,-3.39) | 14.03(8.61,23.63) | 746.17(457.73,1256.26) | -4.08(-4.29,-3.87) |
| Uruguay | 1959.54(1423.23,2571.23) | 1968.49(1429.73,2582.97) | -2.19(-2.49,-1.88) | 1485.79(1066.92,1884.43) | 1573.00(1129.54,1995.04) | -2.33(-2.66,-2.01) |
| Uzbekistan | 102534.76(74204.01,134219.59) | 5139.60(3719.51,6727.81) | 2.30(1.36,3.24) | 87614.59(59807.70,114594.57) | 4761.02(3249.98,6227.13) | 2.25(1.59,2.92) |
| Vanuatu | 565.65(339.74,834.64) | 2595.07(1558.64,3829.18) | -1.70(-2.02,-1.37) | 429.22(246.36,633.07) | 2108.69(1210.33,3110.18) | -1.70(-2.11,-1.29) |
| Venezuela (Bolivarian Republic of) | 37764.32(25264.37,53818.60) | 3380.51(2261.57,4817.63) | 0.18(-0.30,0.66) | 35612.31(24003.84,48063.27) | 3335.06(2247.94,4501.08) | 0.29(0.12,0.47) |
| Viet Nam | 41078.27(23877.51,76225.67) | 970.24(563.97,1800.39) | -2.74(-2.94,-2.53) | 54505.18(22874.67,95312.42) | 1394.73(585.34,2438.94) | -2.87(-3.37,-2.37) |
| Yemen | 237784.44(124299.46,355024.20) | 9864.07(5156.35,14727.56) | -3.13(-3.41,-2.85) | 199588.79(117236.77,282657.31) | 8728.27(5126.91,12360.96) | -3.50(-3.65,-3.35) |
| Zambia | 45796.36(25254.50,80326.43) | 3109.42(1714.70,5453.90) | -3.04(-3.15,-2.92) | 34016.67(20225.63,67475.40) | 2342.41(1392.75,4646.39) | -3.22(-3.57,-2.86) |
| Zimbabwe | 22843.89(13672.23,35709.34) | 2050.28(1227.11,3204.97) | 0.22(-0.81,1.27) | 18131.77(9826.71,31823.66) | 1655.07(896.98,2904.86) | 0.40(0.17,0.63) |

DALYs = disability-adjusted life years; EAPC = estimated annual percentage change.
