# Supplementary material for: Sex differences in global burden of Congenital Heart Anomalies in children under five from 1990 to 2021
Source: PLoS One. 2026 May 6;21(5):e0348351. doi: 10.1371/journal.pone.0348351 (PMC13148693; doi:10.1371/journal.pone.0348351)
Supplement: S8 Table — (DOCX) [file pone.0348351.s008.docx]

**Supplementary Table 8.** National Trends in Deaths Due to Congenital Heart Anomalies among Children Under 5 Years, 2021, and Estimated Annual Percentage Change, 1990–2021.

| location | Male | | | Female | | |
| --- | --- | --- | --- | --- | --- | --- |
|  | 2021 | | EAPC from 1990 to 2021 | 2021 | | EAPC from 1990 to 2021 |
|  | death number  (95% UI) | mortality rate  (95% UI) | rate  (95% CI) | death number  (95% UI) | mortality rate  (95% UI) | rate  (95% CI) |
| Afghanistan | 4396.12(1847.74,6476.95) | 154.27(64.84,227.29) | -2.80(-2.93,-2.67) | 3618.15(1542.37,5606.71) | 137.48(58.61,213.04) | -2.67(-2.75,-2.58) |
| Albania | 22.52(13.09,35.10) | 30.35(17.64,47.31) | -1.94(-2.07,-1.81) | 14.49(7.74,23.86) | 21.18(11.31,34.89) | -1.95(-2.08,-1.83) |
| Algeria | 1141.03(812.10,1625.95) | 47.06(33.49,67.06) | -4.35(-4.59,-4.11) | 847.51(543.12,1189.92) | 37.17(23.82,52.19) | -4.13(-4.35,-3.92) |
| American Samoa | 0.20(0.10,0.36) | 10.48(5.47,19.04) | -1.28(-1.50,-1.06) | 0.31(0.20,0.47) | 17.11(11.07,26.04) | -1.29(-1.46,-1.11) |
| Andorra | 0.02(0.01,0.03) | 1.41(0.81,2.35) | -5.86(-6.18,-5.54) | 0.03(0.02,0.05) | 2.57(1.50,3.95) | -5.70(-6.27,-5.12) |
| Angola | 1024.20(494.82,1810.75) | 36.09(17.44,63.81) | -2.34(-2.66,-2.02) | 677.70(385.12,1108.25) | 24.24(13.78,39.65) | -2.15(-2.55,-1.76) |
| Antigua and Barbuda | 0.43(0.29,0.52) | 15.85(10.69,19.50) | -0.18(-0.37,0.01) | 0.59(0.49,0.70) | 22.94(18.94,27.11) | -0.40(-0.72,-0.09) |
| Argentina | 349.61(265.20,447.37) | 22.84(17.32,29.22) | -1.21(-1.47,-0.96) | 303.79(234.58,383.02) | 20.73(16.01,26.14) | -1.23(-1.35,-1.12) |
| Armenia | 28.88(21.24,37.87) | 29.45(21.65,38.61) | -1.65(-2.22,-1.07) | 18.04(11.84,24.36) | 20.47(13.43,27.64) | -1.56(-1.82,-1.29) |
| Australia | 41.87(27.31,57.17) | 5.42(3.53,7.39) | -3.52(-3.71,-3.32) | 28.11(19.54,36.01) | 3.85(2.68,4.93) | -3.35(-4.01,-2.68) |
| Austria | 14.30(10.28,20.90) | 6.42(4.62,9.38) | -4.94(-5.30,-4.58) | 9.46(7.27,11.32) | 4.52(3.47,5.41) | -4.86(-5.59,-4.12) |
| Azerbaijan | 147.87(86.43,264.98) | 38.55(22.53,69.09) | -1.76(-2.01,-1.50) | 109.74(57.24,181.47) | 32.51(16.96,53.77) | -1.73(-1.98,-1.48) |
| Bahamas | 2.28(1.52,3.13) | 21.27(14.15,29.24) | -1.06(-1.26,-0.87) | 1.34(0.97,1.81) | 12.93(9.37,17.46) | -1.08(-1.30,-0.87) |
| Bahrain | 5.40(3.86,7.42) | 11.27(8.04,15.48) | -4.94(-5.28,-4.59) | 5.33(3.76,7.26) | 11.65(8.21,15.86) | -4.82(-5.07,-4.57) |
| Bangladesh | 1776.96(651.71,3566.86) | 24.10(8.84,48.37) | -4.38(-4.57,-4.18) | 1612.23(770.97,3561.97) | 23.06(11.03,50.95) | -4.22(-4.51,-3.94) |
| Barbados | 1.62(1.06,2.44) | 23.29(15.24,34.95) | -0.93(-1.12,-0.74) | 1.84(1.30,2.57) | 27.77(19.58,38.70) | -0.98(-1.26,-0.69) |
| Belarus | 13.82(7.96,32.54) | 5.74(3.31,13.53) | -5.65(-6.70,-4.60) | 10.21(7.06,15.13) | 4.50(3.11,6.67) | -5.60(-6.02,-5.18) |
| Belgium | 23.13(16.60,30.37) | 7.64(5.48,10.03) | -4.16(-4.45,-3.86) | 14.64(10.85,18.34) | 5.06(3.75,6.34) | -3.76(-4.14,-3.38) |
| Belize | 4.49(3.48,5.75) | 23.29(18.06,29.78) | -1.66(-1.88,-1.43) | 2.77(2.14,3.56) | 14.71(11.36,18.90) | -1.58(-1.76,-1.41) |
| Benin | 635.45(287.27,1040.46) | 53.24(24.07,87.17) | -1.36(-1.48,-1.24) | 559.61(303.76,880.18) | 48.71(26.44,76.62) | -1.46(-1.73,-1.19) |
| Bermuda | 0.14(0.06,0.23) | 10.40(4.48,17.81) | -2.82(-3.02,-2.63) | 0.07(0.03,0.17) | 5.61(2.56,13.68) | -2.68(-3.04,-2.31) |
| Bhutan | 9.88(3.85,18.82) | 31.86(12.42,60.71) | -3.10(-3.39,-2.82) | 8.08(4.05,15.21) | 26.91(13.49,50.64) | -2.95(-3.14,-2.76) |
| Bolivia (Plurinational State of) | 432.28(294.87,597.35) | 70.96(48.40,98.06) | -2.90(-2.99,-2.81) | 311.07(202.06,435.04) | 53.17(34.54,74.36) | -2.71(-2.97,-2.44) |
| Bosnia and Herzegovina | 7.37(5.17,10.14) | 9.46(6.64,13.01) | -4.23(-4.54,-3.92) | 5.71(3.60,9.39) | 7.81(4.93,12.85) | -3.98(-4.21,-3.74) |
| Botswana | 21.76(12.13,34.35) | 18.20(10.15,28.74) | -0.42(-0.48,-0.36) | 13.54(6.88,21.96) | 11.67(5.93,18.94) | -0.57(-0.90,-0.24) |
| Brazil | 2385.29(1859.98,3047.27) | 28.15(21.95,35.97) | -0.54(-0.80,-0.28) | 1848.48(1406.88,2310.42) | 22.86(17.40,28.57) | -0.64(-1.11,-0.16) |
| Brunei Darussalam | 3.20(2.14,4.54) | 19.92(13.33,28.29) | -1.34(-1.68,-1.00) | 3.08(2.11,4.18) | 20.71(14.21,28.15) | -1.35(-1.48,-1.23) |
| Bulgaria | 26.75(19.85,34.23) | 17.19(12.75,22.00) | -2.93(-3.50,-2.36) | 24.67(18.84,30.48) | 16.77(12.81,20.73) | -2.75(-2.85,-2.65) |
| Burkina Faso | 1566.67(532.74,2571.03) | 75.30(25.61,123.58) | -1.14(-1.23,-1.05) | 1458.75(625.40,2486.79) | 72.00(30.87,122.74) | -1.14(-1.27,-1.01) |
| Burundi | 386.08(173.94,693.44) | 35.56(16.02,63.86) | -1.90(-2.20,-1.59) | 289.57(121.64,508.99) | 26.95(11.32,47.37) | -1.90(-2.04,-1.76) |
| Cabo Verde | 2.45(1.19,4.32) | 10.92(5.30,19.24) | -4.49(-4.81,-4.16) | 2.98(1.49,5.58) | 13.80(6.88,25.82) | -4.48(-4.71,-4.26) |
| Cambodia | 669.81(377.75,1033.87) | 74.95(42.27,115.69) | -2.67(-2.88,-2.46) | 496.06(323.69,696.52) | 58.02(37.86,81.47) | -2.58(-2.73,-2.43) |
| Cameroon | 1111.75(425.72,1748.13) | 44.75(17.14,70.37) | -1.23(-1.35,-1.11) | 826.60(448.47,1224.43) | 34.74(18.85,51.46) | -1.26(-1.51,-1.02) |
| Canada | 67.52(51.84,89.41) | 6.93(5.32,9.18) | -3.70(-3.95,-3.45) | 44.86(35.09,55.31) | 4.84(3.79,5.97) | -3.43(-3.56,-3.29) |
| Central African Republic | 282.62(85.05,548.27) | 66.13(19.90,128.29) | -0.89(-1.00,-0.78) | 182.20(72.72,323.40) | 44.28(17.67,78.60) | -0.94(-0.97,-0.90) |
| Chad | 1264.37(287.17,2162.86) | 68.22(15.49,116.70) | -0.59(-0.65,-0.52) | 904.26(370.36,1479.54) | 50.74(20.78,83.02) | -0.70(-1.05,-0.36) |
| Chile | 55.95(44.29,71.07) | 10.09(7.99,12.82) | -2.15(-2.48,-1.82) | 48.66(38.98,58.50) | 9.12(7.30,10.96) | -2.10(-2.31,-1.89) |
| China | 6721.91(4744.46,9878.56) | 16.15(11.40,23.73) | -4.80(-5.23,-4.38) | 5135.80(3859.08,7437.98) | 14.25(10.71,20.64) | -4.73(-5.04,-4.43) |
| Colombia | 532.97(352.77,776.58) | 30.23(20.01,44.04) | -0.34(-0.67,-0.01) | 392.35(260.48,554.91) | 23.36(15.51,33.04) | -0.49(-0.91,-0.08) |
| Comoros | 14.25(6.72,27.91) | 34.29(16.17,67.19) | -2.29(-2.48,-2.11) | 9.72(4.92,19.87) | 24.44(12.37,49.96) | -2.13(-2.33,-1.93) |
| Congo | 73.43(40.97,126.37) | 22.91(12.78,39.42) | -2.36(-2.69,-2.04) | 45.65(28.50,78.22) | 14.62(9.13,25.05) | -2.22(-2.45,-1.99) |
| Cook Islands | 0.03(0.01,0.11) | 5.32(1.15,18.55) | -6.37(-6.90,-5.83) | 0.03(0.01,0.10) | 5.38(2.74,17.75) | -6.14(-6.48,-5.79) |
| Costa Rica | 31.08(23.07,42.43) | 19.77(14.67,26.99) | -1.60(-1.83,-1.36) | 27.40(21.29,33.64) | 18.13(14.09,22.27) | -1.52(-1.62,-1.42) |
| Côte d'Ivoire | 1018.90(444.86,1681.54) | 45.73(19.97,75.47) | -1.25(-1.40,-1.10) | 786.10(461.77,1197.17) | 36.97(21.72,56.30) | -1.27(-1.37,-1.17) |
| Croatia | 8.16(5.61,11.46) | 8.71(5.99,12.22) | -4.47(-4.68,-4.25) | 5.97(4.47,7.78) | 6.74(5.05,8.79) | -4.42(-4.75,-4.08) |
| Cuba | 27.85(20.12,40.63) | 9.89(7.14,14.43) | -4.39(-4.58,-4.20) | 24.35(18.85,30.12) | 9.26(7.17,11.46) | -4.28(-4.53,-4.03) |
| Cyprus | 1.23(0.66,1.90) | 3.19(1.71,4.90) | -7.21(-7.39,-7.03) | 1.55(0.98,2.54) | 4.27(2.69,6.99) | -6.72(-7.01,-6.44) |
| Czechia | 9.15(5.10,14.45) | 3.18(1.77,5.02) | -6.44(-6.75,-6.13) | 5.16(3.23,6.95) | 1.88(1.18,2.53) | -6.38(-6.67,-6.09) |
| Democratic People's Republic of Korea | 183.67(103.14,347.82) | 23.74(13.33,44.96) | -2.99(-3.22,-2.76) | 162.82(98.00,287.63) | 22.02(13.25,38.90) | -2.88(-3.22,-2.54) |
| Democratic Republic of the Congo | 1832.35(919.77,3411.95) | 26.54(13.32,49.42) | -2.23(-2.51,-1.95) | 1526.46(652.12,2824.48) | 22.93(9.80,42.43) | -2.11(-2.42,-1.81) |
| Denmark | 12.38(8.34,15.89) | 7.77(5.23,9.97) | -5.21(-5.67,-4.76) | 6.59(4.62,8.07) | 4.36(3.05,5.33) | -5.13(-5.48,-4.77) |
| Djibouti | 19.72(8.94,38.06) | 25.68(11.64,49.56) | -1.87(-2.22,-1.51) | 13.25(5.98,26.22) | 19.23(8.68,38.06) | -1.86(-2.01,-1.71) |
| Dominica | 0.62(0.38,0.97) | 35.25(21.52,54.66) | 1.25(0.93,1.56) | 0.73(0.40,1.12) | 42.46(23.35,65.58) | 1.12(0.42,1.82) |
| Dominican Republic | 121.36(53.95,259.25) | 23.00(10.23,49.14) | -2.85(-3.04,-2.66) | 96.62(51.13,203.27) | 19.11(10.11,40.21) | -2.69(-2.85,-2.54) |
| Ecuador | 291.60(214.64,386.39) | 34.43(25.34,45.62) | -0.75(-0.99,-0.51) | 236.75(169.92,317.79) | 29.08(20.87,39.03) | -0.76(-0.89,-0.64) |
| Egypt | 2573.64(1816.42,3602.13) | 38.49(27.17,53.88) | -4.83(-5.01,-4.66) | 2166.28(1470.28,3005.94) | 34.11(23.15,47.32) | -4.74(-4.95,-4.52) |
| El Salvador | 76.83(40.37,132.59) | 24.74(13.00,42.69) | -4.42(-4.60,-4.25) | 71.58(46.37,106.31) | 24.67(15.98,36.64) | -4.30(-4.81,-3.78) |
| Equatorial Guinea | 23.87(12.49,42.35) | 24.07(12.59,42.70) | -3.22(-3.47,-2.97) | 12.08(6.43,20.88) | 13.70(7.30,23.68) | -3.18(-3.33,-3.03) |
| Eritrea | 178.12(87.42,344.50) | 37.61(18.46,72.74) | -1.81(-1.91,-1.71) | 125.10(55.54,258.94) | 28.15(12.50,58.26) | -1.78(-1.91,-1.65) |
| Estonia | 1.36(0.69,2.45) | 3.82(1.94,6.88) | -6.29(-6.87,-5.69) | 0.97(0.62,1.21) | 2.88(1.84,3.59) | -5.95(-6.32,-5.58) |
| Eswatini | 13.39(7.75,23.31) | 18.62(10.77,32.41) | -1.06(-1.32,-0.80) | 10.71(6.11,17.27) | 15.64(8.92,25.21) | -1.11(-1.39,-0.84) |
| Ethiopia | 2818.99(1294.59,5432.07) | 34.49(15.84,66.45) | -3.19(-3.34,-3.04) | 2140.35(1168.71,3964.05) | 27.47(15.00,50.88) | -3.03(-3.18,-2.87) |
| Fiji | 24.17(16.05,35.39) | 51.38(34.13,75.23) | 0.00(-0.22,0.22) | 16.04(11.18,23.02) | 36.45(25.41,52.31) | -0.06(-0.33,0.20) |
| Finland | 6.70(3.68,10.44) | 5.39(2.96,8.40) | -4.88(-5.04,-4.73) | 4.90(3.34,6.05) | 4.13(2.82,5.11) | -4.76(-4.98,-4.53) |
| France | 93.54(64.32,145.75) | 5.18(3.57,8.08) | -5.02(-5.20,-4.84) | 80.60(60.85,101.14) | 4.66(3.52,5.85) | -4.87(-5.45,-4.28) |
| Gabon | 23.01(13.28,42.31) | 21.39(12.34,39.32) | -1.62(-1.90,-1.34) | 11.63(6.36,19.92) | 10.98(6.00,18.81) | -1.55(-1.82,-1.29) |
| Gambia | 46.67(28.01,72.93) | 25.63(15.39,40.06) | -2.04(-2.30,-1.78) | 45.59(27.50,68.42) | 25.97(15.67,38.98) | -1.98(-2.10,-1.86) |
| Georgia | 27.34(19.53,35.91) | 21.67(15.48,28.46) | -1.24(-1.84,-0.62) | 19.29(10.82,25.84) | 16.47(9.23,22.06) | -1.27(-1.40,-1.13) |
| Germany | 137.29(97.96,179.74) | 6.62(4.72,8.66) | -4.32(-4.50,-4.13) | 114.36(86.76,135.70) | 5.81(4.41,6.89) | -4.07(-4.31,-3.84) |
| Ghana | 613.92(324.29,945.56) | 25.87(13.67,39.85) | -1.73(-1.87,-1.60) | 508.30(288.78,778.63) | 22.42(12.74,34.34) | -1.70(-1.94,-1.46) |
| Greece | 22.18(16.12,29.67) | 10.23(7.44,13.69) | -4.21(-4.53,-3.90) | 17.10(13.72,20.89) | 8.31(6.66,10.15) | -3.94(-4.20,-3.68) |
| Greenland | 0.15(0.07,0.35) | 7.18(3.52,16.69) | -5.35(-5.52,-5.19) | 0.15(0.09,0.28) | 7.79(4.72,14.69) | -5.33(-5.60,-5.05) |
| Grenada | 1.02(0.75,1.33) | 29.01(21.43,37.84) | -0.97(-1.17,-0.77) | 0.95(0.73,1.20) | 28.35(21.85,35.84) | -1.07(-1.16,-0.98) |
| Guam | 1.15(0.73,1.62) | 17.36(10.97,24.38) | -0.12(-0.58,0.33) | 0.78(0.51,1.39) | 12.62(8.31,22.59) | -0.22(-0.48,0.04) |
| Guatemala | 308.34(206.30,427.20) | 39.01(26.10,54.05) | 2.12(1.35,2.89) | 214.31(153.29,283.56) | 27.87(19.94,36.88) | 1.57(0.79,2.36) |
| Guinea | 717.52(283.37,1109.11) | 62.45(24.66,96.53) | -1.80(-1.91,-1.69) | 602.05(307.11,948.33) | 54.66(27.88,86.10) | -1.74(-1.84,-1.63) |
| Guinea-Bissau | 63.34(31.80,98.82) | 37.36(18.76,58.30) | -2.66(-2.96,-2.36) | 56.70(30.83,93.50) | 34.76(18.90,57.31) | -2.51(-2.73,-2.29) |
| Guyana | 12.04(7.85,17.30) | 31.51(20.55,45.31) | -0.16(-0.36,0.04) | 9.48(6.95,12.68) | 26.10(19.12,34.90) | -0.38(-0.61,-0.14) |
| Haiti | 1005.86(580.37,1598.43) | 126.39(72.92,200.84) | -2.23(-2.45,-2.02) | 753.06(338.24,1430.35) | 97.30(43.70,184.81) | -2.12(-2.75,-1.48) |
| Honduras | 175.01(106.81,276.89) | 31.30(19.10,49.51) | -3.03(-3.12,-2.93) | 105.83(61.72,198.18) | 19.73(11.51,36.95) | -2.89(-3.40,-2.38) |
| Hungary | 20.04(13.30,27.97) | 8.60(5.71,12.00) | -4.50(-4.73,-4.27) | 13.74(9.32,18.10) | 6.21(4.21,8.18) | -4.56(-5.01,-4.11) |
| Iceland | 0.65(0.39,0.99) | 5.79(3.49,8.74) | -4.38(-4.66,-4.09) | 0.48(0.35,0.61) | 4.51(3.29,5.69) | -4.18(-4.49,-3.87) |
| India | 19006.45(12677.45,29635.55) | 32.58(21.73,50.79) | -2.41(-2.51,-2.31) | 13872.94(9253.10,21381.67) | 26.18(17.46,40.35) | -2.23(-2.38,-2.08) |
| Indonesia | 4240.74(2925.70,5821.19) | 37.76(26.05,51.83) | -2.08(-2.22,-1.94) | 2304.59(1335.48,3333.02) | 21.59(12.51,31.22) | -2.10(-2.23,-1.96) |
| Iran (Islamic Republic of) | 321.09(194.73,518.78) | 10.14(6.15,16.39) | -5.76(-6.44,-5.08) | 294.94(204.17,457.23) | 9.87(6.83,15.30) | -5.60(-6.02,-5.19) |
| Iraq | 977.08(621.80,1525.50) | 44.18(28.11,68.97) | -3.40(-3.65,-3.16) | 686.33(486.48,979.71) | 32.97(23.37,47.06) | -3.34(-3.68,-2.99) |
| Ireland | 8.65(6.04,12.82) | 5.65(3.95,8.38) | -3.83(-4.10,-3.57) | 6.86(5.27,8.66) | 4.71(3.63,5.95) | -3.46(-3.65,-3.27) |
| Israel | 28.14(20.12,40.40) | 5.97(4.27,8.57) | -4.31(-4.49,-4.13) | 18.85(13.70,24.28) | 4.22(3.07,5.43) | -4.01(-4.25,-3.77) |
| Italy | 77.63(55.37,100.43) | 6.96(4.97,9.01) | -4.53(-4.72,-4.35) | 51.81(36.58,64.61) | 4.91(3.47,6.12) | -4.62(-4.96,-4.28) |
| Jamaica | 18.93(13.01,26.80) | 21.75(14.96,30.80) | -1.15(-1.39,-0.91) | 15.91(11.53,21.23) | 18.90(13.70,25.22) | -1.16(-1.65,-0.67) |
| Japan | 136.17(81.41,202.74) | 5.79(3.46,8.62) | -4.08(-4.28,-3.89) | 96.20(70.16,119.75) | 4.31(3.14,5.36) | -3.72(-4.20,-3.23) |
| Jordan | 182.67(126.95,266.02) | 32.37(22.49,47.14) | -3.69(-3.80,-3.58) | 142.86(106.59,190.33) | 26.81(20.00,35.71) | -3.41(-3.76,-3.06) |
| Kazakhstan | 338.70(251.52,447.68) | 33.72(25.04,44.57) | 0.64(-0.17,1.46) | 245.45(157.96,312.80) | 26.00(16.73,33.13) | 0.76(0.54,0.97) |
| Kenya | 541.65(290.18,1013.36) | 17.91(9.60,33.51) | -1.90(-2.06,-1.74) | 317.51(150.92,836.43) | 10.85(5.16,28.59) | -1.92(-2.16,-1.67) |
| Kiribati | 3.90(1.17,6.09) | 52.57(15.72,82.23) | -1.88(-1.96,-1.80) | 2.60(1.08,4.25) | 37.79(15.71,61.70) | -1.88(-2.19,-1.57) |
| Kuwait | 30.67(23.77,40.08) | 22.54(17.46,29.45) | -3.21(-3.57,-2.84) | 21.03(16.78,26.60) | 16.31(13.01,20.64) | -3.12(-3.50,-2.74) |
| Kyrgyzstan | 139.27(108.01,176.19) | 34.20(26.52,43.27) | -0.21(-0.56,0.15) | 110.93(76.74,139.47) | 28.69(19.85,36.07) | -0.46(-0.80,-0.12) |
| Lao People's Democratic Republic | 383.60(176.43,629.75) | 90.67(41.70,148.85) | -2.44(-2.62,-2.26) | 304.92(160.28,468.87) | 75.02(39.43,115.36) | -2.25(-2.43,-2.07) |
| Latvia | 2.64(1.77,4.64) | 5.44(3.66,9.58) | -5.21(-5.86,-4.55) | 2.37(1.86,2.92) | 5.24(4.11,6.44) | -5.11(-5.43,-4.79) |
| Lebanon | 26.09(15.55,39.39) | 12.35(7.37,18.66) | -4.50(-4.74,-4.27) | 23.67(14.60,39.64) | 12.16(7.50,20.35) | -4.57(-4.69,-4.45) |
| Lesotho | 30.00(14.31,52.96) | 29.19(13.93,51.54) | -0.55(-0.65,-0.45) | 16.31(8.37,27.27) | 16.15(8.29,27.01) | -0.64(-0.96,-0.32) |
| Liberia | 167.25(69.90,271.50) | 42.62(17.81,69.19) | -3.21(-3.51,-2.92) | 139.30(76.13,225.30) | 37.25(20.36,60.24) | -3.12(-3.25,-3.00) |
| Libya | 152.20(77.47,232.04) | 70.30(35.78,107.17) | -2.64(-3.15,-2.13) | 84.29(40.73,134.60) | 40.81(19.72,65.17) | -2.45(-2.65,-2.25) |
| Lithuania | 6.72(4.50,10.02) | 9.95(6.67,14.84) | -4.32(-4.65,-3.99) | 3.62(2.59,4.45) | 5.65(4.04,6.95) | -4.12(-4.50,-3.74) |
| Luxembourg | 0.59(0.35,1.08) | 3.51(2.05,6.42) | -5.36(-5.83,-4.88) | 0.40(0.30,0.51) | 2.47(1.86,3.13) | -5.45(-5.76,-5.14) |
| Madagascar | 754.51(340.67,1429.76) | 36.34(16.41,68.86) | -1.81(-1.94,-1.67) | 451.00(201.23,936.68) | 22.42(10.00,46.56) | -1.74(-1.86,-1.62) |
| Malawi | 495.60(264.39,901.95) | 36.12(19.27,65.74) | -3.33(-3.47,-3.18) | 316.40(161.12,611.73) | 23.40(11.92,45.25) | -3.33(-3.65,-3.02) |
| Malaysia | 148.99(95.10,215.78) | 11.75(7.50,17.01) | -3.31(-3.84,-2.78) | 125.79(89.24,182.17) | 10.57(7.50,15.31) | -3.30(-3.49,-3.11) |
| Maldives | 3.10(1.96,4.67) | 19.00(12.01,28.62) | -4.61(-4.78,-4.45) | 3.14(2.20,4.66) | 20.30(14.20,30.18) | -4.69(-4.92,-4.46) |
| Mali | 1517.43(841.11,2268.33) | 65.08(36.08,97.29) | -2.46(-2.60,-2.32) | 1532.44(762.10,2416.46) | 68.14(33.89,107.45) | -2.38(-2.50,-2.26) |
| Malta | 1.18(0.84,1.65) | 10.34(7.39,14.48) | -2.94(-3.30,-2.58) | 1.15(0.89,1.44) | 10.80(8.41,13.55) | -2.81(-3.13,-2.48) |
| Marshall Islands | 0.87(0.51,1.38) | 29.65(17.33,47.02) | -0.56(-0.85,-0.26) | 0.58(0.36,0.88) | 21.11(12.97,32.03) | -0.68(-0.85,-0.50) |
| Mauritania | 77.09(43.14,122.07) | 23.01(12.88,36.44) | -1.99(-2.34,-1.63) | 61.31(35.27,91.02) | 19.04(10.95,28.27) | -1.97(-2.08,-1.86) |
| Mauritius | 9.91(7.38,14.00) | 30.34(22.58,42.85) | -1.18(-1.39,-0.97) | 7.84(6.06,10.67) | 24.79(19.16,33.75) | -1.22(-1.41,-1.02) |
| Mexico | 2195.56(1553.87,2962.22) | 43.93(31.09,59.27) | 0.31(0.05,0.56) | 1702.78(1227.53,2193.74) | 34.90(25.16,44.96) | 0.37(-0.24,0.98) |
| Micronesia (Federated States of) | 1.06(0.71,1.59) | 21.63(14.60,32.40) | -2.93(-3.01,-2.85) | 0.78(0.50,1.19) | 17.13(10.83,26.02) | -2.75(-2.87,-2.63) |
| Monaco | 0.05(0.03,0.09) | 6.19(3.27,11.32) | -5.27(-5.66,-4.87) | 0.07(0.05,0.11) | 9.32(6.15,14.25) | -5.19(-5.42,-4.96) |
| Mongolia | 46.61(26.12,70.44) | 23.22(13.01,35.09) | -3.74(-3.89,-3.59) | 45.96(30.52,65.74) | 24.19(16.07,34.61) | -3.43(-3.59,-3.27) |
| Montenegro | 0.61(0.27,1.26) | 3.22(1.42,6.73) | -5.35(-5.76,-4.94) | 0.73(0.45,1.39) | 4.18(2.56,7.98) | -5.26(-5.40,-5.11) |
| Morocco | 300.13(165.09,645.14) | 18.02(9.91,38.74) | -4.01(-4.29,-3.72) | 225.28(115.35,558.99) | 14.21(7.27,35.25) | -3.60(-3.77,-3.42) |
| Mozambique | 1226.23(532.20,2376.45) | 46.95(20.38,90.99) | -2.66(-2.81,-2.51) | 900.97(419.98,1680.84) | 35.09(16.36,65.47) | -2.54(-2.79,-2.29) |
| Myanmar | 2678.89(1226.83,4188.48) | 100.24(45.91,156.73) | -1.81(-2.07,-1.55) | 1824.88(1000.29,2707.24) | 71.45(39.17,106.00) | -1.77(-2.03,-1.51) |
| Namibia | 21.04(11.40,36.08) | 14.98(8.12,25.69) | -1.42(-1.59,-1.24) | 16.73(10.08,27.31) | 12.12(7.30,19.78) | -1.48(-1.70,-1.27) |
| Nauru | 0.30(0.16,0.48) | 41.05(21.82,65.28) | -0.12(-0.55,0.30) | 0.21(0.12,0.30) | 30.88(17.29,44.99) | -0.28(-0.62,0.05) |
| Nepal | 222.76(98.50,593.27) | 13.89(6.14,36.99) | -4.06(-4.15,-3.98) | 249.57(126.15,595.94) | 16.61(8.40,39.67) | -3.64(-3.81,-3.48) |
| Netherlands | 26.05(20.23,37.23) | 5.91(4.59,8.44) | -4.58(-4.83,-4.33) | 17.83(14.59,21.27) | 4.24(3.47,5.06) | -4.65(-5.14,-4.15) |
| New Zealand | 8.38(5.15,13.41) | 5.22(3.21,8.36) | -3.94(-4.35,-3.53) | 7.30(5.76,8.71) | 4.80(3.79,5.73) | -3.53(-3.59,-3.48) |
| Nicaragua | 84.78(50.54,140.09) | 25.40(15.14,41.96) | -4.11(-4.26,-3.96) | 69.09(45.36,101.01) | 21.82(14.32,31.89) | -3.73(-4.11,-3.35) |
| Niger | 1178.97(372.25,1894.94) | 45.37(14.33,72.93) | -2.30(-2.50,-2.11) | 1257.63(498.86,2078.09) | 50.35(19.97,83.19) | -2.13(-2.36,-1.90) |
| Nigeria | 10971.10(4025.18,17535.07) | 58.59(21.50,93.65) | -0.84(-0.94,-0.74) | 8962.63(4281.80,14884.09) | 48.73(23.28,80.92) | -0.90(-1.03,-0.78) |
| Niue | 0.06(0.04,0.08) | 94.73(66.30,131.22) | 0.75(0.25,1.25) | 0.05(0.03,0.07) | 84.27(60.75,122.28) | 0.92(-0.02,1.86) |
| North Macedonia | 4.56(2.66,8.08) | 8.77(5.13,15.55) | -6.08(-6.45,-5.72) | 3.39(2.27,5.53) | 6.98(4.67,11.39) | -5.91(-6.05,-5.78) |
| Northern Mariana Islands | 0.12(0.08,0.19) | 7.43(4.85,11.45) | -1.48(-1.76,-1.19) | 0.11(0.07,0.18) | 7.31(4.60,11.65) | -1.50(-1.66,-1.34) |
| Norway | 6.20(3.51,12.28) | 4.29(2.43,8.50) | -5.36(-5.61,-5.10) | 4.02(2.66,5.50) | 2.94(1.94,4.02) | -5.38(-5.61,-5.14) |
| Oman | 37.23(24.53,54.48) | 17.21(11.34,25.18) | -5.07(-5.80,-4.34) | 34.14(24.07,49.75) | 16.44(11.59,23.95) | -4.93(-5.38,-4.49) |
| Pakistan | 5409.78(3048.61,8162.41) | 35.34(19.91,53.32) | -1.42(-1.62,-1.23) | 4774.68(2504.89,7608.50) | 33.12(17.37,52.77) | -1.49(-1.68,-1.31) |
| Palau | 0.13(0.09,0.19) | 27.14(17.70,39.23) | -2.08(-2.22,-1.93) | 0.08(0.05,0.11) | 16.79(10.06,24.23) | -2.05(-2.17,-1.94) |
| Palestine | 81.13(52.44,119.91) | 25.82(16.69,38.16) | -4.12(-4.31,-3.93) | 71.24(49.54,103.69) | 23.86(16.59,34.73) | -3.73(-4.18,-3.28) |
| Panama | 88.91(63.22,116.96) | 46.59(33.13,61.29) | -0.83(-0.93,-0.73) | 69.67(52.78,88.28) | 38.62(29.26,48.94) | -0.81(-1.11,-0.51) |
| Papua New Guinea | 791.11(197.18,1303.18) | 99.52(24.80,163.93) | -0.32(-0.43,-0.22) | 562.15(199.77,927.65) | 77.39(27.50,127.71) | -0.49(-0.72,-0.26) |
| Paraguay | 101.89(55.56,167.17) | 30.52(16.64,50.07) | -1.34(-1.53,-1.15) | 81.48(51.31,125.06) | 25.77(16.23,39.55) | -1.32(-1.76,-0.87) |
| Peru | 407.33(225.57,641.18) | 23.92(13.25,37.66) | -4.34(-4.59,-4.08) | 357.46(210.09,513.95) | 22.37(13.15,32.17) | -4.12(-4.30,-3.94) |
| Philippines | 2033.45(1493.26,3042.76) | 34.91(25.64,52.24) | -2.34(-2.46,-2.21) | 1550.76(1200.75,2069.86) | 28.78(22.28,38.41) | -2.14(-2.33,-1.94) |
| Poland | 120.34(85.95,161.73) | 12.45(8.89,16.74) | -4.48(-4.67,-4.28) | 92.72(67.51,115.92) | 10.12(7.37,12.66) | -4.42(-4.80,-4.04) |
| Portugal | 14.45(9.78,19.33) | 6.66(4.50,8.90) | -5.54(-5.87,-5.21) | 9.93(7.37,12.46) | 4.77(3.54,5.98) | -5.48(-6.47,-4.48) |
| Puerto Rico | 8.47(6.30,10.81) | 15.71(11.69,20.05) | -1.83(-2.07,-1.59) | 5.41(3.73,6.80) | 10.57(7.28,13.29) | -1.80(-1.91,-1.70) |
| Qatar | 8.03(4.67,12.47) | 8.58(5.00,13.33) | -5.10(-5.22,-4.97) | 8.35(5.77,11.76) | 9.20(6.36,12.95) | -5.01(-5.26,-4.76) |
| Republic of Korea | 34.33(20.90,51.42) | 4.34(2.64,6.49) | -6.46(-6.59,-6.33) | 35.20(23.28,62.03) | 4.64(3.07,8.18) | -6.44(-7.04,-5.83) |
| Republic of Moldova | 20.14(14.18,27.97) | 25.37(17.86,35.23) | -2.32(-2.58,-2.05) | 13.95(10.31,19.18) | 18.65(13.78,25.64) | -2.13(-2.21,-2.05) |
| Romania | 85.29(66.52,102.44) | 17.72(13.82,21.28) | -3.29(-3.73,-2.83) | 64.74(52.23,77.27) | 14.18(11.44,16.93) | -3.25(-3.58,-2.93) |
| Russian Federation | 407.39(274.11,602.47) | 10.42(7.01,15.41) | -3.10(-3.76,-2.45) | 293.37(217.54,338.84) | 7.92(5.88,9.15) | -2.95(-3.29,-2.60) |
| Rwanda | 278.74(152.09,502.93) | 31.38(17.12,56.61) | -2.50(-2.89,-2.11) | 215.34(108.25,405.81) | 25.04(12.59,47.18) | -2.44(-2.69,-2.20) |
| Saint Kitts and Nevis | 0.33(0.23,0.46) | 21.47(15.08,29.56) | -1.50(-1.77,-1.23) | 0.37(0.27,0.48) | 24.36(17.98,32.21) | -1.51(-1.77,-1.24) |
| Saint Lucia | 1.16(0.78,1.68) | 25.73(17.34,37.12) | -1.35(-1.60,-1.09) | 0.59(0.41,0.82) | 13.55(9.58,18.99) | -1.42(-1.73,-1.11) |
| Saint Vincent and the Grenadines | 0.73(0.51,1.03) | 20.02(14.08,28.09) | -2.33(-2.58,-2.07) | 0.50(0.37,0.67) | 14.22(10.40,18.80) | -2.14(-2.28,-1.99) |
| Samoa | 2.94(1.79,4.56) | 19.36(11.82,30.02) | -2.78(-2.90,-2.66) | 2.58(1.59,4.10) | 18.32(11.34,29.13) | -2.65(-2.84,-2.46) |
| San Marino | 0.01(0.00,0.02) | 1.35(0.54,3.10) | -5.92(-6.21,-5.62) | 0.01(0.00,0.02) | 1.36(0.63,3.15) | -5.80(-5.90,-5.69) |
| Sao Tome and Principe | 1.66(0.92,3.09) | 13.06(7.22,24.24) | -3.96(-4.38,-3.55) | 2.21(1.07,4.92) | 18.13(8.76,40.29) | -3.56(-3.65,-3.47) |
| Saudi Arabia | 95.07(39.18,199.25) | 7.57(3.12,15.86) | -7.84(-8.03,-7.65) | 91.65(49.57,185.25) | 7.79(4.21,15.74) | -6.90(-7.35,-6.44) |
| Senegal | 364.87(222.53,567.83) | 31.37(19.13,48.82) | -1.87(-2.17,-1.57) | 341.90(207.21,531.46) | 30.85(18.70,47.95) | -1.86(-2.10,-1.63) |
| Serbia | 20.11(12.57,28.18) | 10.53(6.59,14.76) | -7.31(-7.88,-6.74) | 14.29(9.92,25.27) | 8.04(5.58,14.21) | -6.79(-6.96,-6.61) |
| Seychelles | 1.33(0.94,1.84) | 33.18(23.31,45.82) | -0.67(-1.08,-0.26) | 1.21(0.79,1.69) | 31.50(20.43,43.86) | -0.74(-0.83,-0.66) |
| Sierra Leone | 502.61(167.36,846.83) | 73.96(24.63,124.62) | -1.91(-2.07,-1.75) | 388.94(197.76,621.91) | 58.63(29.81,93.74) | -1.92(-2.28,-1.57) |
| Singapore | 5.62(3.11,9.92) | 3.84(2.12,6.79) | -7.00(-7.42,-6.59) | 4.57(3.04,6.01) | 3.27(2.18,4.30) | -6.48(-6.61,-6.34) |
| Slovakia | 19.01(13.47,25.08) | 12.96(9.18,17.09) | -3.56(-3.77,-3.34) | 13.22(9.83,18.83) | 9.49(7.05,13.51) | -3.37(-3.58,-3.16) |
| Slovenia | 2.15(1.31,3.24) | 4.27(2.61,6.42) | -6.09(-6.27,-5.91) | 1.40(0.94,1.74) | 2.95(1.97,3.66) | -5.94(-6.34,-5.54) |
| Solomon Islands | 16.06(10.19,24.23) | 32.29(20.48,48.73) | -1.92(-2.00,-1.84) | 14.07(7.81,21.93) | 30.85(17.13,48.10) | -1.95(-2.16,-1.75) |
| Somalia | 1033.34(273.56,2301.14) | 48.34(12.80,107.65) | -1.13(-1.32,-0.94) | 732.72(236.29,1702.58) | 36.79(11.86,85.49) | -1.12(-1.42,-0.81) |
| South Africa | 401.70(220.95,650.31) | 15.98(8.79,25.88) | -1.75(-1.90,-1.59) | 349.76(222.23,501.95) | 14.29(9.08,20.50) | -1.70(-1.83,-1.57) |
| South Sudan | 734.38(139.59,1462.37) | 90.97(17.29,181.14) | -0.76(-1.05,-0.47) | 434.75(154.27,862.31) | 57.58(20.43,114.21) | -0.81(-1.06,-0.55) |
| Spain | 66.35(45.63,84.97) | 7.01(4.82,8.98) | -4.93(-5.25,-4.61) | 37.58(27.14,46.41) | 4.20(3.03,5.19) | -4.81(-5.00,-4.62) |
| Sri Lanka | 145.83(90.80,231.53) | 18.29(11.38,29.03) | -2.70(-3.11,-2.30) | 121.07(84.78,179.58) | 15.76(11.04,23.38) | -2.59(-2.72,-2.46) |
| Sudan | 3230.68(1782.30,4942.94) | 110.98(61.22,169.80) | -3.10(-3.29,-2.90) | 2020.98(1172.27,3029.03) | 74.08(42.97,111.04) | -2.91(-3.02,-2.80) |
| Suriname | 7.46(4.41,11.71) | 32.79(19.37,51.48) | -1.95(-2.03,-1.88) | 5.34(3.12,7.89) | 24.51(14.30,36.18) | -1.97(-3.08,-0.84) |
| Sweden | 12.96(8.04,20.43) | 4.33(2.68,6.82) | -5.34(-5.71,-4.98) | 5.51(3.90,9.86) | 1.94(1.38,3.48) | -5.23(-5.33,-5.14) |
| Switzerland | 19.31(14.37,24.75) | 8.51(6.33,10.90) | -4.52(-4.83,-4.22) | 12.19(9.61,14.67) | 5.67(4.47,6.83) | -4.58(-4.77,-4.39) |
| Syrian Arab Republic | 142.90(89.42,225.22) | 27.71(17.34,43.68) | -4.36(-4.86,-3.85) | 137.63(86.53,199.30) | 28.12(17.68,40.72) | -4.16(-4.25,-4.06) |
| Taiwan (Province of China) | 42.83(31.80,54.98) | 9.28(6.89,11.91) | -3.15(-3.47,-2.83) | 32.65(26.59,38.56) | 7.58(6.17,8.95) | -2.99(-3.29,-2.69) |
| Tajikistan | 200.66(99.82,495.80) | 29.03(14.44,71.72) | -0.00(-0.17,0.17) | 153.85(79.14,313.73) | 23.77(12.23,48.47) | -0.17(-0.62,0.27) |
| Thailand | 226.47(118.06,316.45) | 15.58(8.12,21.77) | -3.90(-4.04,-3.76) | 213.84(145.46,289.67) | 15.57(10.59,21.10) | -3.48(-3.53,-3.42) |
| Timor-Leste | 62.40(34.37,96.15) | 65.52(36.09,100.95) | -2.96(-3.09,-2.83) | 52.58(33.09,77.47) | 58.68(36.93,86.45) | -2.87(-3.02,-2.71) |
| Togo | 205.19(99.77,326.32) | 34.44(16.75,54.77) | -1.72(-1.88,-1.55) | 156.17(90.65,252.82) | 27.08(15.72,43.84) | -1.67(-1.93,-1.41) |
| Tokelau | 0.05(0.03,0.08) | 95.61(57.09,160.43) | -2.44(-3.47,-1.40) | 0.05(0.03,0.09) | 103.08(68.54,182.54) | -2.28(-2.39,-2.16) |
| Tonga | 1.29(0.83,1.94) | 17.19(11.07,25.78) | -1.72(-1.88,-1.57) | 0.54(0.33,0.99) | 7.88(4.80,14.42) | -1.68(-1.80,-1.56) |
| Trinidad and Tobago | 15.32(10.49,21.22) | 37.25(25.51,51.60) | 0.04(-0.24,0.32) | 12.39(9.20,16.55) | 31.47(23.36,42.03) | -0.06(-0.50,0.39) |
| Tunisia | 132.82(91.94,189.62) | 28.64(19.82,40.88) | -4.76(-4.89,-4.63) | 88.98(58.38,124.87) | 20.78(13.63,29.16) | -4.71(-5.05,-4.37) |
| Türkiye | 879.28(607.64,1211.93) | 30.84(21.31,42.50) | -5.89(-6.07,-5.71) | 706.16(489.15,942.56) | 26.15(18.11,34.90) | -5.75(-6.04,-5.47) |
| Turkmenistan | 182.63(127.68,240.05) | 65.98(46.13,86.72) | 1.94(1.31,2.57) | 109.49(71.00,160.01) | 41.64(27.01,60.86) | 1.42(0.82,2.02) |
| Tuvalu | 0.22(0.13,0.34) | 32.01(19.06,50.14) | -4.43(-4.59,-4.28) | 0.11(0.07,0.17) | 18.09(11.28,28.55) | -4.38(-4.65,-4.10) |
| Uganda | 1395.34(663.28,2481.58) | 37.38(17.77,66.49) | -1.72(-1.86,-1.57) | 921.06(503.51,1539.29) | 25.69(14.04,42.93) | -1.68(-1.82,-1.54) |
| Ukraine | 151.81(112.20,200.91) | 18.51(13.68,24.50) | -2.92(-3.16,-2.67) | 112.23(88.30,138.80) | 14.54(11.44,17.98) | -2.73(-2.83,-2.62) |
| United Arab Emirates | 23.36(12.33,36.55) | 10.55(5.57,16.51) | -4.91(-5.34,-4.47) | 23.12(11.90,33.28) | 10.95(5.64,15.75) | -4.78(-5.02,-4.54) |
| United Kingdom | 139.42(107.61,186.43) | 7.46(5.76,9.97) | -4.14(-4.53,-3.76) | 98.99(81.75,118.47) | 5.55(4.58,6.64) | -3.74(-3.96,-3.53) |
| United Republic of Tanzania | 2019.84(1016.50,3643.16) | 45.26(22.78,81.63) | -2.08(-2.21,-1.94) | 1370.94(673.37,2569.31) | 31.28(15.36,58.62) | -1.99(-2.22,-1.76) |
| United States of America | 741.73(579.94,1063.32) | 7.80(6.10,11.19) | -3.21(-3.34,-3.07) | 580.60(479.21,660.91) | 6.39(5.27,7.27) | -3.12(-3.55,-2.69) |
| United States Virgin Islands | 0.21(0.10,0.42) | 10.11(4.98,20.54) | -4.31(-4.45,-4.17) | 0.15(0.09,0.25) | 7.76(4.56,13.44) | -3.99(-4.31,-3.66) |
| Uruguay | 21.08(15.15,27.88) | 21.18(15.22,28.01) | -2.03(-2.26,-1.80) | 16.02(11.38,20.42) | 16.96(12.05,21.61) | -1.98(-2.09,-1.87) |
| Uzbekistan | 1129.36(811.65,1488.43) | 56.61(40.68,74.61) | 0.60(-0.03,1.23) | 965.40(655.17,1268.47) | 52.46(35.60,68.93) | 0.50(0.32,0.69) |
| Vanuatu | 6.20(3.65,9.19) | 28.42(16.75,42.16) | -1.59(-1.85,-1.33) | 4.71(2.66,6.98) | 23.12(13.08,34.31) | -1.51(-1.78,-1.25) |
| Venezuela (Bolivarian Republic of) | 413.89(273.45,593.70) | 37.05(24.48,53.15) | 0.25(0.14,0.36) | 391.54(262.55,530.64) | 36.67(24.59,49.69) | 0.27(0.09,0.44) |
| Viet Nam | 437.18(244.91,825.71) | 10.33(5.78,19.50) | -2.37(-2.71,-2.03) | 588.61(237.44,1047.18) | 15.06(6.08,26.80) | -2.23(-2.47,-1.98) |
| Yemen | 2636.96(1375.47,3948.68) | 109.39(57.06,163.80) | -3.11(-3.25,-2.97) | 2216.92(1294.20,3149.92) | 96.95(56.60,137.75) | -2.98(-3.27,-2.69) |
| Zambia | 507.51(277.57,893.58) | 34.46(18.85,60.67) | -2.34(-2.69,-2.00) | 374.42(218.69,749.33) | 25.78(15.06,51.60) | -2.16(-2.32,-2.01) |
| Zimbabwe | 250.34(147.74,395.12) |  | -0.20(-0.45,0.05) | 196.31(103.94,348.56) |  | -0.45(-0.60,-0.30) |

DALYs = disability-adjusted life years; EAPC = estimated annual percentage change.
