# Supplementary material for: Sex differences in global burden of Congenital Heart Anomalies in children under five from 1990 to 2021
Source: PLoS One. 2026 May 6;21(5):e0348351. doi: 10.1371/journal.pone.0348351 (PMC13148693; doi:10.1371/journal.pone.0348351)
Supplement: S9 Table — (DOCX) [file pone.0348351.s009.docx]

**Supplementary Table 9.** National Trends in Incidence of Congenital Heart Anomalies among Children Under 5 Years, 2021, and Estimated Annual Percentage Change, 1990–2021

| location | Male | | | Female | | |
| --- | --- | --- | --- | --- | --- | --- |
|  | 2021 | | EAPC from 1990 to 2021 | 2021 | | EAPC from 1990 to 2021 |
|  | incidence number  (95% UI) | incidence rate  (95% UI) | rate  (95% CI) | incidence number  (95% UI) | incidence rate  (95% UI) | rate  (95% CI) |
| Afghanistan | 13936.65(11267.75,17492.62) | 489.06(395.40,613.84) | -0.54(-0.58,-0.50) | 12221.93(9942.27,15246.42) | 464.40(377.78,579.33) | -0.49(-0.55,-0.42) |
| Albania | 221.14(171.30,289.15) | 298.04(230.86,389.70) | 0.19(0.00,0.38) | 213.43(165.40,282.16) | 312.02(241.80,412.49) | 0.13(-0.10,0.36) |
| Algeria | 6045.79(4849.62,7767.10) | 249.34(200.01,320.33) | 0.07(0.00,0.14) | 5408.43(4405.01,6805.08) | 237.22(193.21,298.48) | 0.07(-0.17,0.32) |
| American Samoa | 5.24(3.95,7.28) | 274.21(206.83,381.55) | -0.53(-0.68,-0.39) | 4.53(3.38,6.25) | 251.77(187.58,347.29) | -0.46(-0.62,-0.29) |
| Andorra | 3.10(2.51,3.89) | 239.97(194.57,301.07) | -0.58(-0.69,-0.47) | 2.92(2.35,3.68) | 237.10(191.00,298.13) | -0.54(-0.80,-0.29) |
| Angola | 12794.01(9830.95,16653.83) | 450.84(346.43,586.85) | -0.95(-1.06,-0.84) | 13142.17(10106.66,17370.44) | 470.14(361.55,621.40) | -1.02(-1.11,-0.94) |
| Antigua and Barbuda | 5.16(3.90,7.51) | 191.90(145.03,279.37) | -0.05(-0.33,0.24) | 5.64(4.17,8.09) | 218.13(161.46,312.93) | -0.04(-0.10,0.01) |
| Argentina | 3145.43(2507.41,4003.02) | 205.45(163.78,261.47) | -0.26(-0.43,-0.10) | 3113.58(2496.07,4068.41) | 212.49(170.35,277.66) | -0.24(-0.37,-0.12) |
| Armenia | 404.79(314.58,533.81) | 412.69(320.72,544.24) | 0.28(-0.13,0.69) | 388.97(299.81,519.83) | 441.39(340.21,589.89) | 0.27(0.01,0.54) |
| Australia | 1350.51(1060.35,1768.78) | 174.65(137.13,228.74) | 0.27(0.17,0.38) | 1316.47(1032.70,1721.37) | 180.29(141.43,235.74) | 0.24(0.12,0.36) |
| Austria | 664.39(562.90,799.45) | 298.21(252.66,358.83) | 0.84(0.59,1.10) | 689.65(590.35,823.29) | 329.47(282.03,393.31) | 0.77(0.57,0.97) |
| Azerbaijan | 1781.45(1394.18,2210.06) | 464.47(363.50,576.22) | 0.18(-0.04,0.39) | 1550.92(1213.74,1993.90) | 459.53(359.63,590.78) | 0.11(-0.21,0.44) |
| Bahamas | 21.18(16.19,29.76) | 197.74(151.16,277.89) | -0.25(-0.38,-0.13) | 22.00(16.67,30.26) | 212.68(161.09,292.51) | -0.23(-0.61,0.14) |
| Bahrain | 86.08(69.02,107.89) | 179.60(144.00,225.12) | -0.39(-0.53,-0.26) | 82.28(64.66,105.87) | 179.80(141.30,231.34) | -0.35(-0.56,-0.13) |
| Bangladesh | 26126.09(19981.37,34388.05) | 354.33(270.99,466.38) | -0.65(-0.69,-0.61) | 22550.08(17155.90,31296.16) | 322.57(245.41,447.69) | -0.67(-0.71,-0.62) |
| Barbados | 12.82(9.66,18.17) | 183.87(138.49,260.62) | -0.33(-0.47,-0.19) | 14.15(10.83,20.21) | 212.96(162.94,304.23) | -0.28(-0.41,-0.15) |
| Belarus | 591.92(443.01,816.78) | 246.12(184.20,339.62) | 0.35(0.08,0.63) | 564.34(425.31,767.96) | 248.76(187.48,338.52) | 0.37(0.00,0.74) |
| Belgium | 694.24(585.89,799.54) | 229.33(193.54,264.12) | -0.47(-0.75,-0.20) | 630.09(536.55,730.03) | 217.86(185.52,252.42) | -0.42(-0.62,-0.22) |
| Belize | 46.15(35.94,62.48) | 239.14(186.26,323.79) | -0.35(-0.44,-0.26) | 47.51(36.34,67.07) | 252.24(192.96,356.10) | -0.28(-0.36,-0.20) |
| Benin | 5340.95(4025.20,7452.61) | 447.46(337.23,624.37) | -0.37(-0.53,-0.21) | 5543.84(4239.67,7395.99) | 482.60(369.07,643.83) | -0.31(-0.35,-0.28) |
| Bermuda | 2.30(1.58,3.34) | 176.41(121.31,255.98) | -0.32(-0.42,-0.22) | 2.54(1.77,4.01) | 203.77(141.77,321.49) | -0.27(-0.37,-0.17) |
| Bhutan | 114.22(88.77,152.94) | 368.40(286.33,493.28) | -0.68(-0.86,-0.49) | 98.62(74.41,132.40) | 328.45(247.82,440.94) | -0.68(-0.72,-0.63) |
| Bolivia (Plurinational State of) | 2686.69(2047.96,3659.10) | 441.04(336.19,600.67) | -0.39(-0.44,-0.33) | 1748.84(1345.03,2476.03) | 298.94(229.91,423.24) | -0.32(-0.38,-0.26) |
| Bosnia and Herzegovina | 193.38(151.16,258.47) | 248.06(193.90,331.55) | -0.13(-0.27,-0.00) | 192.05(149.63,252.95) | 262.79(204.75,346.12) | -0.10(-0.20,-0.00) |
| Botswana | 440.36(331.33,618.52) | 368.45(277.22,517.51) | -0.37(-0.42,-0.31) | 500.80(388.23,662.87) | 431.80(334.74,571.54) | -0.31(-0.35,-0.27) |
| Brazil | 30642.50(22134.74,44469.74) | 361.69(261.27,524.90) | 0.05(-0.13,0.23) | 23396.03(17704.09,32232.88) | 289.36(218.96,398.65) | 0.03(-0.05,0.11) |
| Brunei Darussalam | 46.29(37.82,57.99) | 288.50(235.71,361.42) | 0.17(-0.02,0.37) | 48.02(39.78,59.29) | 323.33(267.88,399.27) | 0.11(-0.04,0.27) |
| Bulgaria | 503.72(390.65,648.21) | 323.71(251.05,416.56) | 0.86(0.60,1.13) | 483.62(383.23,630.48) | 328.83(260.57,428.69) | 0.84(0.51,1.17) |
| Burkina Faso | 10395.17(7896.00,14124.28) | 499.66(379.53,678.91) | -0.36(-0.44,-0.29) | 10975.65(8480.34,14397.22) | 541.73(418.57,710.62) | -0.30(-0.35,-0.26) |
| Burundi | 4596.64(3513.10,6436.35) | 423.34(323.55,592.77) | -0.41(-0.52,-0.31) | 4982.01(3880.84,6513.55) | 463.63(361.15,606.15) | -0.37(-0.42,-0.31) |
| Cabo Verde | 58.33(40.06,86.50) | 260.00(178.57,385.59) | -0.42(-0.50,-0.34) | 60.37(41.89,89.00) | 279.17(193.71,411.55) | -0.38(-0.44,-0.32) |
| Cambodia | 3672.03(2923.66,4794.17) | 410.91(327.17,536.48) | -0.63(-0.77,-0.49) | 3255.13(2531.52,4239.81) | 380.76(296.11,495.93) | -0.59(-0.66,-0.52) |
| Cameroon | 10180.77(7693.99,13962.59) | 409.80(309.70,562.02) | -0.52(-0.62,-0.42) | 10582.23(8073.50,14471.68) | 444.75(339.31,608.22) | -0.45(-0.54,-0.35) |
| Canada | 1264.58(974.73,1701.21) | 129.86(100.10,174.70) | -1.94(-2.32,-1.56) | 1507.94(1136.05,2002.00) | 162.79(122.64,216.13) | -1.59(-1.83,-1.36) |
| Central African Republic | 2434.27(1903.14,3156.87) | 569.58(445.30,738.65) | -0.37(-0.41,-0.33) | 2449.07(1902.96,3224.03) | 595.21(462.49,783.56) | -0.31(-0.36,-0.26) |
| Chad | 9809.68(7485.19,13344.54) | 529.30(403.88,720.03) | -0.46(-0.53,-0.39) | 10358.06(8073.01,13456.67) | 581.20(452.99,755.07) | -0.41(-0.45,-0.37) |
| Chile | 1194.59(1004.65,1444.95) | 215.53(181.26,260.70) | -0.11(-0.14,-0.07) | 1379.83(1193.40,1652.25) | 258.54(223.61,309.59) | -0.07(-0.17,0.04) |
| China | 97322.36(73414.80,131910.80) | 233.78(176.35,316.86) | -1.51(-1.78,-1.24) | 76286.44(58867.30,102470.92) | 211.69(163.35,284.34) | -1.38(-1.75,-1.01) |
| Colombia | 5249.83(3698.65,7979.04) | 297.72(209.75,452.50) | -0.39(-0.51,-0.27) | 3456.24(2631.48,4743.99) | 205.80(156.69,282.47) | -0.35(-0.43,-0.26) |
| Comoros | 147.35(108.82,212.58) | 354.67(261.93,511.68) | -0.63(-0.71,-0.55) | 156.51(116.14,223.06) | 393.62(292.09,560.97) | -0.62(-0.66,-0.57) |
| Congo | 1195.50(882.78,1700.45) | 372.96(275.40,530.50) | -0.82(-0.99,-0.66) | 1231.47(915.41,1773.05) | 394.32(293.12,567.73) | -0.77(-0.93,-0.61) |
| Cook Islands | 1.23(0.87,1.80) | 210.40(148.59,308.78) | -0.56(-0.66,-0.45) | 1.04(0.75,1.54) | 192.76(139.21,285.31) | -0.53(-0.73,-0.33) |
| Costa Rica | 381.05(265.40,553.65) | 242.38(168.82,352.17) | -0.53(-0.58,-0.49) | 231.12(170.41,342.89) | 152.97(112.78,226.94) | -0.46(-0.58,-0.35) |
| Côte d'Ivoire | 9146.96(6945.75,12243.60) | 410.51(311.72,549.49) | -0.42(-0.48,-0.36) | 9679.45(7278.08,13418.97) | 455.24(342.30,631.12) | -0.37(-0.46,-0.27) |
| Croatia | 209.33(165.74,260.59) | 223.31(176.80,278.00) | 0.87(0.36,1.38) | 181.67(137.66,240.50) | 205.26(155.53,271.73) | 0.94(0.62,1.27) |
| Cuba | 502.15(362.98,721.78) | 178.28(128.87,256.26) | -0.04(-0.26,0.18) | 516.31(375.65,794.55) | 196.46(142.94,302.33) | -0.03(-0.28,0.21) |
| Cyprus | 89.57(73.35,111.46) | 231.29(189.41,287.82) | -0.25(-0.45,-0.05) | 83.92(68.97,102.51) | 231.09(189.91,282.28) | -0.23(-0.41,-0.05) |
| Czechia | 616.70(471.98,812.41) | 214.30(164.01,282.30) | 0.36(0.08,0.65) | 575.26(432.76,771.65) | 209.73(157.78,281.33) | 0.39(0.16,0.62) |
| Democratic People's Republic of Korea | 2341.71(1717.57,3191.06) | 302.66(222.00,412.44) | -0.80(-0.97,-0.62) | 2039.80(1505.12,2858.28) | 275.86(203.55,386.56) | -0.73(-0.86,-0.61) |
| Democratic Republic of the Congo | 30396.19(22954.21,41749.81) | 440.24(332.45,604.68) | -0.66(-0.75,-0.58) | 30937.04(23602.20,40792.37) | 464.78(354.59,612.84) | -0.67(-0.78,-0.57) |
| Denmark | 396.96(322.77,489.95) | 249.04(202.49,307.37) | -0.99(-1.05,-0.93) | 367.60(307.10,450.77) | 242.97(202.98,297.94) | -1.07(-1.27,-0.87) |
| Djibouti | 217.55(160.91,312.89) | 283.27(209.53,407.42) | -0.81(-0.93,-0.69) | 214.19(163.20,287.25) | 310.88(236.87,416.91) | -0.74(-0.93,-0.55) |
| Dominica | 3.76(2.88,5.32) | 213.01(163.12,301.14) | -0.46(-0.59,-0.33) | 4.01(3.06,5.69) | 234.47(178.83,332.50) | -0.42(-0.61,-0.22) |
| Dominican Republic | 1432.93(1115.46,1948.69) | 271.59(211.42,369.35) | -0.24(-0.43,-0.05) | 1445.47(1117.29,1992.06) | 285.94(221.02,394.06) | -0.21(-0.32,-0.11) |
| Ecuador | 3083.33(2188.95,4638.87) | 364.04(258.45,547.70) | -0.51(-0.62,-0.40) | 1873.98(1396.62,2694.42) | 230.17(171.54,330.95) | -0.44(-0.60,-0.29) |
| Egypt | 19908.86(16136.84,25218.03) | 297.78(241.36,377.20) | -0.04(-0.32,0.24) | 17935.58(14654.59,22857.53) | 282.37(230.72,359.86) | -0.04(-0.35,0.28) |
| El Salvador | 975.63(715.08,1422.19) | 314.12(230.23,457.90) | -0.54(-0.66,-0.42) | 625.83(487.51,830.89) | 215.70(168.03,286.38) | -0.48(-0.70,-0.26) |
| Equatorial Guinea | 350.00(260.33,488.95) | 352.95(262.52,493.08) | -1.36(-1.40,-1.32) | 360.60(270.92,492.84) | 408.96(307.25,558.94) | -1.24(-1.31,-1.16) |
| Eritrea | 1851.19(1430.85,2488.99) | 390.89(302.14,525.57) | -0.44(-0.63,-0.26) | 1862.99(1418.56,2465.96) | 419.16(319.16,554.82) | -0.40(-0.48,-0.32) |
| Estonia | 88.58(66.43,117.70) | 249.20(186.89,331.12) | 0.55(0.20,0.90) | 86.40(65.01,117.25) | 256.91(193.31,348.64) | 0.49(0.10,0.88) |
| Eswatini | 267.57(202.12,361.30) | 371.98(280.98,502.28) | -0.16(-0.29,-0.03) | 297.73(226.91,391.46) | 434.74(331.34,571.62) | -0.15(-0.26,-0.03) |
| Ethiopia | 35777.43(27580.76,47935.19) | 437.67(337.40,586.40) | -0.87(-0.98,-0.77) | 36968.08(28099.95,49853.54) | 474.52(360.69,639.92) | -0.87(-0.93,-0.81) |
| Fiji | 128.87(98.95,174.91) | 273.98(210.38,371.87) | -0.25(-0.48,-0.01) | 111.76(84.83,156.18) | 253.95(192.74,354.88) | -0.22(-0.41,-0.04) |
| Finland | 308.27(253.25,386.92) | 247.99(203.73,311.26) | -0.72(-0.81,-0.62) | 267.38(214.47,339.22) | 225.65(181.00,286.28) | -0.70(-1.01,-0.38) |
| France | 3955.15(3453.40,4596.57) | 219.21(191.40,254.76) | 0.12(-0.14,0.39) | 3612.38(3090.97,4158.84) | 208.85(178.71,240.45) | 0.09(-0.01,0.18) |
| Gabon | 374.42(269.76,530.70) | 348.01(250.74,493.27) | -0.64(-0.75,-0.54) | 364.18(266.67,526.11) | 343.73(251.70,496.56) | -0.63(-0.69,-0.57) |
| Gambia | 715.89(516.18,1030.36) | 393.20(283.51,565.92) | -0.63(-0.70,-0.56) | 715.29(539.15,1036.28) | 407.52(307.17,590.40) | -0.61(-0.70,-0.51) |
| Georgia | 398.58(317.69,508.87) | 315.93(251.81,403.35) | 0.54(0.19,0.88) | 348.13(284.61,437.25) | 297.22(242.99,373.30) | 0.47(0.21,0.73) |
| Germany | 5357.91(4423.34,6550.34) | 258.25(213.21,315.73) | 0.45(0.24,0.65) | 4693.29(3893.47,5629.90) | 238.39(197.76,285.96) | 0.44(0.28,0.59) |
| Ghana | 8886.77(6616.71,12295.06) | 374.51(278.84,518.14) | -0.21(-0.37,-0.06) | 9277.76(6861.31,13147.90) | 409.21(302.63,579.91) | -0.18(-0.25,-0.11) |
| Greece | 457.09(382.27,556.84) | 210.93(176.40,256.96) | 0.62(0.26,0.99) | 451.26(371.69,563.96) | 219.16(180.51,273.89) | 0.50(0.30,0.71) |
| Greenland | 5.47(4.49,6.70) | 260.33(213.69,318.62) | -0.12(-0.23,-0.01) | 5.30(4.20,6.69) | 273.55(216.72,344.89) | -0.08(-0.24,0.07) |
| Grenada | 7.99(5.99,10.82) | 227.11(170.32,307.56) | -0.04(-0.11,0.03) | 8.34(6.30,11.88) | 249.18(188.21,354.92) | -0.04(-0.14,0.05) |
| Guam | 16.23(11.93,22.90) | 244.80(179.89,345.33) | -0.15(-0.24,-0.06) | 14.39(10.32,20.20) | 234.02(167.87,328.55) | -0.10(-0.41,0.20) |
| Guatemala | 3295.67(2451.68,4765.35) | 416.96(310.18,602.90) | 0.06(-0.02,0.15) | 2328.30(1836.41,3004.18) | 302.82(238.85,390.73) | 0.04(-0.11,0.19) |
| Guinea | 5466.28(4234.06,7507.00) | 475.74(368.50,653.35) | -0.46(-0.59,-0.33) | 5823.61(4536.66,7535.84) | 528.71(411.87,684.16) | -0.41(-0.44,-0.38) |
| Guinea-Bissau | 826.32(631.96,1101.21) | 487.46(372.80,649.62) | -0.55(-0.61,-0.50) | 831.43(635.84,1106.21) | 509.64(389.75,678.07) | -0.50(-0.57,-0.42) |
| Guyana | 108.03(84.70,143.30) | 282.88(221.79,375.23) | -0.24(-0.28,-0.21) | 106.73(82.36,144.60) | 293.85(226.74,398.10) | -0.22(-0.39,-0.04) |
| Haiti | 3466.76(2811.67,4356.32) | 435.59(353.28,547.36) | -0.18(-0.36,-0.00) | 3596.53(2883.89,4533.12) | 464.68(372.61,585.69) | -0.16(-0.21,-0.11) |
| Honduras | 1981.22(1475.43,2850.70) | 354.28(263.84,509.76) | -0.26(-0.30,-0.22) | 1442.22(1127.60,1919.43) | 268.92(210.25,357.90) | -0.24(-0.30,-0.18) |
| Hungary | 622.82(475.09,828.30) | 267.20(203.82,355.35) | 0.17(0.08,0.27) | 608.57(460.28,823.71) | 274.98(207.97,372.19) | 0.11(-0.06,0.29) |
| Iceland | 28.40(22.92,36.03) | 251.68(203.10,319.26) | 0.09(-0.02,0.21) | 22.05(18.05,27.54) | 206.31(168.88,257.72) | 0.09(-0.13,0.31) |
| India | 210429.62(163461.76,275361.90) | 360.67(280.17,471.96) | -0.12(-0.28,0.03) | 203075.62(160723.84,262506.23) | 383.22(303.30,495.37) | -0.09(-0.22,0.04) |
| Indonesia | 41217.46(32027.39,54062.79) | 367.00(285.17,481.37) | -0.40(-0.46,-0.34) | 36386.27(28122.04,47393.47) | 340.84(263.42,443.94) | -0.36(-0.41,-0.31) |
| Iran (Islamic Republic of) | 7287.06(5899.50,9078.43) | 230.17(186.34,286.75) | 0.19(0.04,0.34) | 7048.92(5677.50,8849.74) | 235.82(189.94,296.06) | 0.12(-0.17,0.41) |
| Iraq | 6609.96(5260.64,8319.13) | 298.86(237.85,376.14) | -0.50(-0.69,-0.31) | 5811.57(4675.60,7179.85) | 279.17(224.60,344.90) | -0.43(-0.53,-0.33) |
| Ireland | 349.25(300.13,413.00) | 228.40(196.28,270.10) | -0.25(-0.46,-0.04) | 313.73(268.66,369.99) | 215.64(184.66,254.30) | -0.22(-0.57,0.12) |
| Israel | 948.46(771.68,1196.98) | 201.20(163.70,253.92) | -0.16(-0.24,-0.07) | 893.84(724.04,1107.89) | 200.02(162.02,247.92) | -0.14(-0.30,0.03) |
| Italy | 2262.43(1837.26,2834.35) | 202.89(164.76,254.18) | -0.29(-0.49,-0.08) | 2211.52(1798.89,2716.60) | 209.61(170.50,257.49) | -0.25(-0.33,-0.16) |
| Jamaica | 170.14(128.51,238.79) | 195.53(147.69,274.43) | -0.02(-0.18,0.14) | 182.84(139.87,259.44) | 217.23(166.17,308.23) | -0.02(-0.13,0.09) |
| Japan | 6058.20(4963.51,7574.84) | 257.56(211.02,322.04) | -0.36(-0.38,-0.33) | 6127.53(4994.98,7726.70) | 274.37(223.66,345.98) | -0.29(-0.46,-0.11) |
| Jordan | 1277.71(1008.92,1676.40) | 226.39(178.77,297.04) | -0.74(-0.79,-0.68) | 1080.35(852.08,1414.52) | 202.71(159.88,265.42) | -0.70(-0.79,-0.62) |
| Kazakhstan | 4548.13(3590.32,5777.95) | 452.83(357.46,575.27) | 0.53(0.38,0.69) | 4339.26(3363.15,5687.14) | 459.64(356.25,602.42) | 0.46(0.11,0.82) |
| Kenya | 10300.34(7598.58,14241.90) | 340.61(251.27,470.95) | -0.56(-0.68,-0.43) | 10474.21(7856.32,14409.05) | 357.96(268.49,492.43) | -0.53(-0.57,-0.48) |
| Kiribati | 29.64(23.39,38.73) | 399.82(315.59,522.58) | -0.36(-0.43,-0.30) | 25.70(20.12,33.76) | 373.55(292.42,490.72) | -0.29(-0.39,-0.20) |
| Kuwait | 252.43(198.58,330.65) | 185.47(145.91,242.94) | -0.14(-0.26,-0.02) | 229.24(179.88,298.85) | 177.83(139.54,231.83) | -0.10(-0.45,0.25) |
| Kyrgyzstan | 1790.87(1387.10,2324.29) | 439.76(340.61,570.75) | 0.24(-0.00,0.48) | 1774.68(1387.37,2279.71) | 458.97(358.81,589.59) | 0.22(-0.06,0.50) |
| Lao People's Democratic Republic | 1974.35(1535.09,2539.35) | 466.66(362.84,600.20) | -0.74(-1.04,-0.44) | 1760.26(1390.89,2274.84) | 433.08(342.20,559.69) | -0.72(-0.82,-0.63) |
| Latvia | 121.71(93.85,159.86) | 251.18(193.69,329.91) | 0.75(0.53,0.98) | 118.26(86.95,164.79) | 261.30(192.11,364.10) | 0.73(0.33,1.12) |
| Lebanon | 422.36(339.23,537.40) | 200.04(160.67,254.52) | -0.63(-0.70,-0.56) | 371.74(302.79,485.76) | 190.87(155.47,249.42) | -0.61(-0.78,-0.44) |
| Lesotho | 419.79(323.50,585.96) | 408.52(314.82,570.25) | -0.19(-0.23,-0.14) | 450.99(352.74,600.74) | 446.71(349.40,595.04) | -0.16(-0.19,-0.14) |
| Liberia | 1679.27(1249.69,2411.92) | 427.94(318.47,614.65) | -0.95(-1.09,-0.80) | 1701.48(1331.08,2205.93) | 454.96(355.92,589.85) | -1.00(-1.07,-0.92) |
| Libya | 512.48(411.74,660.62) | 236.69(190.16,305.11) | -0.43(-0.54,-0.31) | 450.88(365.89,570.00) | 218.28(177.14,275.95) | -0.38(-0.44,-0.32) |
| Lithuania | 166.18(124.13,224.28) | 246.04(183.78,332.05) | 0.70(0.42,0.98) | 161.48(118.51,225.40) | 252.11(185.02,351.90) | 0.59(0.26,0.92) |
| Luxembourg | 36.60(29.60,46.18) | 216.82(175.36,273.56) | -0.52(-0.64,-0.40) | 35.41(28.85,43.79) | 218.81(178.27,270.59) | -0.45(-0.57,-0.32) |
| Madagascar | 8109.60(6178.23,11262.60) | 390.58(297.56,542.44) | -0.54(-0.64,-0.45) | 8658.32(6605.38,11532.29) | 430.38(328.33,573.24) | -0.49(-0.52,-0.46) |
| Malawi | 5458.72(4138.35,7204.86) | 397.86(301.62,525.12) | -1.22(-1.31,-1.13) | 5690.73(4402.26,7690.93) | 420.90(325.60,568.84) | -1.15(-1.23,-1.08) |
| Malaysia | 2820.78(1999.22,3991.96) | 222.37(157.61,314.70) | -0.44(-0.60,-0.28) | 2470.85(1759.01,3573.38) | 207.65(147.83,300.31) | -0.40(-0.56,-0.24) |
| Maldives | 40.34(29.72,56.83) | 247.41(182.32,348.56) | -0.49(-0.62,-0.35) | 38.50(28.46,54.72) | 249.09(184.15,354.04) | -0.42(-0.59,-0.25) |
| Mali | 11756.27(8942.18,16195.72) | 504.23(383.54,694.64) | -0.62(-0.80,-0.45) | 12430.56(9610.73,16343.19) | 552.75(427.36,726.73) | -0.56(-0.59,-0.52) |
| Malta | 26.98(22.51,34.10) | 237.19(197.85,299.71) | 1.33(1.07,1.60) | 25.22(20.84,31.26) | 237.36(196.11,294.20) | 1.16(0.93,1.39) |
| Marshall Islands | 9.83(7.45,13.26) | 335.11(254.15,452.21) | -0.15(-0.27,-0.02) | 8.33(6.41,11.16) | 303.80(233.81,406.91) | -0.11(-0.19,-0.02) |
| Mauritania | 1160.74(844.57,1653.92) | 346.49(252.11,493.70) | -0.71(-0.76,-0.66) | 1194.11(887.28,1686.79) | 370.84(275.55,523.85) | -0.69(-0.72,-0.66) |
| Mauritius | 73.42(52.62,102.70) | 224.71(161.05,314.31) | -0.18(-0.24,-0.12) | 63.91(45.99,89.92) | 202.11(145.44,284.37) | -0.16(-0.38,0.05) |
| Mexico | 17377.03(12912.34,24560.42) | 347.71(258.37,491.45) | -0.40(-0.42,-0.37) | 11698.46(9233.26,15193.99) | 239.74(189.22,311.38) | -0.35(-0.51,-0.19) |
| Micronesia (Federated States of) | 15.18(11.70,21.02) | 310.22(239.04,429.53) | -0.22(-0.27,-0.16) | 13.04(9.79,18.57) | 284.83(213.88,405.75) | -0.19(-0.24,-0.15) |
| Monaco | 1.71(1.40,2.13) | 209.16(171.14,260.70) | -1.41(-1.99,-0.82) | 1.51(1.22,1.88) | 187.79(152.19,233.73) | -1.32(-1.40,-1.24) |
| Mongolia | 1103.53(875.12,1426.69) | 549.72(435.94,710.70) | -0.08(-0.19,0.02) | 1047.43(843.38,1323.04) | 551.42(443.99,696.51) | -0.04(-0.28,0.20) |
| Montenegro | 44.35(33.83,58.75) | 236.00(180.04,312.61) | 0.35(0.10,0.60) | 43.59(33.45,59.30) | 250.54(192.24,340.78) | 0.34(0.23,0.45) |
| Morocco | 4798.47(3848.37,6025.41) | 288.16(231.10,361.84) | -0.48(-0.61,-0.35) | 4275.24(3425.77,5311.84) | 269.63(216.06,335.01) | -0.42(-0.52,-0.31) |
| Mozambique | 10758.67(8447.07,14233.92) | 411.93(323.42,544.99) | -0.75(-0.82,-0.69) | 11428.80(8933.36,15401.37) | 445.14(347.95,599.87) | -0.73(-0.75,-0.70) |
| Myanmar | 11473.91(8993.42,14845.78) | 429.35(336.53,555.52) | -0.63(-0.67,-0.59) | 9809.46(7466.14,13133.33) | 384.08(292.33,514.22) | -0.59(-0.67,-0.52) |
| Namibia | 490.11(358.41,722.97) | 349.02(255.24,514.86) | -0.40(-0.51,-0.29) | 540.84(416.28,738.43) | 391.83(301.59,534.98) | -0.35(-0.44,-0.27) |
| Nauru | 2.59(2.00,3.53) | 354.41(273.91,482.44) | -0.41(-0.59,-0.22) | 2.13(1.63,2.90) | 319.86(244.80,435.68) | -0.36(-0.46,-0.27) |
| Nepal | 6099.14(4688.37,8276.80) | 380.25(292.30,516.01) | -0.82(-0.90,-0.74) | 4471.33(3362.76,6158.34) | 297.61(223.82,409.90) | -0.76(-0.93,-0.60) |
| Netherlands | 894.55(760.39,1083.99) | 202.83(172.41,245.78) | -0.71(-0.86,-0.56) | 834.98(712.00,998.39) | 198.72(169.45,237.61) | -0.69(-0.85,-0.54) |
| New Zealand | 345.82(282.72,439.97) | 215.48(176.17,274.15) | 0.07(-0.17,0.31) | 330.54(273.31,413.38) | 217.30(179.67,271.75) | 0.07(-0.07,0.20) |
| Nicaragua | 1034.70(771.04,1500.71) | 309.95(230.97,449.55) | -0.17(-0.31,-0.04) | 704.49(554.32,918.25) | 222.44(175.02,289.94) | -0.15(-0.20,-0.09) |
| Niger | 13509.85(10356.93,17938.05) | 519.94(398.60,690.36) | -0.84(-0.87,-0.82) | 14398.01(11323.90,18788.01) | 576.39(453.32,752.13) | -0.81(-0.94,-0.69) |
| Nigeria | 95085.77(73143.33,125651.07) | 507.83(390.64,671.07) | -0.60(-0.73,-0.47) | 97025.43(75968.95,126810.80) | 527.49(413.02,689.42) | -0.55(-0.63,-0.47) |
| Niue | 0.16(0.12,0.22) | 271.37(202.95,365.56) | -0.04(-0.10,0.02) | 0.13(0.09,0.18) | 227.75(163.60,324.04) | -0.03(-0.19,0.13) |
| North Macedonia | 130.60(101.17,168.96) | 251.25(194.65,325.07) | -0.22(-0.42,-0.02) | 130.31(99.76,169.74) | 268.46(205.52,349.69) | -0.18(-0.28,-0.09) |
| Northern Mariana Islands | 3.28(2.36,4.73) | 197.42(142.28,284.28) | -1.26(-1.62,-0.90) | 2.89(2.09,4.17) | 186.90(135.29,270.04) | -1.24(-1.82,-0.65) |
| Norway | 363.14(295.86,451.23) | 251.49(204.90,312.50) | -0.63(-0.74,-0.53) | 332.43(271.91,410.58) | 243.14(198.87,300.30) | -0.62(-0.79,-0.46) |
| Oman | 472.56(380.89,614.98) | 218.42(176.05,284.25) | -0.43(-0.64,-0.21) | 462.34(371.27,587.50) | 222.63(178.78,282.90) | -0.38(-0.51,-0.26) |
| Pakistan | 68201.11(53960.34,89577.07) | 445.50(352.48,585.13) | -0.23(-0.31,-0.15) | 55841.65(43634.20,73536.88) | 387.33(302.66,510.07) | -0.20(-0.27,-0.13) |
| Palau | 1.28(0.95,1.78) | 258.37(191.62,359.50) | -0.44(-0.47,-0.41) | 1.12(0.83,1.55) | 247.49(183.36,343.00) | -0.40(-0.55,-0.25) |
| Palestine | 760.86(602.88,962.64) | 242.15(191.87,306.37) | -0.46(-0.50,-0.41) | 677.31(546.73,875.69) | 226.87(183.13,293.32) | -0.41(-0.51,-0.31) |
| Panama | 538.83(388.59,786.11) | 282.34(203.61,411.90) | -0.20(-0.37,-0.02) | 343.01(260.03,477.13) | 190.16(144.16,264.51) | -0.17(-0.30,-0.03) |
| Papua New Guinea | 3622.96(2808.42,4741.09) | 455.74(353.28,596.39) | 0.14(0.07,0.21) | 3088.86(2391.18,4073.68) | 425.26(329.20,560.84) | 0.10(0.02,0.17) |
| Paraguay | 1022.42(700.67,1561.83) | 306.20(209.84,467.74) | -0.09(-0.36,0.18) | 768.76(575.68,1094.09) | 243.15(182.09,346.06) | -0.04(-0.10,0.01) |
| Peru | 6610.37(4695.76,10353.84) | 388.22(275.77,608.06) | -0.32(-0.37,-0.27) | 4023.78(3004.66,6008.84) | 251.84(188.06,376.08) | -0.27(-0.36,-0.19) |
| Philippines | 20208.77(15752.69,26317.94) | 346.93(270.43,451.80) | -0.31(-0.40,-0.22) | 16951.66(13056.59,22822.19) | 314.60(242.31,423.55) | -0.27(-0.36,-0.17) |
| Poland | 2091.39(1733.94,2600.18) | 216.43(179.44,269.08) | -0.12(-0.23,0.00) | 2518.88(2031.10,3242.86) | 275.06(221.80,354.12) | -0.08(-0.28,0.12) |
| Portugal | 394.80(318.97,485.06) | 181.86(146.93,223.43) | 0.56(0.31,0.82) | 373.12(303.14,458.30) | 179.18(145.58,220.09) | 0.50(0.22,0.78) |
| Puerto Rico | 86.80(64.81,123.53) | 160.98(120.20,229.11) | -0.95(-1.08,-0.82) | 90.46(66.37,135.62) | 176.67(129.62,264.86) | -1.03(-1.28,-0.78) |
| Qatar | 188.90(150.96,246.14) | 202.02(161.44,263.23) | -0.25(-0.37,-0.13) | 171.09(137.41,219.31) | 188.43(151.34,241.54) | -0.23(-0.45,-0.01) |
| Republic of Korea | 1545.39(1235.25,1935.33) | 195.14(155.97,244.37) | -1.10(-1.19,-1.01) | 1640.19(1348.24,2020.65) | 216.40(177.88,266.60) | -1.07(-1.35,-0.80) |
| Republic of Moldova | 221.26(171.75,289.47) | 278.68(216.32,364.59) | 0.31(0.07,0.55) | 211.03(161.61,277.36) | 282.03(215.98,370.68) | 0.33(0.06,0.60) |
| Romania | 1337.41(1050.96,1684.48) | 277.85(218.34,349.95) | 0.46(0.06,0.86) | 1288.55(1000.53,1668.67) | 282.26(219.17,365.53) | 0.44(0.22,0.66) |
| Russian Federation | 10346.03(8194.08,13288.39) | 264.68(209.63,339.96) | 0.38(0.22,0.53) | 9931.00(7707.46,12789.09) | 268.27(208.21,345.48) | 0.39(-0.05,0.82) |
| Rwanda | 3447.24(2565.30,4703.98) | 388.03(288.76,529.49) | -0.91(-1.19,-0.63) | 3614.78(2754.33,5100.27) | 420.29(320.25,593.01) | -0.88(-0.98,-0.77) |
| Saint Kitts and Nevis | 3.35(2.57,4.68) | 216.27(166.26,302.20) | -0.21(-0.35,-0.06) | 3.48(2.59,4.89) | 231.59(172.35,325.04) | -0.18(-0.32,-0.03) |
| Saint Lucia | 9.26(7.06,13.43) | 205.13(156.41,297.44) | -0.01(-0.35,0.33) | 9.74(7.37,13.64) | 225.53(170.73,315.77) | -0.02(-0.11,0.08) |
| Saint Vincent and the Grenadines | 7.46(5.60,10.06) | 204.07(153.06,275.25) | -0.27(-0.41,-0.13) | 8.16(6.26,11.43) | 230.47(176.66,322.76) | -0.24(-0.40,-0.09) |
| Samoa | 45.27(33.77,61.06) | 298.15(222.44,402.16) | -0.15(-0.41,0.11) | 39.54(29.61,56.75) | 281.21(210.55,403.59) | -0.12(-0.17,-0.06) |
| San Marino | 1.42(1.14,1.80) | 225.74(180.99,285.21) | 0.01(-0.13,0.15) | 1.30(1.05,1.65) | 225.11(180.74,284.83) | -0.00(-0.07,0.07) |
| Sao Tome and Principe | 41.72(30.30,59.53) | 327.49(237.84,467.31) | -0.83(-0.98,-0.68) | 41.57(31.12,57.95) | 340.62(255.01,474.79) | -0.80(-0.92,-0.68) |
| Saudi Arabia | 2528.37(2053.89,3172.11) | 201.24(163.48,252.48) | -0.38(-0.44,-0.32) | 2356.16(1917.81,2982.46) | 200.23(162.98,253.45) | -0.32(-0.45,-0.19) |
| Senegal | 4524.67(3372.89,6260.26) | 389.01(289.98,538.23) | -0.48(-0.54,-0.42) | 4681.74(3565.88,6446.20) | 422.42(321.74,581.62) | -0.42(-0.52,-0.32) |
| Serbia | 422.52(332.59,551.00) | 221.38(174.26,288.69) | -0.58(-0.69,-0.47) | 405.82(316.11,531.88) | 228.25(177.79,299.15) | -0.53(-0.72,-0.34) |
| Seychelles | 9.89(7.24,14.21) | 245.79(179.95,353.23) | -0.19(-0.54,0.17) | 8.58(5.99,11.98) | 222.65(155.40,311.00) | -0.17(-0.24,-0.10) |
| Sierra Leone | 3321.16(2583.99,4389.40) | 488.74(380.26,645.94) | -0.65(-0.68,-0.62) | 3535.98(2781.09,4578.20) | 533.00(419.21,690.10) | -0.67(-0.72,-0.62) |
| Singapore | 215.90(174.36,272.00) | 147.69(119.27,186.06) | -0.95(-1.16,-0.75) | 259.33(211.68,330.43) | 185.57(151.47,236.46) | -1.03(-1.26,-0.80) |
| Slovakia | 367.33(278.46,478.31) | 250.31(189.75,325.93) | 0.47(0.09,0.86) | 343.52(263.63,453.31) | 246.46(189.15,325.23) | 0.45(0.27,0.63) |
| Slovenia | 106.03(80.21,138.67) | 210.36(159.13,275.11) | 0.25(0.13,0.36) | 104.73(80.13,141.55) | 220.50(168.71,298.04) | 0.24(-0.00,0.47) |
| Solomon Islands | 190.62(149.79,245.53) | 383.27(301.17,493.67) | -0.50(-0.63,-0.37) | 161.23(124.87,218.95) | 353.55(273.82,480.13) | -0.42(-0.44,-0.40) |
| Somalia | 10621.49(8319.30,14218.96) | 496.88(389.19,665.18) | -0.43(-0.62,-0.24) | 10863.86(8546.66,14460.16) | 545.52(429.17,726.11) | -0.40(-0.45,-0.34) |
| South Africa | 8577.98(6381.40,11892.63) | 341.32(253.92,473.21) | -0.35(-0.44,-0.25) | 9128.44(6931.63,12370.86) | 372.87(283.14,505.32) | -0.28(-0.41,-0.16) |
| South Sudan | 3946.29(3005.68,5493.02) | 488.83(372.31,680.42) | -0.21(-0.25,-0.17) | 4089.23(3167.41,5436.68) | 541.60(419.51,720.06) | -0.18(-0.22,-0.13) |
| Spain | 2059.77(1837.57,2311.94) | 217.69(194.20,244.34) | 0.50(0.09,0.91) | 2050.10(1786.13,2311.48) | 229.05(199.55,258.25) | 0.45(0.38,0.53) |
| Sri Lanka | 1789.31(1290.37,2529.90) | 224.35(161.79,317.22) | -0.65(-0.71,-0.58) | 1554.29(1125.57,2136.93) | 202.36(146.55,278.22) | -0.64(-0.75,-0.52) |
| Sudan | 11292.32(9216.52,13921.80) | 387.91(316.60,478.23) | -0.90(-1.04,-0.76) | 9758.55(7907.40,12115.14) | 357.72(289.86,444.11) | -0.87(-0.91,-0.83) |
| Suriname | 60.81(46.75,86.30) | 267.34(205.54,379.43) | -0.24(-0.29,-0.19) | 60.55(47.17,83.25) | 277.87(216.46,382.04) | -0.21(-0.29,-0.14) |
| Sweden | 569.91(448.70,727.96) | 190.17(149.72,242.91) | 0.51(0.23,0.80) | 487.68(393.17,613.06) | 172.02(138.68,216.24) | 0.46(0.16,0.76) |
| Switzerland | 592.69(502.83,709.93) | 261.07(221.49,312.72) | -0.82(-0.91,-0.72) | 506.25(420.58,614.02) | 235.49(195.64,285.62) | -0.75(-0.80,-0.71) |
| Syrian Arab Republic | 1243.52(1000.57,1603.84) | 241.16(194.04,311.03) | -0.37(-0.49,-0.26) | 1116.99(901.08,1427.34) | 228.23(184.11,291.64) | -0.32(-0.43,-0.21) |
| Taiwan (Province of China) | 883.47(601.52,1313.31) | 191.42(130.33,284.55) | -0.31(-0.47,-0.15) | 761.15(541.80,1089.90) | 176.72(125.80,253.05) | -0.26(-0.53,0.01) |
| Tajikistan | 4026.86(3146.75,5119.83) | 582.53(455.21,740.64) | 0.23(-0.07,0.54) | 3822.26(2985.05,4828.72) | 590.57(461.21,746.08) | 0.20(0.03,0.36) |
| Thailand | 3671.29(2705.46,5034.43) | 252.52(186.09,346.27) | -0.11(-0.18,-0.04) | 3129.12(2305.94,4445.15) | 227.88(167.93,323.72) | -0.08(-0.13,-0.02) |
| Timor-Leste | 405.35(318.20,528.72) | 425.60(334.10,555.14) | -0.94(-1.01,-0.87) | 366.42(289.52,480.04) | 408.92(323.10,535.72) | -0.89(-1.04,-0.74) |
| Togo | 2350.23(1740.40,3367.91) | 394.47(292.12,565.28) | -0.49(-0.60,-0.38) | 2522.08(1890.21,3625.64) | 437.32(327.75,628.67) | -0.42(-0.52,-0.32) |
| Tokelau | 0.12(0.09,0.16) | 226.59(165.87,316.78) | -0.54(-0.56,-0.52) | 0.11(0.08,0.16) | 231.75(170.27,329.62) | -0.49(-0.76,-0.21) |
| Tonga | 21.21(15.90,30.36) | 281.58(211.04,402.99) | -0.11(-0.21,-0.00) | 17.90(13.20,24.74) | 260.43(191.99,359.93) | -0.07(-0.16,0.02) |
| Trinidad and Tobago | 83.63(62.47,117.64) | 203.36(151.91,286.06) | 0.15(0.02,0.29) | 84.91(63.65,122.80) | 215.56(161.59,311.76) | 0.11(-0.04,0.26) |
| Tunisia | 1001.91(810.32,1248.07) | 216.01(174.71,269.09) | -0.10(-0.43,0.22) | 860.15(687.27,1095.57) | 200.87(160.50,255.85) | -0.06(-0.23,0.11) |
| Türkiye | 7323.00(5943.37,9390.04) | 256.83(208.44,329.32) | -0.53(-0.63,-0.44) | 6103.16(4963.58,7784.78) | 226.00(183.80,288.27) | -0.46(-0.55,-0.38) |
| Turkmenistan | 1387.99(1104.25,1777.87) | 501.44(398.94,642.30) | 0.05(-0.13,0.22) | 1343.16(1050.38,1732.02) | 510.87(399.51,658.77) | 0.02(-0.08,0.12) |
| Tuvalu | 2.42(1.88,3.14) | 358.27(279.33,466.34) | -0.59(-0.69,-0.49) | 2.07(1.60,2.73) | 339.00(261.93,448.22) | -0.55(-0.70,-0.41) |
| Uganda | 14491.36(10924.57,19815.92) | 388.25(292.69,530.91) | -0.71(-0.79,-0.62) | 15148.64(11329.26,21125.06) | 422.52(315.99,589.22) | -0.69(-0.75,-0.63) |
| Ukraine | 2349.70(1833.02,3041.77) | 286.52(223.51,370.91) | 0.40(0.09,0.71) | 2221.07(1689.08,2906.45) | 287.66(218.76,376.43) | 0.39(-0.02,0.80) |
| United Arab Emirates | 363.55(295.07,464.89) | 164.17(133.25,209.93) | -0.12(-0.34,0.09) | 332.67(263.02,436.38) | 157.49(124.52,206.59) | -0.09(-0.38,0.20) |
| United Kingdom | 3707.99(3066.32,4612.13) | 198.34(164.01,246.70) | 0.71(0.36,1.06) | 3406.50(2805.83,4159.77) | 190.83(157.18,233.03) | 0.70(0.43,0.96) |
| United Republic of Tanzania | 16432.88(12375.00,22944.78) | 368.21(277.28,514.12) | -0.73(-0.76,-0.70) | 17796.98(13559.24,24153.44) | 406.02(309.34,551.04) | -0.70(-0.75,-0.65) |
| United States of America | 23954.35(19252.29,30411.35) | 252.02(202.55,319.96) | -0.19(-0.27,-0.12) | 22104.54(18060.27,28105.43) | 243.19(198.70,309.21) | -0.17(-0.30,-0.03) |
| United States Virgin Islands | 4.03(3.03,5.64) | 197.87(148.53,276.84) | -0.03(-0.26,0.20) | 4.27(3.07,6.21) | 226.90(163.10,330.16) | -0.03(-0.07,0.02) |
| Uruguay | 217.07(169.83,278.38) | 218.06(170.61,279.65) | -0.25(-0.43,-0.07) | 203.04(158.66,260.42) | 214.96(167.97,275.71) | -0.23(-0.44,-0.02) |
| Uzbekistan | 9991.29(7808.42,12678.45) | 500.82(391.40,635.51) | 0.29(0.09,0.48) | 9410.20(7490.12,12045.34) | 511.36(407.02,654.55) | 0.29(0.15,0.43) |
| Vanuatu | 74.77(57.27,100.37) | 343.05(262.73,460.46) | -0.45(-0.51,-0.40) | 65.93(50.47,86.40) | 323.91(247.95,424.46) | -0.40(-0.44,-0.36) |
| Venezuela (Bolivarian Republic of) | 3333.67(2409.60,5190.66) | 298.42(215.70,464.65) | -0.13(-0.23,-0.02) | 2086.19(1618.15,2747.63) | 195.37(151.54,257.31) | -0.09(-0.35,0.17) |
| Viet Nam | 10645.21(7827.65,14782.00) | 251.43(184.88,349.14) | -0.34(-0.44,-0.24) | 8687.49(6498.05,12235.98) | 222.30(166.28,313.10) | -0.28(-0.40,-0.16) |
| Yemen | 9238.85(7388.62,11609.57) | 383.26(306.50,481.60) | -0.53(-0.68,-0.37) | 8528.91(6957.96,10615.69) | 372.98(304.28,464.24) | -0.45(-0.60,-0.30) |
| Zambia | 5460.39(4128.97,7648.39) | 370.74(280.34,519.30) | -0.87(-1.13,-0.61) | 5892.52(4521.09,7904.52) | 405.76(311.32,544.31) | -0.87(-0.97,-0.76) |
| Zimbabwe | 4312.38(3259.84,5984.61) | 387.04(292.58,537.13) | 0.05(-0.02,0.12) | 4758.36(3721.92,6237.58) | 434.34(339.74,569.37) | 0.03(-0.08,0.14) |

DALYs = disability-adjusted life years; EAPC = estimated annual percentage change.
