# Supplementary material for: Sex differences in global burden of Congenital Heart Anomalies in children under five from 1990 to 2021
Source: PLoS One. 2026 May 6;21(5):e0348351. doi: 10.1371/journal.pone.0348351 (PMC13148693; doi:10.1371/journal.pone.0348351)
Supplement: S10 Table — (DOCX) [file pone.0348351.s010.docx]

**Supplementary Table 10** Results of multivariable linear mixed-effects models examining the association between sex and CHA burden, adjusting for SDI and temporal trends

| Model Outcome | Fixed Effect | Adjusted Coefficient (β) | 95% CI | P-value |
| --- | --- | --- | --- | --- |
| DALYs Rate | Male Sex  (Ref: Female) | 876.40 | 793.90 – 958.90 | < 0.001 |
| Mortality Rate | Male Sex  (Ref: Female) | 9.70 | 8.77 – 10.62 | < 0.001 |

Note: Models were adjusted for Socio-demographic Index (SDI) and year (1990–2021) as fixed effects, with GBD region included as a random effect to account for geographical clustering.
